# The unique immune ecosystems in pediatric brain tumors: integrating single-cell and bulk RNA-sequencing

**DOI:** 10.3389/fimmu.2023.1238684

**Published:** 2023-11-29

**Authors:** Liangliang Cao, Wanqun Xie, Wenkun Ma, Heng Zhao, Jiajia Wang, Zhuangzhuang Liang, Shuaiwei Tian, Baocheng Wang, Jie Ma

**Affiliations:** Department of Pediatric Neurosurgery, Xinhua Hospital Affiliated to Shanghai Jiao Tong University School of Medicine, Shanghai, China

**Keywords:** pediatrics, brain tumors, tumor microenvironment, single-cell RNA-seq, immunotherapy

## Abstract

**Background:**

The significant progress of immune therapy in non-central nervous system tumors has sparked interest in employing the same strategy for adult brain tumors. However, the advancement of immunotherapy in pediatric central nervous system (CNS) tumors is not yet on par. Currently, there is a lack of comprehensive comparative studies investigating the immune ecosystem in pediatric and adult CNS tumors at a high-resolution single-cell level.

**Methods:**

In this study, we comprehensively analyzed over 0.3 million cells from 171 samples, encompassing adult gliomas (IDH wild type and IDH mutation) as well as four major types of pediatric brain tumors (medulloblastoma (MB), ependymoma (EPN), H3K27M-mutation (DIPG), and pediatric IDH-mutation glioma (P-IDH-M)). Our approach involved integrating publicly available and newly generated single-cell datasets. We compared the immune landscapes in different brain tumors, as well as the detailed functional phenotypes of T-cell and myeloid subpopulations. Through single-cell analysis, we identified gene sets associated with major cell types in the tumor microenvironment (gene features from single-cell data, scFes) and compared them with existing gene sets such as GSEA and xCell. The CBTTC and external GEO cohort was used to analyze and validate the immune-stromal-tumor patterns in pediatric brain tumors which might potentially respond to the immunotherapy.

**Results:**

From the perspective of single-cell analysis, it was observed that major pediatric brain tumors (MB, EPN, P-IDH-M, DIPG) exhibited lower immune contents compared with adult gliomas. Additionally, these pediatric brain tumors displayed diverse immunophenotypes, particularly in regard to myeloid cells. Notably, the presence of HLA-enriched myeloid cells in MB was found to be independently associated with prognosis. Moreover, the scFes, when compared with commonly used gene features, demonstrated superior performance in independent single-cell datasets across various tumor types. Furthermore, our study revealed the existence of heterogeneous immune ecosystems at the bulk-RNA sequencing level among different brain tumor types. In addition, we identified several immune-stromal-tumor patterns that could potentially exhibit significant responses to conventional immune checkpoint inhibitors.

**Conclusion:**

The single-cell technique provides a rational path to deeply understand the unique immune ecosystem of pediatric brain tumors. In spite of the traditional attitudes of “cold” tumor towards pediatric brain tumor, the immune-stroma-tumor patterns identified in this study suggest the feasibility of immune checkpoint inhibitors and pave the way for the upcoming tide of immunotherapy in pediatric brain tumors.

## 1 Introduction

The therapeutic strategy targeting the specific component in the immune ecosystem has achieved remarkable advances in recent years (1, 2). Considering the distinct immune microenvironment and the molecular and immunological characters of pediatric brain tumors, it should be more rigorous to apply the scientific findings of their adult counterparts in them (3–5). Recent studies have shown that there are significant differences in immune compositions between children and adults at the levels of bulk RNA and DNA methylation. However, these deconvolution-based methods are unable to directly measure and achieve a high-resolution depiction of the immune composition landscape (6). The direct and systematic mapping of immune ecosystems in pediatric brain tumors, including the detailed immunophenotypes of immune cells, is still lacking. Therefore, a full understanding of the tumor microenvironment (TME) compositions of the CNS at the single-cell level is the essential precondition of the successful application of immunotherapy.

As the major immune components infiltrating into the TME, myeloid cells play important roles in modulating the antitumor functions (7). Some therapeutic strategies redirecting them are ongoing. In order to clearly understand their various functional phenotypes among different cancer types, Zhang et al. systematically investigated the unique and recurrent phenotypes of myeloid across 15 tumor types and identified some potential targets, for example, LAPM3 cDCs and TNF+ mast cells. The CNS hosts the heterogeneous populations of myeloid cells, including microglia and border-associated macrophage. It is conceivable that the functions of microglia are distinct and highly diverse in different ages and pathological conditions (8–10). Klemm et al. found that microglial (MG) and bone marrow-derived myeloid (BMDM) exhibited a multifaceted polarization phenotype and diverse transcriptional programming in adult gliomas and brain metastases (4) and acquired tumor-associated signatures with the dysregulations of hypoxia and inflammatory molecules (9, 11). Recently, the mystery of the TME in pediatric brain tumors was unveiled. The TME of medulloblastoma (MB) was analyzed systematically, and several myeloid clusters were identified (12). In addition, the polarization characters (M1/M2) and prognostic value in MB were investigated by multiple fluorescence immunohistochemistry (13). However, the identification of recurrent functional phenotypes spanning multiple pediatric brain tumor types is still lacking, which will undoubtedly affect the fully understanding of heterogeneity and evolution of the TME.

Different from depicting the state of certain cell type, the systematical identification of constant and specific immune cell pairings of immune, stromal, and tumor cells across the diverse tumor types will provide priori knowledge for cancer immunity before immunotherapy (14). Krummel et al. identified 12 immune archetypes in over 10 tumor types with 10 immune cell features, mainly focusing on non-CNS cancer types (15). Considering the unique immune characteristics of the central nervous system (CNS), phenotypic differences between children and adults, the predominance of malignant cells in CNS tumors, and the potential benefits of immunotherapy in treating pediatric brain tumors, it remains unknown whether a distinct immune-stromal-tumor ecosystem exists.

In this study, combining with the published and newly generated scRNA-seq data, we mapped the landscapes of the TME across six major brain tumor types and comprehensively analyzed the immunophenotypes of T cells and myeloid cells in different cancer types. Different from the definitions of myeloid cells using a single gene in previous studies, we combined the marker genes with mostly affected pathways to discover the recurrent function phenotypes across different brain tumors. In addition, considering the unique stromal composition and predominance of malignant cells in pediatric brain tumors, we constructed the gene features of different cell types (namely, scFes) including the tumor-related features based on single-cell analysis and finally identified 12 immune-stromal-tumor patterns. We believe this study will provide a comprehensive compendium to understand the complexity of the TME and potential strategies for the upcoming tide of immunotherapy in pediatric brain tumors.

## 2 Materials and methods

### 2.1 Single-cell RNA-seq datasets collected in this study

**Figure 1:**
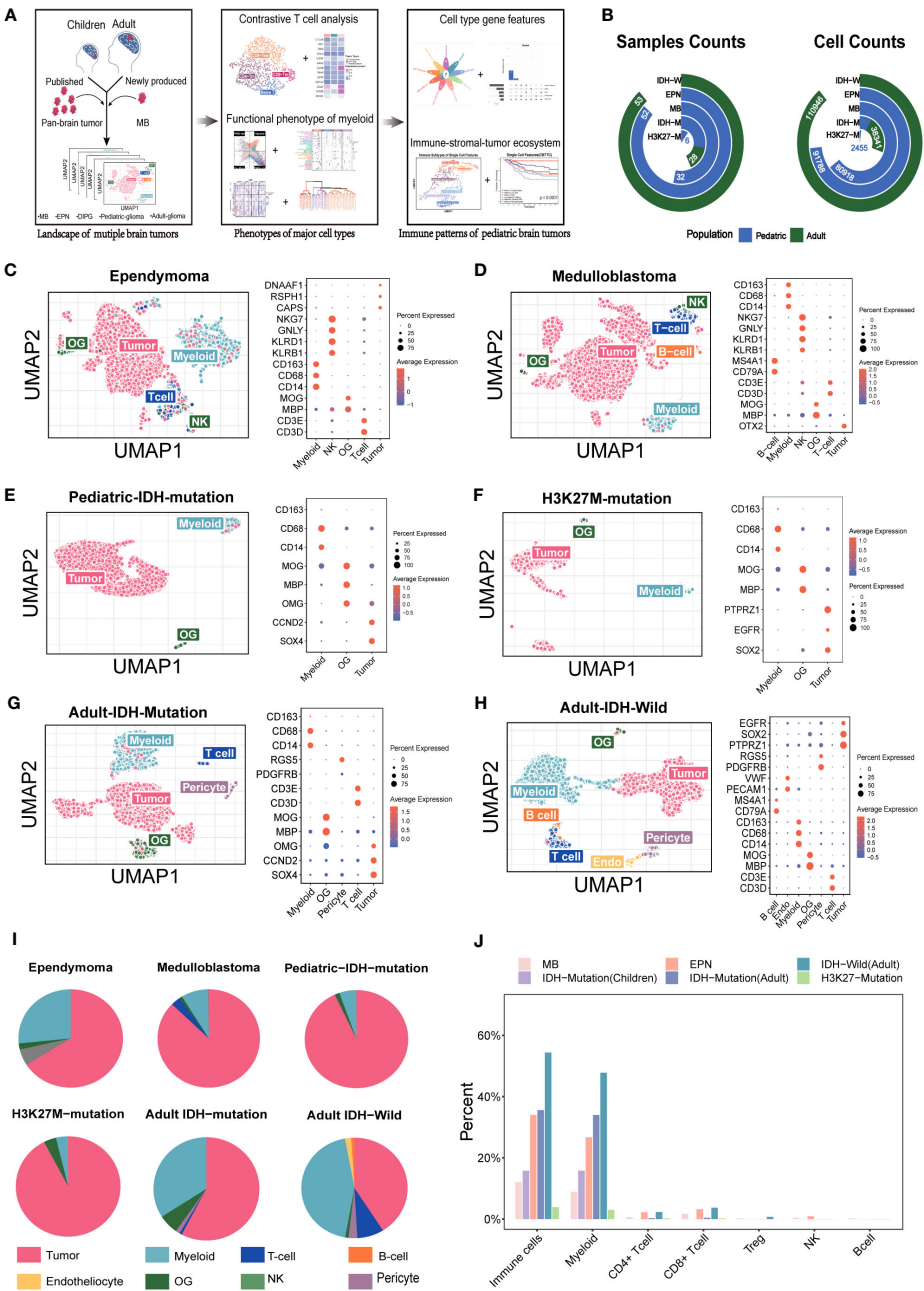
Overview of the single cells from the major pediatric brain tumors and adult gliomas. **(A)** Summary of the workflow used to analyze the immune components and functional phenotypes of myeloid and T cells, construct gene features, and identify the immune. **(B)** The included sample numbers and cell counts. **(C–H)** (Ependymoma, medulloblastoma, pediatric IDH-mutation glioma, H3K27M-mutation glioma, adult IDH-wild glioma, adult IDH-mutation glioma) Uniform Manifold Approximation and Projection (UMAP) plot of the analyzed single cells and dot plot of marker genes for each cell type. Each color represents one cell type. **(I)** Pie chart showing the relative size of each cell type. **(J)** The comparison of immune components among the major pediatric brain tumors and adult gliomas.

We collected published scRNA-seq data covering four pediatric brain tumor types (ependymoma (EPN), medulloblastoma (MB), IDH-mutation glioma (P-IDH-M), and H3K27M-mutation glioma (DIPG)) and two adult gliomas [IDH-wild glioma (adult-IDH-W) and IDH-mutation glioma (adult-IDH-M)] (**Figure 1A**).

To supplement the publicly available data and study the immune components in the “cold” tumors (16, 17), we collected five specimens of MB (including three unpublished datasets generated previously and two newly produced) (18) and obtained the snRNA-seq data using the 10x Genomics platform (19) (**Table S1**). The study protocol was approved by the Ethics Committee of Xinhua Hospital Affiliated to Shanghai Jiao Tong University School of Medicine, and the written informed consents were obtained from all patients.

### 2.2 Primary scRNA-seq data preprocessing

#### 2.2.1 Data qualification and transformation

For the newly generated snRNA-seq data from 10x Genomics, the Cell Ranger (version 3.0, 10x Genomics Inc.) was used for the alignment and quantification of sequencing reads against the GRCh38 human reference genome. The cells with fewer than 2,000 UMI counts, less than 200 detected genes and >10% mitochondrial gene count, were filtered out. DoubletFinder with default parameters was applied to remove the potential doublets (20).

For previously published scRNA-Seq data, the quality-passed cells from the original publications were used for downstream analysis. Count data generated based on 10x Genomics Chromium were normalized by the NormalizeData function from the Seurat packages (version 4.1.1) (21). The TPM data generated based on Smart-seq2 were log2-transformed.

#### 2.2.2 Comparing two methods identifying known cell types

The Cluster-based method was based on the Seurat pipeline, and two or more marker genes were used to annotate the cell types (for example, MBP, MOG, and PLP1 were combinedly to mark the mature oligodendrocytes). Another method named “positive selection” (cells with positive expression of known markers) were performed according to the expression of single marker gene (for example, oligodendrocytes were annotated if the expression level of “MBP” was higher than the average level). Then, the cell numbers, mean expression level of markers, and mean enrichment scores of mark pathway were compared.

#### 2.2.3 Clustering per dataset

Two-run clustering was performed on every dataset to reduce the technical noise. The first-run clustering was to obtain the coarse cell types. The percentages of mitochondrial genes and heat shock protein genes were calculated and added using the AddMetaData function. The cell-cycle score of each cell was scored by the CellCycleScoring function for the G2/M and S cell-cycle phases. The 10x Genomics-based dataset was renormalized by the SCTransform function, and the donor effect, number of UMIs, percentage of mitochondrial transcripts, percentage of heat shock protein genes, and cell-cycle scores were regressed out. The top 2000 genes were identified as highly variable genes (HVG) and used for principal component analysis (PCA). The Shared Nearest Neighbor (SNN) graph was built with the top 15 principal components, and the cells were clustered using the Louvain algorithm with default parameters. The primary cell types were annotated according to the marker genes. Then, the second-run clustering was performed on each cell type as the pipeline of first-run clustering. The resolution parameter of clustering was set to 50 to construct the mini-clusters to find out and exclude the contaminant cells or doublets. The reminding cells were kept for the downstream analysis.

### 2.3 Ro/e analysis for the tissue abundance of T-cell subpopulations

To characterize the tissue distribution of a specific T subpopulation, odds ratios (ORs) were calculated and used to indicate preferences. We constructed a 2 × 2 contingency table. This table included the number of cells belonging to the target T-cell subpopulation i in tissue j, the number of cells of T cell i in other tissues, the number of cells of non-i T cells in tissue j, and the number of cells of non-i T cells in other tissues. To determine the significance, Fisher’s exact test was applied to this contingency table, allowing us to obtain the OR and corresponding p-value. The p-values were then adjusted using the BH method implemented in the R function p.adjust. Consequently, a higher OR with a value above 1.5 indicated a preference for the target T-cell subpopulation i to distribute in tissue j. Conversely, a lower OR with a value below 0.5 indicated a preference for T cell i not to distribute in tissue j.

### 2.4 Conduction of two independent RNA-seq cohorts of pediatric brain tumors

#### 2.4.1 RNA-seq cohorts of pediatric brain tumors from the Children’s Brain Tumor Tissue Consortium

Transcriptomic data and clinical data were downloaded from the USCS XENA portal https://xena.ucsc.edu/as FPKM units. Overall, 11 pediatric brain tumor types, namely, anaplastic astrocytoma (AA), astrocytoma (AS), atypical teratoid rhabdoid tumor (ATRT), choroid plexus papilloma (CPP), craniopharyngioma (CPG), diffuse intrinsic pontine glioma (DIPG), ependymoma (EPN), ganglioglioma (GG), medulloblastoma (MB), oligodendroglioma (OG), and primitive neuroectodermal tumor (PNET), were used in this study. In total, 679 pediatric samples were included for further analysis.

#### 2.4.2 Independent external cohort of pediatric brain tumors from GEO

We collected 44 public datasets using Affymetrix protocol (U133 Plus 2.0 Array) from the GEO (total samples number = 2,331). The adult patients were excluded. CEL files were processed using the gcRMA package and log2 transformation for consistent normalization. The criteria of quality control were set as low correlation (<0.8) and similarity (outlier distribution *via* k-means analysis) with each tumor type. Finally, out of 2,331 samples, 1,245 were retained for further analysis. The batch effect was removed with the preservation of tumor characters using the combat function of the sva package.

### 2.5 Purified cell type compendium including immune, stromal, and tumor cell lines

We collected 76 RNA-seq gene expression datasets (1911 samples) based on the GPL570 platform from GEO to create a cell compendium, including sorted T cells, B cells, NK cells, granulocytes, endotheliocytes, oligodendrocytes, pericytes, medulloblastoma cells, glioma cells, and ependymoma cells. CEL files were downloaded and processed using the gcRMA package and log2 transformation for consistent normalization.

### 2.6 Discrimination of MG and BMDM in 10× datasets based on machine-learning method

Due to the low expression rates of classical MG and BMDM in 10× datasets, a pipeline based on random forest was constructed. Previous studies reported that MG and BMDM in the gliomas and brain metastases always increased the other core gene set signals but still maintained their cell type specificity. Therefore, we hypothesized that some stable core genes maintaining the specificity of MG and BMDM might exist across different brain tumors and could be detected by different platforms. Then, we constructed a machine-learning pipeline based on random forest to discriminate the MG and MBDM in MB (**Figure 3A**). For EPN, the IDH wild glioma which had paired 10× and Smart-seq2 data, the Smart-seq2 datasets were used to identify the MG and BMDM through the Seurat pipeline with the classical markers. First, the cells of IDH wild glioma and ependymoma from Smart-seq2 were clustered *via* the Seurat pipeline. The resolution parameters were set at 0.3 and were defined as MGs and BMDMs according to their respective markers (**Figures S3A-C**). Then, the classifications based on the random forest of IDH wild and ependymoma were conducted, and their ability of discriminating the MG and BMDM in the smart-seq datasets were validated (**Figures S3B, C**). Then, the classifications were applied to the pared 10× data of EPN and IDH-wild gliomas. After that, the classifications based on the annotated Smart-seq2 datasets were constructed using the randomForest function from the randomForest package with default parameters. The classifications were evaluated with the cmdscale functions. Then, the classifications were used to predict the cell types in the 10x Genomics datasets of corresponding tumor types (EPN the IDH wild glioma).

The differential expression genes (DEGs) between MG and BMDM of EPN the IDH wild glioma were analyzed respectively with the FindMarker function (logFC >0.25, adjust p value <0.01). Moreover, the intersection of DEGs was regarded as the conserved DEGs of MG and BMDM fit on the 10x Genomics platform. The Seurat object of MB and IDH-mutation glioma based on the 10x Genomics platform, only containing the conserved DEGs, was conducted and analyzed with the Seurat pipeline. All the conserved DEGs were used as HVGs when the PCA was performed. The resolution parameters were set at 0.2.

### 2.7 Identification of immunophenotypes of myeloid cells in different tumor types

#### 2.7.1 Clustering myeloid cells

To reduce the noises from different platforms and studies, a five-step procedure was applied (22). First, after extracting myeloid and clustering with default parameters, respectively, the aov package was used to perform the analysis of variance (ANOVA) and obtain the F values of every gene. The percentile ranks of F values was calculated. Second, genes were ordered ascendingly by the median of percentile ranks across different datasets. Third, excluding the ribosome genes, cell-cycle genes, and heat shock protein genes, the top 2000 genes detected in over half of datasets were identified as informative genes. Fourth, in order to comprehensively analyze the myeloid subsets of the major brain tumors, we integrated two independent datasets by Seurat for each tumor type (MB, EPN, P-DH-M glioma, and adult IDH mutation glioma) to obtain a larger cell number (16, 17) (**Figures S4A-E**). Due to the large samples of adult IDH wild gliomas a recent study provided, we just selected the primary samples for the analysis of the myeloid subset (23). We integrated the myeloid compartments by the PrepSCTIntegration function from the Seurat package to obtain a large cell number in these “cold” tumors. The parameters of k.weigh and k.filter were set according to the cell counts. Fifth, the informative genes were used as HVGs when the PCA was performed and the resolution parameter was set from 0.1 to 2 to obtain the different cluster numbers. The Davies–Bouldin index (DBI) was to determine the best number of clusters.

#### 2.7.2 Functional annotation of myeloid cells with marker genes and pathways

For the purpose of functional comparison of myeloid subsets among different brain tumors, we use the marker genes and most affected pathways to define their functional phenotypes. Marker genes combined with most affected pathways were used to comprehensively annotate the myeloid cells from different tumor types. First, the FindAllMarkers function from the Seurat package was used to find out the marker genes, and the gene ontology (GO) analysis was performed with the clusterProfile package (version: 4.2.2) based on marker genes to determine the affected pathways (24). In addition, the gsva function from the GSVA package (Version:1.42.0) was performed on the expression data to obtain the matrix of cells and pathways (25). Then, the differential pathways of each clusters were analyzed with the limma package (3.50.3) (logFC >0, adjust p value <0.05) (26). Then, the intersected pathways of GO analysis and differential pathways from GSVA were ranked according to the enrichment score and the marker genes in the top intersected pathway were ranked by logFC. The intersected pathway with maximal enrichment score and containing top10 marker genes was determined as the marker pathway. The corresponding marker genes with the maximal logFC were used as the marker genes. A bubble plot was made to compare the median −log10 of q value and median gene counts of specific pathway in different clusters. A heat map was to compare the mean of z-score-transformed expression value of the marker genes determined in this study and those previously reported.

### 2.8 Similarity analysis of clusters from different tumor types

The integrated gene expression matrices were z-score-transformed averaged per cluster. Thus, the original gene by cell expression matrix was converted to the gene by cluster expression matrix. Matrices of EPN, MB, pediatric IDH mutation, adult IDH wild, and adult IDH mutation were combined by column, and only genes present in all datasets were retained. The combined matrix was used for hierarchical clustering with the hclust function, and the dendrograms were created by as.ggdend from the dendextend package (27).

### 2.9 Construction of gene features (scFes) describing TME properties

In order to obtain the gene features of classical cell types that could be applied to different tumor types and different platforms, a five-step procedure was applied. First, the common adult and pediatric tumor types including adult IDH-W and adult IDH-M gliomas, EPN, MB, DIPG, and P-IDH-M were incorporated in, and most tumor types contained two types of datasets based on 10x Genomics and Smart-seq2 platforms. Second, the gene features of specific cell type were identified per dataset using the FindMarkers function. The threshold values were determined according to the cell types. The rigorous parameters were chosen for the common cell types, such as myeloid cells, T cells, CD4, CD8, B cells, NK cells, oligodendroglia cells, endothelial cells, and pericytes (adjust p value <10e-20, min.pct >0.3, and logFC >1 for Smart-seq2; adjust p value <10e-10, min.pct>0.1, and logFC >0.25 for 10x Genomics). The relatively loose threshold values were chosen for the subpopulations of major cell types, such as MG, BMDM, naïve T cells, CD4 memory cells, CD8 memory cells, and CD8 effector cells (adjust p value <10e-10, min.pct >0.3, and logFC>1 for Smart-seq2; adjust p value <10e-10, min.pct >0.1, and logFC >0.25 for 10x Genomics). Third, the intersected gene features of every cell type from each dataset were obtained. The gene features of cytotoxic CD8 T cells were chosen from the genes existed in three quarters of datasets containing this cell type. Due to the heterogeneity of tumor components in different tumor types, genes meeting the threshold values of common cell types in more than one tumor types were selected as the conserved tumor features.

### 2.10 Construction of gene features of immune-stromal patterns

In order to extend the immune-stromal-tumor patterns in the external dataset (GEO), the gene signatures were generated by DEG analysis between the specific archetype and each of the other 11 archetypes, using limma and Voom (p value <0.05, logFC >1)(15). The intersection between the top 3,000 genes by logFC of each of 11 DEGs per archetype was assigned as an initial gene features. If the initial gene features had more than 20 genes, coefficients of variation (CV) were calculated and the top 20 genes with the lowest CV and detected in at least 80% of cells of corresponding archetype were defined as the archetype gene features. If the initial gene features were less than 20 genes, the initial gene features were defined as archetype gene features.

### 2.11 Multiplex immunohistochemistry

For multiplex immunohistochemistry (mIHC) staining (CD11b, HLA-DQA1, and CD1E for cluster 2 in medulloblastoma, and CD11b, CD3, and MAG for immune patterns), co-staining of the selected markers was performed using a Four-Color Fluorescence Kit (Recordbio Biological Technology, Shanghai, China) based on the tyramide signal amplification (TSA) technology according to the manufacturer’s instruction. All the slides were scanned using a Pannoramic P-MIDI (3DHISTECH, Hungary). The positive cell numbers were calculated by HALO 3.3 software (Indica Labs, USA).

## 3 Results

### 3.1 Low immune infiltration of pediatric brain tumors

To dissect the tumor ecosystems in children, in addition to the accessible public data, we collected five medulloblastoma specimens from four patients for snRNA-seq based on 10x Genomics. The predominant functional phenotypes of T cell and myeloid subsets and immune-stomal-tumor patterns in children were investigated (**Figure 1A**). In total, more than 0.3 million cells from 171 samples covering adult gliomas and four major pediatric brain tumor types were included (**Figure 1B**).

To analyze the TME components of each tumor type, we evaluated the major current methods identifying known cell types, namely, clustering-based (CB) method and positive expression of marker genes (PEMG) method. We found that the CB method performed better overall (**Figure S1**). For the datasets based on 10x Genomics or Smart-Seq2, the CB method was able to identify more cells than the PEMG method in most tumor types, which was important for the “cold” tumor types. Our data indicated that the “cold” tumor types like MB and EPN also contained the major classical immune cells, such as T cells (PTPRC, CD3D), B cells (CD79A, IGHG1, MZB1), and NK cells (KLRB1) (**Figures 1C–H**). In addition, the immune components varied significantly among the different brain tumors (**Figure 1I**) and were lower in the pediatric brain tumors (MB, EPN, and pediatric IDH-M) than those in the adult brain tumors (IDH-W and adult IDH-M) (**Figure 1J**). Similar with the previous studies, myeloid cells were predominant among all the immune cells in brain tumors, and MB and EPN held a relatively higher rate of CD8 T cells among all the tumor types (6). However, the functional phenotypes of them in pediatric brain tumors are still unknown.

### 3.2 Targeting the T cells may not be the optimal strategy for MB and EPN

**Figure 2:**
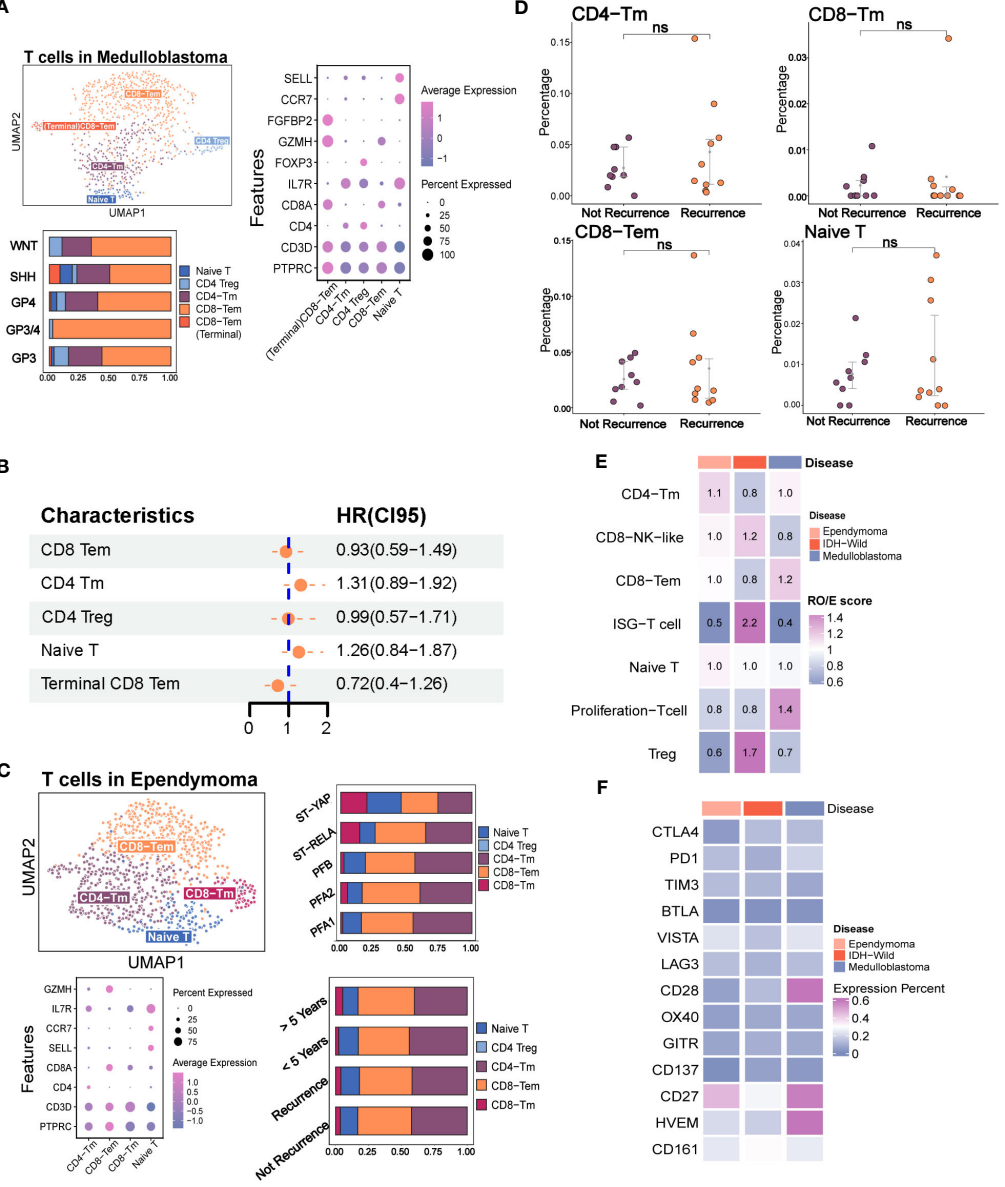
The immune phenotypes of T cells in the major pediatric brain tumors. **(A)** The evaluation of T-cell subpopulation in medulloblastoma including the UMAP plot of the identified T-cell subsets, dot plot of marker genes for each cell type, histogram for the relative size of each subset among different molecular subtypes, and Kaplan–Meier plot for terminal CD8 effector memory T cells. **(B)** Forest plot shows none of the T-cell subpopulations are independent factors of prognosis. **(C)** Evaluation of the T-cell subpopulation in ependymoma including the UMAP plot of the analyzed single cells, dot plot of marker genes for each cell type and histogram for the relative size of each subset among different molecular subtypes and prognosis groups. **(D)** Point plot shows that none of the T-cell subpopulations have significant changes between recurrence and non-recurrence groups. **(E)** Heatmap reveals the tissue prevalence of each T-cell subpopulation by Ro/e score. **(F)** Heatmap shows the expression percentage of immune checkpoint molecules in T cells of ependymoma, medulloblastoma, and adult IDH wild gliomas. The "ns" represents "not significant".

According to the previous studies on various types of tumors, certain subsets of T cells, including effector T cells, memory T cells, and exhausted T cells, have been found to have specific functions in either eliminating or tolerating tumor cells within the tumor microenvironments (22, 28, 29). Due to the extremely low contents of T cells in IDH mutation glioma and H3K27 mutation glioma, we focused on the characteristics of T cells in the MB, EPN, and adult IDH wild glioma (**Figures 2A, B**, **S2D**). In order to explore the phenotypes of T cells in MB and EPN, we defined the subpopulations of T cells according to the known markers identified in a recent study of T-cell atlas. The results revealed that T cells in MB mainly consisted of CD8 effector memory T cells (CD8 Tem, GZMH), CD8 terminal effector memory T cells (terminal CD8 Tem, FGFBP2), CD4 memory T cells (CD4 Tm, IL7R), regulatory T cells (Treg, FOXP3), and naive T cells (Tn, CCR7, and SELL) (**Figures 2A**, **S1A**) (22). Different molecular subtypes of MB held a heterogeneous composition of T-cell subpopulations (**Figure 2A**). Moreover, CD8 Tem formed the majority of T cells in all of the subtypes (**Figure 2A**). We also found that the higher scores of CD8 Tem markers were associated with a survival advantage in MB, whereas an opposite effect was observed in CD4 Tm and Tn (**Figure S2B**). However, the univariate Cox analysis revealed that the T-cell subpopulations did not significantly correlate with prognosis (**Figure 2B**). Considering that Kaplan–Meier (KM) analysis is a non-parametric method and Cox regression is a semiparametric method that takes multiple factors into account, the results of Cox analysis may indeed be considered to have higher credibility (30). In EPN, T cells mainly consisted of CD8 Tem, CD8 Tm, CD4 Tm, and Tn. The subpopulations of T cells were evenly distributed in different molecular subtypes (**Figures 2C**, **S2C**). Moreover, both the group with a follow-up period of over and less than 5 years, as well as the recurrence and non-recurrence groups, exhibited similar percentages of T-cell subtypes (**Figure 2C**). There was also no significant difference in the percentages of four subpopulations between the recurrence and not recurrence groups (**Figure 2D**).

We next quantified the tissue enrichment of T-cell subsets among the different tumor types by integrating the different T-cell data from different datasets (**Figures S2E-G**). The IFIT3+ T cells and Treg identified within the three tumor types were preferentially enriched in adult IDH-W gliomas (**Figure S2H**). The Ro/e analysis also demonstrated the preferences (**Figure 2E**). Based on the comparison of expression percentage of immune checkpoints, we observed that classical molecules like PD1, CTLA4, TIM3, and LAG3 and newly reported molecules like CD161 were extremely low in these brain tumors, which might partly explain the difficulty for their clinical transformation in brain tumors (**Figure 2F**) (31). Overall, the low contents of T cells and low expression rates of immune checkpoints indicated that the traditional immunotherapy strategies targeting the local T cells in the tumor microenvironment, such as immune checkpoint blockade, may not be the optimal treatment strategy for pediatric MB and EPN.

### 3.3 Assessment of known functional phenotypes of myeloid cells

**Figure 3:**
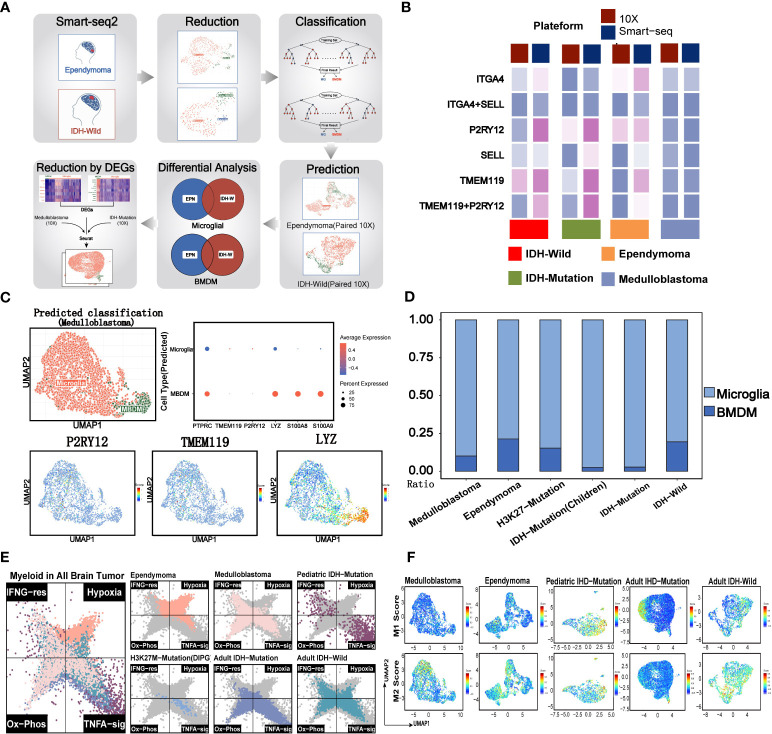
Evaluation of traditional immune phenotypes of myeloid cells in pediatric and adult brain tumors. **(A)** Summary of the workflow used to discriminate the microglial and BMDM in the 10x data. **(B)** The comparison of 10x and Smart-Seq2 platforms across the different brain tumors. **(C)** Evaluation of MG and BMDM in 10x data of medulloblastoma. The UMAP plot displays the consistency between the predicted MG and BMDM and the clusters. The dot plot shows the expression of classical markers in the MG and BMDM. UMAP plots of marker genes show the expression of classical markers in the cells. **(D)** The relative size of MG and BMDM across the different brain tumors. **(E)** Two-dimensional butterfly plot visualization of classical tumor-related pathway scores in pediatric and adult brain tumors. Colors represent different tumors. **(F)** The M1 and M2 scores of the different clusters across the different brain tumors.

Tumor-associated macrophages (TAM) were regarded as a potential target in the future immunotherapy. We next accessed the characteristics of known phenotypes of myeloid cells, such as MG, BMDM, M1, and M2, in the major pediatric brain tumors. The previous studies reported that the MG and BMDM in brain tumors showed distinct transcriptomic profiles and inflammatory polarization tendency, which are additionally influenced by the underlying disease type (4). However, it was apparent from **Figure 3B** that the expression percentages of classical MG and BMDM markers (P2RY12 and TMEM119 for MG, and ITGA4 and SELL for BMDM) from the 10x platform were significantly lower when compared with those from the Smart-Seq2 technique (4, 9, 23). The results revealed that identified MGs and BMDMs were largely consistent with clusters obtained from the Seurat pipeline, indicating that the internal characteristics of MG and BMDM were basically preserved across different conditions (**Figures S3A–C**). When evaluating the classical markers in MG and BMDM, predominance in the expression levels and percentages of markers remained in the corresponding cell types (**Figures S3D, E**). Similarly, they were also highly consistent with the cluster results of Seurat (**Figures 3C**, **S3F**). The results showed that the ratio of MG and BMDM varied among brain tumors, and MG comprised the vast majority of myeloid cells (4), especially for the pediatric and adult IDH mutation gliomas (**Figure 3D**). However, the rough classification of MG and BMDM still lacks guidance for the functional phenotypes.

Previous studies reported that myeloid subsets on glioblastoma (GBM) were significantly enriched in classical inflammatory signals and metabolic pathways (4, 9, 23). The butterfly plot revealed a significant enrichment of hypoxia in myeloid cells of ependymoma, when compared with other brain tumors (**Figure 3E**). Similar with the myeloid cells of adult brain tumors, myeloid cells of medulloblastoma were significantly enriched in oxidative phosphorylation and TNFα pathways while deficient in hypoxia signals. Classical inflammatory hallmarks (IFNα response and TNFα-signaling) were enriched in myeloid cells of pediatric IDH mutation gliomas, indicating the anti-tumorigenic phenotypes of these cells in the microenvironment.

Then, we investigated the M1 and M2 signature scores in the clusters of all the tumor types (**Figure S3G**). We found the co-expression of both M1 and M2 gene signatures in most of myeloid subsets from EPN, pediatric IDH-M glioma, and IDH-W glioma. Additionally, certain clusters exhibited both lower M1 and M2 gene feature scores, suggesting that the categorization of M1 and M2 may not be entirely applicable for the classification of myeloid brain tumors (**Figure 3F**) (23, 32). Therefore, this defective classification of myeloid cells in brain tumors suggested that it was significant to further uncover the function in a specific tumor microenvironment.

### 3.4 Comparative analysis of functional states of myeloid subsets from pediatric and adult brain tumors

**Figure 4:**
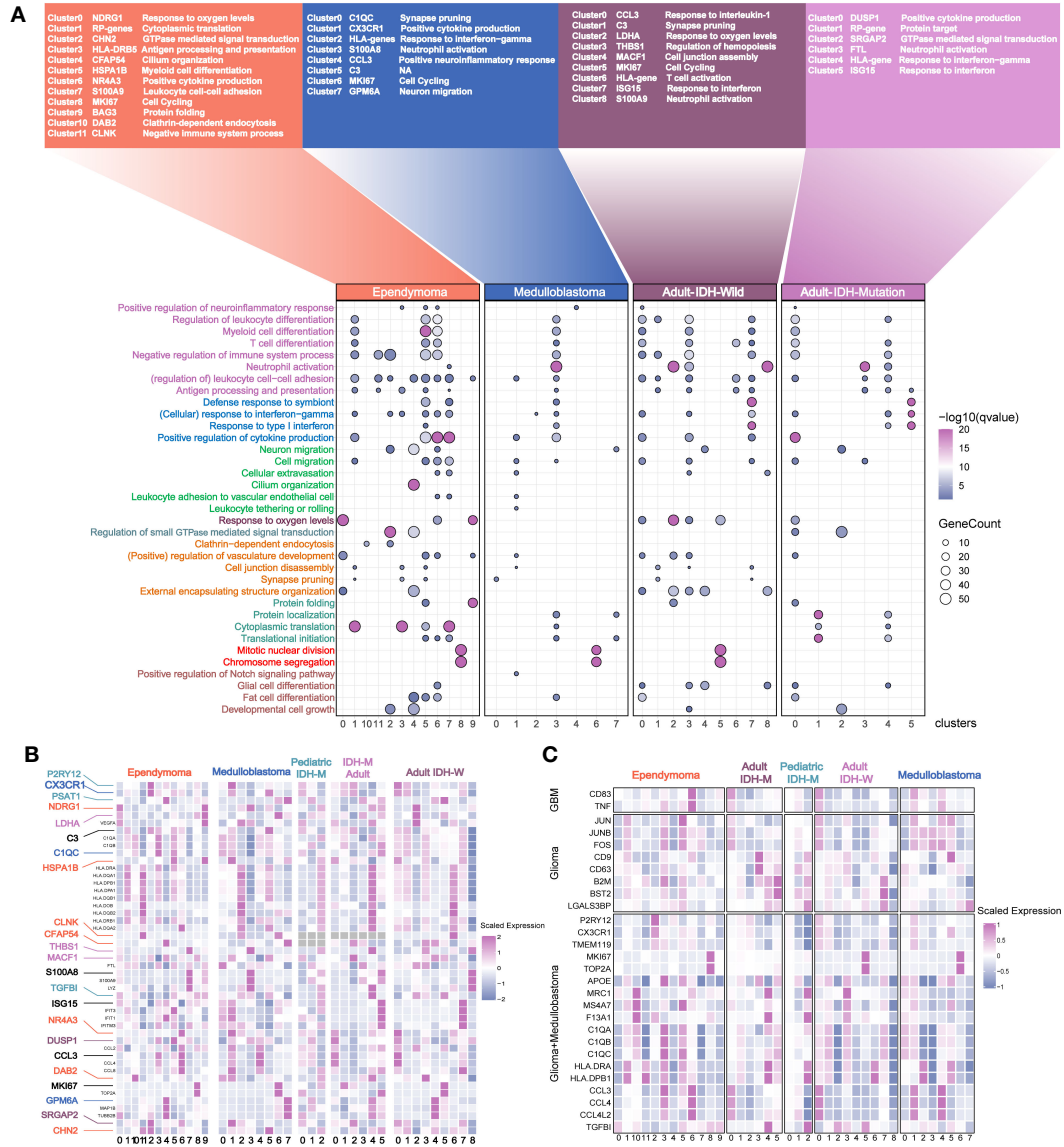
Annotation of the myeloid subsets from the major pediatric and adult brain tumors. **(A)** The names of the different clusters. The most affected terms in each cluster are represented in a dot plot, with the size of the dot corresponding to the number of genes per term and the color of the dots corresponding to the q value of enrichment after –log10 transformation. **(B)** The selected genes with different colors are used as the marker genes. Color-coding is consistent with the tumor types. **(C)** The previously reported marker genes of myeloid subpopulations in the different clusters across the different brain tumors.

In order to comprehensively analyze the myeloid subsets of the major brain tumors, we integrated two independent datasets. The results revealed that the batch effects were removed (**Figures S4A–E**). The best number of clusters was evaluated by DBI (**Figure S4F**). The results showed that the myeloid cells in EPN were the most heterogeneous because of the largest cluster numbers (**Figure S4A**). Except for the pediatric IDH mutation gliomas which had no significant pathways via ssGSEA, the marker pathways of each myeloid cluster across the pediatric and adult brain tumors uncovered the common perturbation of functional modules, such as leukocyte activation and interferon response, and exclusive pathway perturbation in brain tumors, such as pathways related to cilium organization and endocytosis in ependymoma (**Figure 4A**). Then, we investigated marker genes in different clusters to find out the myeloid clusters with similar expression levels of marker genes but defined as different myeloid subsets (**Figure 4B**). For example, similar expression levels of NDRG1, LDHA, MHCII molecules, interferon genes, GPM6A, C9, and SRGAP2 were discovered in different myeloid subpopulations, indicating the similar functional states in different brain tumors. The previously reported markers were also compared among different tumor types, for example, the homeostasis myeloid subpopulation with high expression levels of P2RY12 and CX3CR1 in gliomas and cluster 2 marked with the pathway of “GTPase signal transduction” in ependymoma (9), the activated microglial subsets highly expressing CD83 and TNF in GBM, and the clusters6 marked with pathways of “positive cytokine production” in ependymoma (23) (**Figure 4C**).

**Figure 5:**
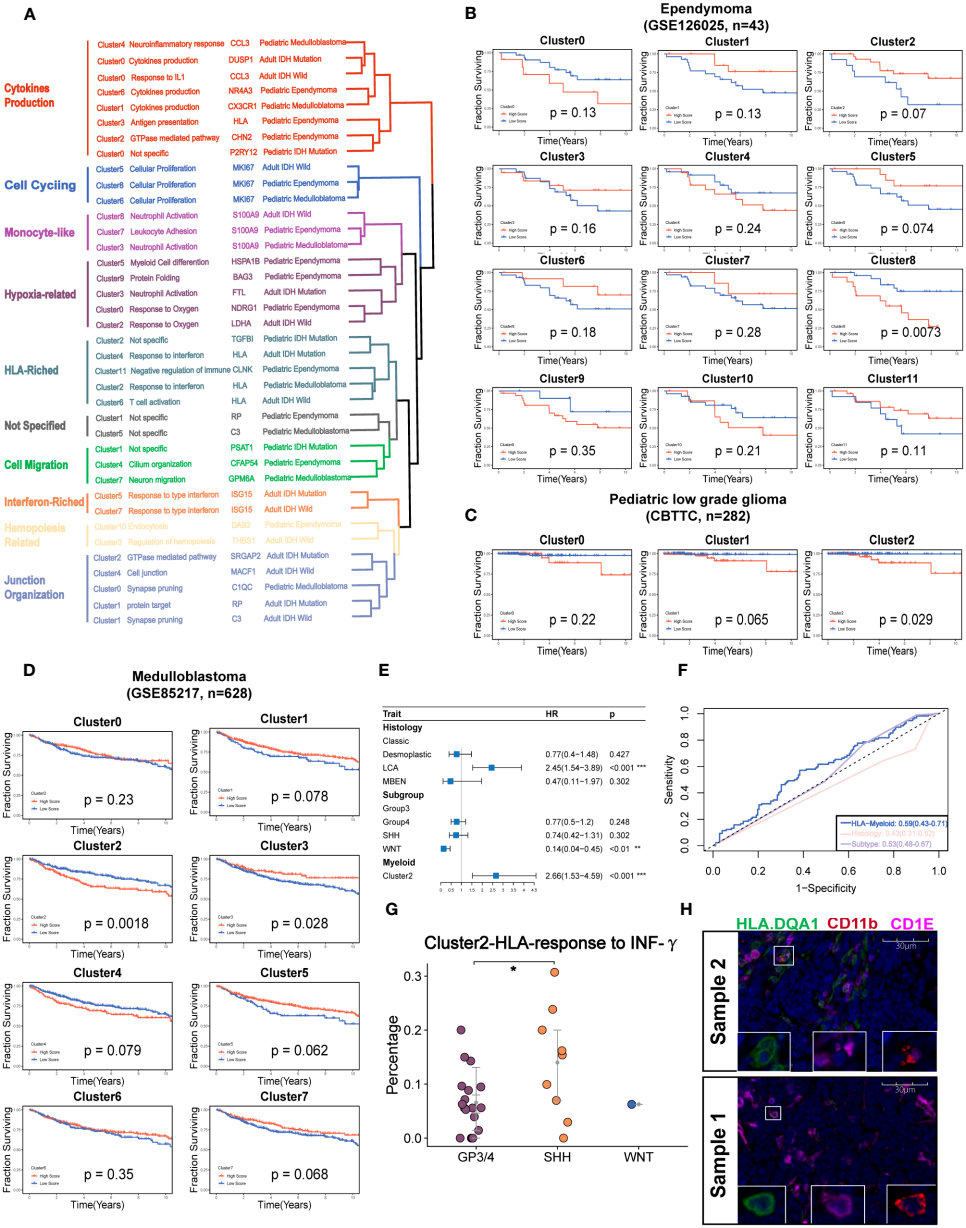
Identification of the potential targeted myeloid subsets. **(A)** Hierarchical clustering shows the similarity of clusters across the different brain tumors. **(B)** Kaplan–Meier survival curves generated with each cluster signature score of EPN using GSE126025. **(C)**. v. **(D)**. Kaplan–Meier survival curves generated with each cluster signature score of the medulloblastoma using GSE85217. **(E)** The forest plot reveals that the cluster 2 of medulloblastoma is the independent factor of prognosis. **(F)** The ROC curve shows a higher AUC value of cluster 2 of medulloblastoma than classical histology and molecular subtypes. **(G)** The percent of Cluster2 in medulloblastoma was significantly higher in SHH subgroup. **(H)** The mIHC demonstrates the existence of cluster 2 in the two samples of MB.The "*" represents "P value < 0.05".

To quantify their similarities, we calculated the correlations between the average transcriptome of each cluster in different tumor types. As expected, the same major lineages from different cancer types, such as cycling, monocyte-like, hypoxia-related, cytokine-stimulated, and interferon-related subpopulations, were clustered together, further demonstrating the shared myeloid lineages between pediatric and adult brain tumors (**Figures 5A**). Then, we used the angiogenic and phagocytic signatures, a dichotomous functional phenotype, to access the functional phenotypes of each cluster across the different tumor types (33). As expected, some clusters exhibited significantly preferential signature scores. However, most clusters in pediatric brain tumors had a similar score in the two phenotype signatures (**Figure S5A**). Using the public clinical data, we investigated the relationship of the different myeloid lineages with patient prognosis. The clusters highly expressing HLA genes were negatively associated with prognosis in multiple tumors except for EPN (**Figures 5B–D**, **S5C, D**). Furthermore, the HLA gene-enriched cluster in MB was the independent factor of prognosis (**Figure 5E**) and it had better performance than molecular subtypes and traditional histology when predicting the 5-year survival (**Figure 5F**). Then, we determined three markers to define this cluster (**Figure S5B**). By conducting subtyping analysis on medulloblastoma, it was found that this cluster exhibited significant subtyping preferences, primarily existing in the G3 and SHH subtypes (**Figure 5G**). The mIHC staining of tumor sections further confirmed the existence of this subset (**Figure 5H**).

### 3.5 Establishment of TME gene expression signatures

**Figure 6:**
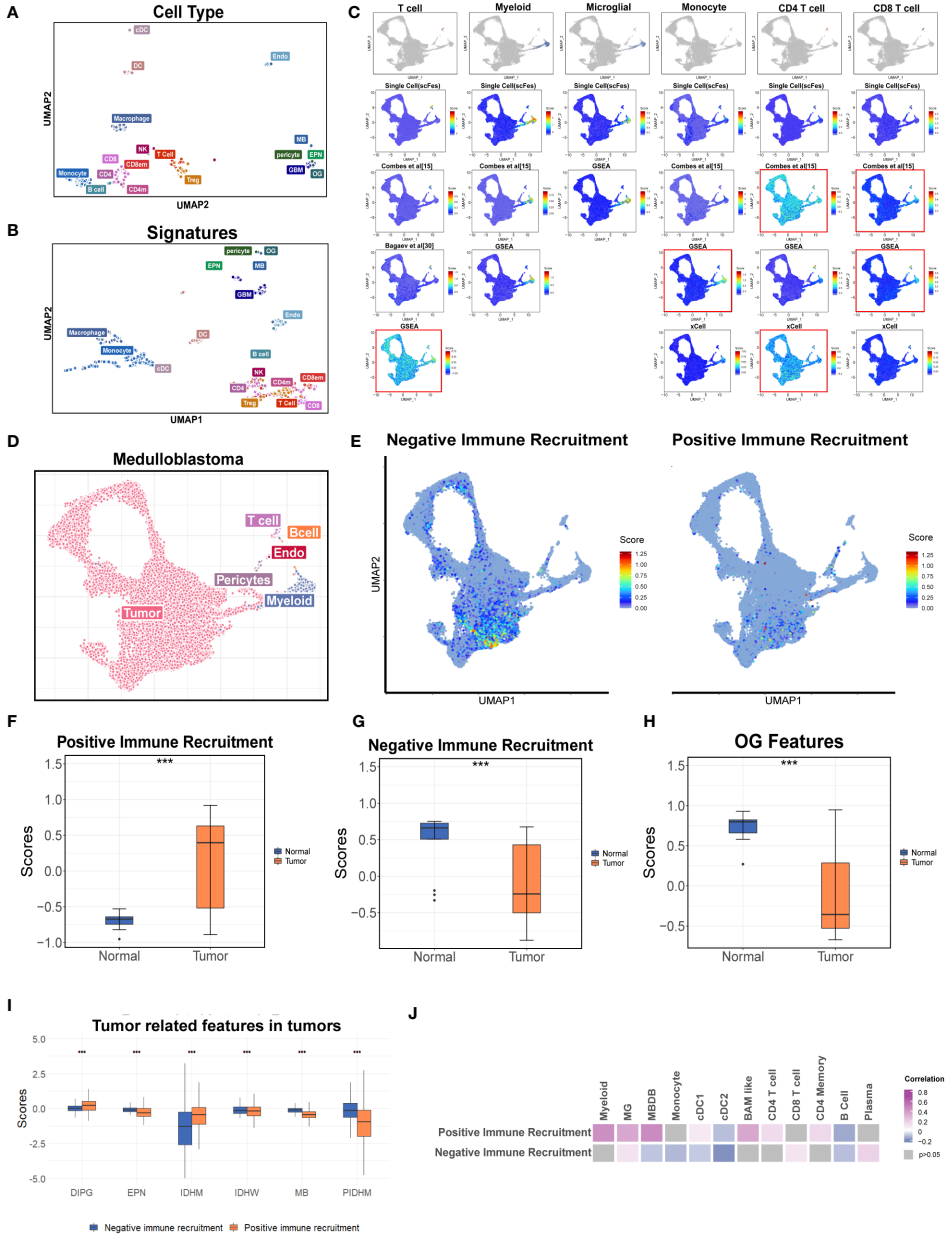
Construction the gene features of different cell types in the TME. **(A)** UMAP of the purified cell samples marked with the original cell annotation. **(B)** UMAP of purified cell samples in the space of the scFes scores. **(C)** UMAP overlays of the feature scores of major cell types in the single-cell dataset of MB (newly produced). **(D)** UMAP plot of the newly produced single cell data. Each color represents one cell type. **(E)** UMAP overlays of the tumor-related features. **(F)** Box plot shows the signature score-positive immune recruitment in GSE50161 including the medulloblastoma, ependymoma, glioblastoma multiforme, pilocytic astrocytoma, and normal brain. **(G)** Box plot shows the signature score-negative immune recruitment in GSE50161 including the medulloblastoma, ependymoma, glioblastoma multiforme, pilocytic astrocytoma, and normal brain **(H)** Box plot shows the OG signature score in GSE50161 including the medulloblastoma, ependymoma, glioblastoma multiforme, pilocytic astrocytoma, and normal brain. **(I)** Box plot shows the comparison of tumor-related signature scores across the different brain tumors. **(J)** Heatmap reveals the correlations between the tumor-related signature score and scFeg scores of different immune cell types. "***" represents the "P value <0.001".

To analyze TMEs using the transcriptomic data, the gene expression signatures (scFes) of immune and stromal components were constructed via combining multiple datasets of different tumor types from 10x and Smartseq2 platforms to find out the conserved gene signatures (Methods, **Figure S6A**, **Table S2**). We compared the scFes with the previously reported gene sets and found only small overlap among them (**Figures S6B–I**). To confirm the cell type-specific expression patterns of scFes, 1,891 RNA-seq profiles of sorted cell subpopulations across multiple GEO datasets were conducted and the final scFes were highly cell type specific and showed effective segregation, with high expression scores for cell types associated with each signature (**Figures 6A, B**). In addition, we evaluated scFes in the averaged expression data of cell line (34) and found that scFes performed better when marking CD8 T cells and Treg (**Figures S6J, K**). Furthermore, we conducted validation and comparison of scFes with published counterparts in independent scRNA datasets of various tumor types at the single-cell level. Our analysis revealed that scFes effectively identified cell types annotated by classical markers and outperformed gene features of certain cell types from GSEA (35), xCell (36), and recently published studies (15) (**Figures 6C, D**, **S7A, B**).

Considering the important roles of tumor cells and the unique role of OG in the cell networks of the brain TME depicted by the single-cell datasets, the gene signatures of tumor components correlated with the recruitment of immune cells (named as “positive immune recruitment” (positive-IR) and “negative immune recruitment” (negative-IR) which were positively and negatively correlated with the expression level of CD45, respectively) and OG were also explored to create a holistic approach describing the TME of brain tumors. We accessed the two tumor features in a newly produced dataset of medulloblastoma, which was a kind of well-known “cold” tumor, and found that they were predominantly existed in the specific tumor clusters, and the negative-IR score was significantly higher, indicating the tumor components might contribute to the deficiency of immune components in medulloblastoma (**Figures 6D, E**, **S8A**).

Furthermore, we accessed the two tumor features and OG features in two independent bulk RNA datasets of pan-cancer (**Figures 6F–H**). The results revealed that the positive-IR score was significantly higher in the tumor group and might be associated with better prognosis (**Figures 6F**, **S8B**) whereas the negative-IR score was significantly lower in the tumor group (**Figure 6G**), according to the fact of the activated immune system in brain tumors compared with immune-privileged normal brain (**Figure 6H**). OG scores were significantly associated with prognosis (**Figure S8D**), but whether this correlation was influenced by clinical parameters such as WHO classification, metastasis state, and the history of radiotherapy and chemotherapy still required data with more comprehensive clinical information. In addition, the relatively higher positive-IR score in EPM, MB, and pediatric IDH-mutation gliomas might partially account for the lower immune content when compared with adult brain tumors (**Figures 1J**, **6I**). Moreover, the paradox between the higher positive-IR and lowest immune content in DIPG, and some exceptions of the correlations between immune recruitment feature scores and immune cell feature scores, indicated the existence of other factors affecting the immune recruitment in addition to the tumor cells (**Figures 6I–J**). The OG feature score was higher in the normal brain, suggesting that the developmental program promoting the formation of mature oligodendrocytes was blocked in tumors (37, 38). Furthermore, the higher OG feature score was associated with better prognosis (**Figure 6H**). However, the tumor-related features and this exclusive stromal component of CNS were always ignored in the current studies of the TME and deserved further study.

### 3.6 Coarse classification of immune patterns in pediatric brain tumors

**Figure 7:**
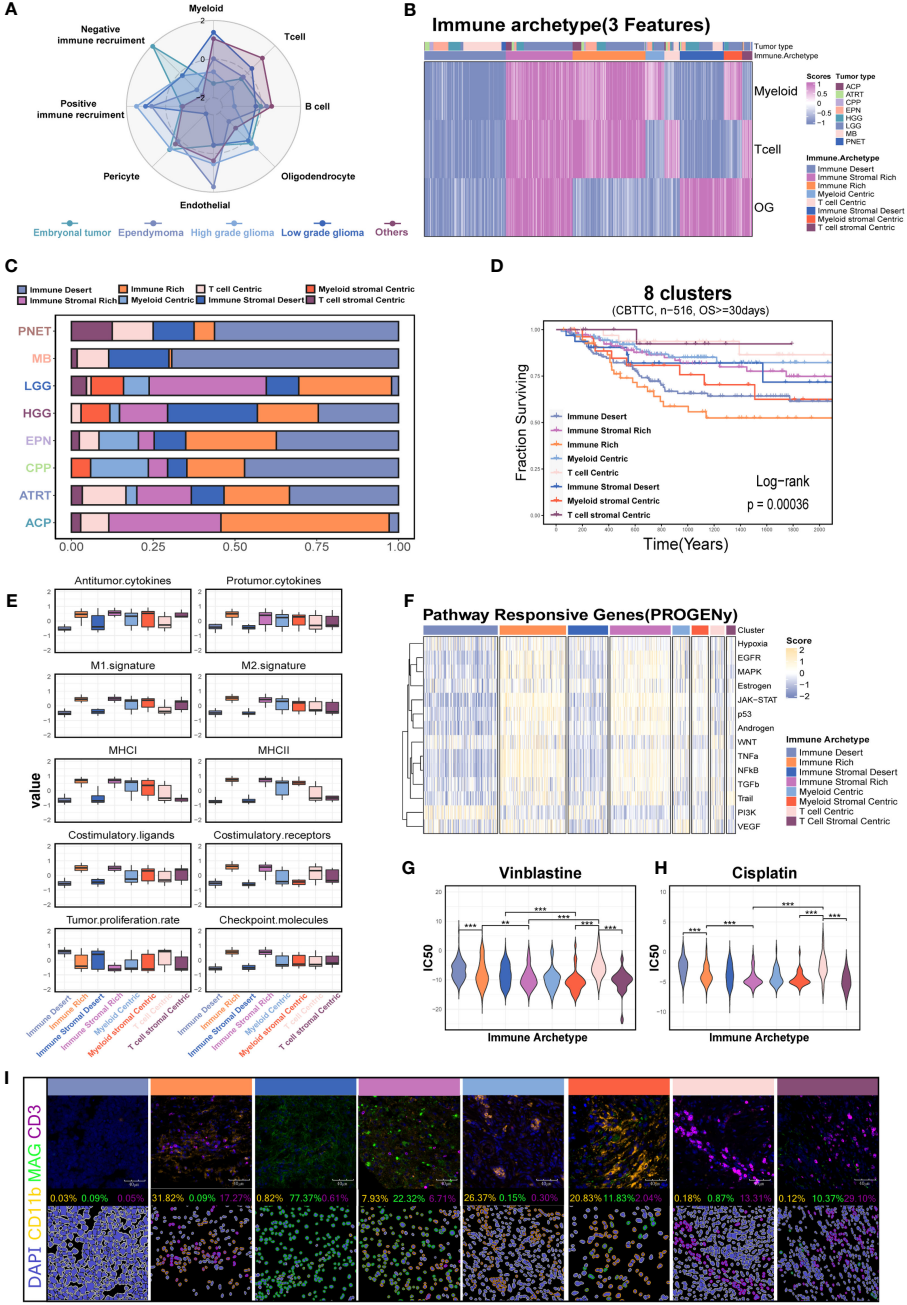
Investigation of the immune patterns based on the myeloid, T-cell, and oligodendrocyte features (3-feaure) in the CBTTC dataset. **(A)** Radar plot shows the signature score of major cell types across the major pediatric brain tumors. **(B)** The eight clusters based on the 3-feature. **(C)** Histogram for the relative size of each immune patterns among the different pediatric brain tumors. **(D)** Kaplan–Meier survival curves generated with signature scores of eight clusters (34). **(E)** Box plot shows the differences in the major immune-related processes across the eight immune patterns. **(F)** Relative signaling pathway activity scores in tumor cells measured from RNA-seq by PROGENy. **(G)** The drug sensitivity analysis of vinblastine and cisplatin among the different immune patterns. **(H)** Kaplan–Meier survival curves generated with the OG signature score using the CBTTC dataset. Box plot shows the OG signature score in GSE50161 including the medulloblastoma, ependymoma, glioblastoma multiforme, pilocytic astrocytoma, and normal brain. **(I)** mIHC demonstrated the eight immune patterns across the different brain tumors. "**" and "***" respectively represent the "P value <0.01" and "P value <0.001".

A recent study identified 12 immune archetypes across multiple cancers types but only including one pediatric brain tumor type (15). A holistic survey of the immune archetypes in pediatric brain tumors is still lacking. As expected, the cell types varied among the different tumor types (**Figure 7A**). We next explored the primary archetypes by following the same pipeline but including the unique stromal component—the OG feature. The three markers (ITGAM for myeloid, CD3 for T cell, and MAG for OG) were used for primary classification, and the DBI was used to determine the optimal cluster number. The primary classification contained eight clusters (**Figures 7B**, **S9A**, **Table S3**), including the six previously reported immune archetypes and two new small clusters (named myeloid stromal centric and T-cell stromal centric archetypes). The expression level of the three markers varied significantly among the eight clusters (**Figures S9B–D**). The immune archetypes were highly tumor specific (**Figure 7C**) and significantly associated with prognosis (**Figure 7D**).

Based on this, we next investigated the characteristics of tumor biology among the eight clusters. The immune stromal-rich and immune-rich clusters were characterized by the elevated expression scores of antitumor cytokines, M1, MHC molecules, and costimulatory molecules, demonstrating an immune-active TME compared with the immune stromal desert and immune desert clusters. However, the coexistence of the highest score of protumor factors, such as protumor cytokines, M2, and checkpoint molecules, indicated the reprogramming of the immune microenvironment in these two clusters (**Figure 7E**). The transcriptomic programs of interferon-stimulated genes (ISGs) and immune escape and chemokines also demonstrated the lasting but exhausted tumor immunology in them (**Figures S9E, F**). Conversely, the enrichment with transcriptomic programs of the cell cycle in the immune stromal desert and immune desert clusters was consistent with the increased capacity of tumor proliferation (**Figures S9F**, **7E**). In addition, the GSEA and PROGENy analysis also revealed the differential enrichment of tumor-related pathways among the eight clusters (**Figures S9G**, **7F**). The increased PI3K pathway activation in the immune desert cluster calculated with PROGENy analysis suggested the potential targeted therapy for this subset (**Figure 7F**). The differential sensitivity to the vinblastine and cisplatin, which were used as the traditional chemotherapy, might provide the possibility to the individualized treatment (**Figures 7G, H**) (39). Finally, we further confirmed the eight clusters via mIHC assays in the tumor tissues including five MBs, three EPNs, five CPGs, two CPPs, five ASs, three DIPGs, and two GGs (**Figure 7I**).

### 3.7 Immune archetypes based on 9-features

**Figure 8:**
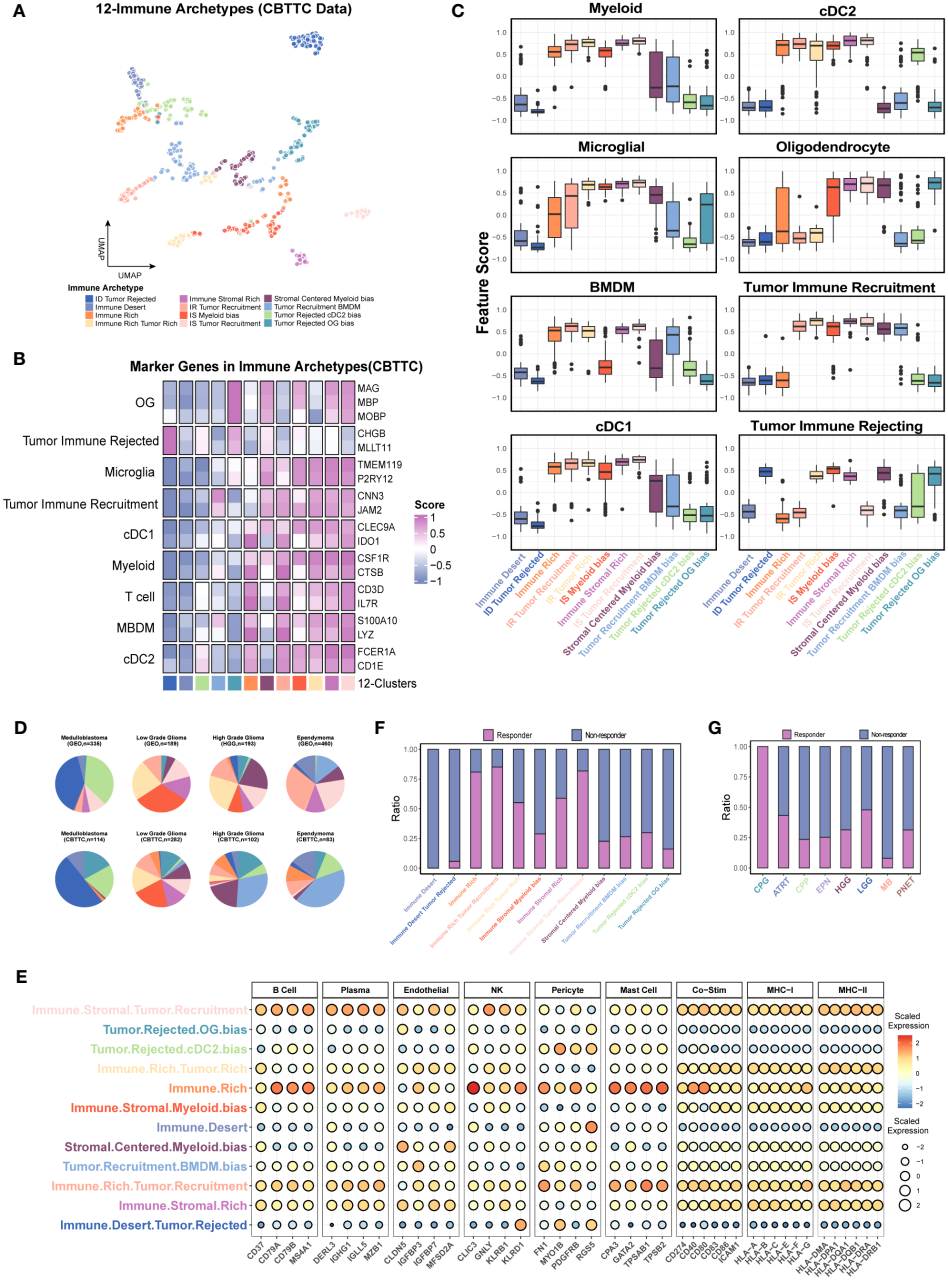
Further investigation of the immune patterns (12 clusters) based on the nine features in the CBTTC dataset. **(A)** UMAP displays the 12 immune patters in the CBTTC cohorts. Each dot represents a single cluster. **(B)** The heatmap shows the mean expression levels of the marker genes of predominant cell type in each cluster using the integrated CBTTC dataset. **(C)** Box plot shows the differences in the major cell types across the different immune patterns. **(D)** Pie charts display the relative size of immune patterns in the major tumor types. Color-coding is consistent with A. **(E)** Bubble plot reveals the signature scores of multiple cell types and immune related molecules in the 12 clusters. **(F, G)** The prediction of response rates in each cluster and tumor type.

Unlike in the non-CNS tumor types, no CD4-biased or CD8-biased tumors existed in the pediatric brain tumors when analyzed with gene sets from scFes and Combes et al. (**Figures S10A, B**). Considering the fact that coarse classification of stromal components (CD44 and CD90) used in the previous study might contain malignant cells (15) and the malignant cells might negatively or positively affect the immune recruitment, the tumor-related features (positive IR and negative IR) were also included. MG is the exclusive cellular components in the brain, and their phenotypes might be highly diverse in different pathological conditions, Therefore, the BMDM and MG features were also included for further classification. Similarly, the DBI was used to determine the optimal cluster number (**Figure S10C**). Finally, 12 immune-stromal-tumor patterns (namely, 12 clusters) were identified with nine features (**Figures 8A**, **S10D**, **Table S3**). The marker genes and gene features from scFes demonstrated predominant cell types in each cluster (**Figures 8B, C**). Furthermore, we obtained the DEGs of 12 clusters and conducted an external cohort including 1,245 children and a similar composition of tumor type with Children’s Brain Tumor Tissue Consortium (CBTTC) to validate the 12 clusters (**Table S4**). After removing the batch effect, the similar archetypes were validated in the external dataset (**Figures S10E, F**). Similar with the coarse classification based on the 9-feature, the predominant archetypes varied among the major pediatric brain tumors, and the relative composition of archetypes by tumor types was similar in CBTTC and GEO (**Figure 8D**). The inconsistency of individual archetypes between the two datasets, such as IR-tumor rich in high-grade glioma and tumor recruitment BMDM bias in ependymoma, might come from the discrepant composition of pathological or molecular subtypes. Compared with the interaction pair of some cell types in the non-CNS tumors (15), the immune component tended to synchronous change maybe due to the unique immune recruitment mechanism (**Figure 8E**). Finally, we used the TIDE score to evaluate the immune response of immune archetypes. Immune-rich and immune stromal-rich clusters had a high response rate over 50% (**Figure S10G**), whereas the further classification, namely, 12 clusters, obviously had better ability to discriminate the responsive and non-responsive subsets. Surprisingly, immune-rich, immune-rich tumor recruitment, and immune stromal tumor recruitment with a high TIDE score had a high response rate over 75% and immune desert nearly did not respond to immune checkpoint inhibitors (40). Moreover, all the craniopharyngioma (CPG) might respond to PD-1 or CTLA inhibitors (**Figure S10H**; **Figures 8F–G**). Therefore, pediatric brain tumors exhibit a distinct immune ecosystem, suggesting that CPG could potentially serve as a candidate for traditional immune therapy, such as immune checkpoint inhibitors.

## 4 Discussion

Pediatric brain tumors are the leading cause of cancer-related deaths in children, and the prognosis of certain tumor type remains abysmal, for example, the survival time of DIPG is always measured in just months. In spite of the advances in the combination of multidisciplinary diagnosis and treatment, surgery, and systemic therapy, the therapy-related long-term adverse events, such as hearing loss and neurodevelopment and neurocognitive disorders, are still the troubling complications. The extensive immunotherapy using cytokines, certain immune cells (T cell, DC, NK) and immune checkpoint inhibitors (ICIs) in the non-CNS cancers spurred the interest in pediatric brain tumors to minimize long-term morbidities (41–43). In this study, we firstly presented a holistic survey of major pediatric brain tumors from the single-cell perspective, including the immune cell abundances, identification of functional phenotypes of T cells and myeloid cells, and the immune-stromal-tumor patterns. In this study, we evaluated CB and PEMG to identify known cell types. Although the expression levels of myeloid marker gene (ITGAM) in all the tumor types were relatively lower in the CB method, the enrichment scores of the mark pathway were even higher when comparing with those of the PEMG method. The PEMG method seemed to identify more OG cells probably because of the relatively rigorous criterion of OG identification in the CB method—high expression of three marker genes (MBP, MOG, PLP1)—whereas the PEMG method just identified the cells expressing single marker genes. To construct a global tumor niche atlas of different tumor types, we performed cell clustering and marker gene identification using Seurat. In our study, we primarily utilize the marker genes employed in the original research to annotate the cells in the public datasets. The identification of malignant cells is determined by the deficient expression of established stromal marker genes or marker genes associated with mature neural or OG cells within the non-immune cell population. As we know, some tumors, like medulloblastoma, have typical copy number variations; the malignant cells were mainly inferred based on overall copy number variations in the bioinformatics analysis process. Since pediatric brain tumors generally have lower copy number variations compared with adult tumors (44), and existing copy number variation algorithms developed for single-cell data, such as inferCNV (45) and copyKAT (44), may not be suitable for identifying malignant cells with low copy number variations, we did not use a copy number variation-based strategy in our study. Moreover, we included a wide range of brain tumors in our analysis. To ensure consistent analysis strategies for each tumor type and dataset, and to avoid discrepancies caused by different analysis approaches, we employed the clustering analysis combined with feature genes which demonstrated higher universality and achieved effective clustering. Our study provides an essential step towards fully understanding the TME in the major pediatric brain tumors before mechanically applying the current immunotherapy strategy on them.

Here, we revealed the distinct characteristics of the TME in the major tumor types (MB, EPN, IDH-mutation, and H3K27M-mutation) in children, which contained fewer immune cells than those in adults. Consistent with the previous study investigating the immunophenotypes of pediatric brain tumors via multicolor FACS, the EPN held higher infiltrating myeloid and T cells than MB (**Figure 1J**) (46). The strategy targeting the T cells has demonstrated non-persistent clinical responses in adult IDH-wild gliomas (47, 48). Although another study reported that Group 3 MB might respond better than SHH MB when applied with the PD-1 inhibitor in murine, the extremely low contents of T cells and expression percentage of PD-1 in MB and EPN suggested that the current strategy targeting the T cells in pediatric brain tumors should be more cautious (**Figures 1J**, **3F**) (49).

In spite of tremendous challenges of immunotherapy in pediatric brain tumors, some opportunities still exist. An unprecedented number of studies have demonstrated the myeloid lineage-associated resistance mechanisms in the resistance to therapy (50, 51). Developing the therapeutic strategies targeting myeloid subpopulations, as the dominated immune cells in the TME, is of enormous potential to complement the current immunotherapy strategies (52). For example, depleting CD73 in mice could decrease the immunosuppressive macrophage subset but increase the immunostimulatory subpopulations to enhance the anti-PD1 effectiveness (53). However, the diverse functions of myeloid cells are highly dependent on the different neuropathological conditions. Our study showed that classical cancer-related hallmarks in the myeloid of children and adult were highly heterogenous. The cytokine-enriched clusters were the shared myeloid type among the different tumors, indicating the common response to pathological conditions (**Figure 5A**) (9, 12, 54). Unlike those in adult brain, the myeloid subsets highly expressing interferon-related genes were not detected in children. Although we did not detect the clusters significantly highly expressing CD73, we uncovered a DC-like subpopulation highly expressing HLA genes and CD1E, which was associated with worse prognosis, and was an independent prognostic indicator of MB patient survival. This observation suggested that this myeloid subset was a potential target, but the strategy of targeting the myeloid subpopulation should be tailored according to their functional phenotypes in different tumor types.

Malignant cells are the major component of brain tumors. Parsing the relations among the immune and stromal components and tumor cells is essential to clearly understanding the feasibility of immunotherapy in pediatric brain tumors. In this study, we identified the common tumor-related features across the different tumor types which were correlated with the expression level of CD45 and found that they were significantly correlated with multiple immune cell types (**Figure 6J**). Interestingly, the negative immune recruitment and positive immune recruitment labeled the same tumor clusters (**Figure 6E**). Previous studies reported that cells often presented a dynamic equilibrium state of promoting or inhibiting a certain pathway or function, rather than simply promoting or inhibiting it (55, 56). In this study, we hope to quantify the immunosuppressive and immune recruitment ability of tumor cells in different tumor types through the gene feature scores. These tumor clusters exhibited synchronous immune recruitment and immune rejection features, indicating a close association between these tumor cells and the formation of the immune microenvironment. This association may have different effects on various types of immune cells, such as TAMs (57), T cells (58), and B cells (59).

The coarse classification-8 clusters, with distinct characteristics of myeloid, T-cell, and oligodendrocyte infiltration, indicated that the different traditional chemotherapy and immunotherapy strategies should be applied (**Figures 7G**, **S10G**). Interestingly, the integration of tumor-related features and myeloid subpopulation features helped to further discriminate the subsets potentially responding to the immune checkpoint inhibitors. Moreover, immune components including the cells, co-stimulators, and MHC molecules were extremely low in the clusters enriching tumor-rejected features (**Figure 8E**), suggesting the essentiality of fully considering the negative effect on the antitumor immunity and the potential roles of the malignant cells and myeloid subsets in the process of cancer immunology (60–62). Consistent with the previous studies, immunophenotypes of pediatric brain tumors may be less immunosuppressive than those of adult brain tumors, such as craniopharyngiomas and low-grade gliomas, which might light the path to the immunotherapy in pediatric brain tumors, especially for those tumors with high recurrence (**Figure 8G**) (46, 63, 64).

### 4.1 Limitations

While our research involved various single-cell datasets of pediatric brain tumors and conducted a thorough analysis of the tumor microenvironment, we regret that a more comprehensive subgroup analysis cannot be performed. This limitation stems from insufficient biological samples, limited size of single-cell data samples, and the absence of molecular subtyping labels for specific tumor types in publicly available datasets. In addition, unlike adult tumors, pediatric brain tumors have a wide variety but low incidence rates. Whether based on public databases or newly generated data from our own samples, the sample size is small. Furthermore, according to the previous studies, scRNA was able to identify higher percentages of immune cells (19). Therefore, the integration analysis was performed in order to maximize the sample size and neutralize the inconsistency of the two different sequencing platforms. However, the potential error caused by sequencing technology and algorithm factors is still unavoidable in our study. It is still worth further in-depth study.

## 5 Conclusions

In this study, we systematically compared the immunophenotypes of immune cells in the major pediatric brain tumors with those in adult gliomas by integrating the public and newly produced single-cell data and depicted the immune patterns in pediatric brain tumors. These results revealed that specific immune patterns might respond to the PD-1 or CTLA inhibitors. For the relative “cold” tumors, such as MB and EPN, targeting the myeloid subpopulations might also be a potential method.

## Data availability statement

The datasets presented in this study can be found in online repositories. The names of the repository/repositories and accession number(s) can be found in the article/**Supplementary Material**.

## Ethics statement

The studies involving humans were approved by Ethic committee of Xinhua Hospital Affiliated to Shanghai Jiao Tong University School of Medicine. The studies were conducted in accordance with the local legislation and institutional requirements. Written informed consent for participation in this study was provided by the participants’ legal guardians/next of kin. Written informed consent was obtained from the minor(s)’ legal guardian/next of kin for the publication of any potentially identifiable images or data included in this article.

## Author contributions

LC: methodology (lead), writing original draft (lead), data collection (lead). ST: data collection (equal), methodology (equal), writing of original draft (equal). WX: methodology (equal), writing of original draft (equal), HZ: data collection (equal); methodology (equal). ZL: sample collection. JW: methodology (equal). BW: methodology (equal), writing of review and editing (equal). JM: study design (lead); writing of review and editing (equal); funding acquisition (lead). All authors contributed to the article and approved the submitted version.

## Supplementary material

Supplementary Figure 1 The comparison of two methods-clustering-based (CB) method and positive expression of marker genes (PEMG) method. X axis represents the parameters of marker genes, cell types and corresponding marker pathways, and the y axis represents the ratio of the parameters between the two methods. The red line represents the mean ratio of all parameters.

Supplementary Figure 2
**(A)**. UMAP overlays of the feature scores and marker genes expression level of T cell subpopulations in the single cell dataset of Medulloblastoma. **(B)**. Kaplan–Meier survival curves generated with signature score of each T cell subpopulation in the medulloblastoma. **(C)**. UMAP overlays of the feature scores and marker genes expression level of T cell subpopulations in the single cell dataset of ependymoma. **(D)**. Identification of T cell subpopulations in the adult IDH-wild glioma. UMAP shows the identified T cell subsets, and dotplot shows the marker genes of each subpopulations. **(E)**. The integration of T cells from the different datasets GSE155446 (medulloblastoma), GSE125969 (ependymoma) and GSE182109 (adult IDH-wild glioma). **(F)**. The T cell subpopulations identified in the integrated T cell dataset. **(G)**. The marker genes of each identified subpopulation of T cell. **(H)**. The comparison of percentages of T cell subpopulations among the different tumors.

Supplementary Figure 3
**(A)**.The UMAP plot shows the clusters of myeloid compartments from the H3K27M-mutation based on smart-seq2. The dotplot displays the classical marker genes of MG and BMDM in each cluster. **(B)**. The UMAP plots respectively show the clusters of myeloid compartments from the adult IDH-wild gliomas based on smart-seq2 and displays the discrimination between MG and BMDM. The dotplot displays the classical marker genes of MG and BMDM in each cluster. **(C)**. The UMAP plots respectively show the clusters of myeloid compartments from the ependymoma based on smart-seq2 and displays the discrimination between MG and BMDM. The dotplot displays the classical marker genes of MG and BMDM in each cluster. **(D–F)**. Evaluation of MG and BMDM in the 10X data of medulloblastoma, adult IDH-wild glioma and IDH-mutation glioma. The UMAP plot displays the consistency between the predicted MG and BMDM and the clusters. The dotplot shows the expression of classical markers in the MG and BMDM. UMAP plots of marker genes shows the expression of classical markers in the cells. **(G)**. The UMAP plots shows the clusters of myeloid cells from the ependymoma, medulloblastoma, pediatric IDH-mutation glioma, adult IDH-mutation glioma and adult IDH-wild gliomas.

Supplementary Figure 4
**(A–D)**. The evaluation of myeloid subpopulations in ependymoma, medulloblastoma, pediatric IDH-mutation and adult IDH-mutation. The UMAP plots show the integration of the single cells from the different sources and the clusters of integrated myeloid datasets. The dotplots display the marker genes of each cluster. **(E)**. The UMAP plots shows the clusters of myeloid cells from the adult IDH-wild gliomas. **(F)**. The line charts show the optimal cluster number (dotted line) and the minimum value of Davies-Bouldin index (DBI) of different integrated myeloid datasets of each tumor type. X axis represents the cluster number, and Y axis represents the DBI.

Supplementary Figure 5
**(A)**. Heatmap display the phagocytosis and angiogenesis scores in each cluster across the different tumors. **(B)**. Dotplot shows the marker genes (HLA-DQA1 and CD1E) in the cluster2 of medulloblastoma. **(C)**. Kaplan–Meier survival curves generated with each clusters signature score of adult IDH-mutation glioma using the CGGA dataset. **(D)**. Kaplan–Meier survival curves generated with each clusters signature score of adult IDH-wild glioma using the CGGA dataset.

Supplementary Figure 6
**(A)**.The intersections of marker genes of major cell types across the different datasets. **(B–I)**. Upset plot reveals the intersections of gene features from scFes and other gene features from different sources**. (J, K)** Heatmap and hierarchical clustering of mean expression levels in the purified cell lines collected by previous study (34).

Supplementary Figure 7
**(A)**. UMAP overlays of the feature scores of major cell types in the independent single cell dataset of ependymoma. **(B)**. UMAP overlays of the feature scores of major cell types in the independent single cell dataset of adult IDH-wild glioma.

Supplementary Figure 8
**(A)**.The tumor cells in the medulloblastoma were sampled and the heatmap shows that the expression levels of negative-IR genes in the most of tumor cells are higher those of positive-IR genes. **(B–D)**. The positive-IP scores**(B)**, the negative-IR scores**(C)** and OG**(D)** scores were associated with the better prognosis.

Supplementary Figure 9
**(A)**. The line chart shows optimal cluster number (dotted line) and the minimum value of DBI. X axis represents the cluster number, and Y axis represents the DBI. **(B–D)**. The violin plots respectively show the expression levels of three markers among the 8 clusters. **(E)**. Heatmap and hierarchical clustering of median chemokine gene expression per cluster in the CBTTC cohort. **(F)**. Dotplot shows the median chemokine gene expression per cluster of tumor related processes in the CBTTC cohort. **(G)**. A heatmap showing top and bottom Hallmark Pathways with the top 20 variances among the 8 clusters.

Supplementary Figure 10
**(A)**. Heatmap and hierarchical clustering of CD4 and CD8 features from scFes. **(B)**. Heatmap and hierarchical clustering of CD4 and CD8 features from the recently published article(引文). **(C)**. The line chart shows optimal cluster number (dotted line) and the minimum value of DBI. X axis represents the cluster number, and Y axis represents the DBI. **(D)**. The heatmap shows the 12 clusters based on the 9 features via hierarchical clustering. **(E)**. The PCA plot reveals the batch effect among the different RNA-seq datasets from GEO and the removal of batch effect with the preservation of corresponding tumor characters. **(F)**. The heatmap shows the mean expression levels of the marker genes of predominant cell type in each cluster using the integrated GEO dataset **(G)**. The prediction of response rates in each cluster (8 clusters). **(H)**. The TIDE score among the different clusters (12 clusters).

## Glossary

**Table:**

TME	tumor microenvironment
CNS	center nervous system
scRNA-seq	single-cell RNA sequencing
MG	microglial
BMDM	bone marrow-derived myeloid
scFes	gene features based on single-cell data
BBB	blood–brain barrier
TAM	tumor-associated macrophage
P-IDH-M	pediatric IDH-mutation
IDH-W	IDH-wild
HVG	highly variable genes
PCA	principal component analysis
SNN	shared nearest neighbor
AA	anaplastic astrocytoma
MB	medulloblastoma
EPN	ependymoma
AS	astrocytoma
ATRT	atypical teratoid rhabdoid tumor
CPP	choroid plexus papilloma
CPG	craniopharyngioma
DIPG	diffuse intrinsic pontine glioma
EPN	ependymoma
GG	ganglioglioma
OG	oligodendrocyte
PNET	primitive neuroectodermal tumor
DEGs	differential expression genes
DBI	Davies–Bouldin Index
logFC	log fold change
CV	coefficient of variation
CB	clustering-based
PEMG	positive expression of marker genes
GBM	glioblastoma
mIHC	multiple immunohistochemistry
negative-PR	negative immune recruitment
positive-PR	positive immune recruitment
CD8 Tem	CD8 effector memory T cells
terminal CD8 Tem	CD8 terminal effector memory T cells
CD4 Tm	CD4 memory T cells
Treg	regulatory T cell
Tn	naive T cell
ISG	interferon-stimulated gene
