# Supplementary material for: The unique immune ecosystems in pediatric brain tumors: integrating single-cell and bulk RNA-sequencing
Source: Front Immunol. 2023 Nov 29;14:1238684. doi: 10.3389/fimmu.2023.1238684 (PMC10716463; doi:10.3389/fimmu.2023.1238684)
Supplement: Supplementary file 1 [file Table_1.docx]

| **Supplemental Table 1. Single cell Datasets included in this sudy** | | | | | | | | | |
| --- | --- | --- | --- | --- | --- | --- | --- | --- | --- |
| **Data Set** | **Tumor Type** | **Populations** | **Sample Number** | **Cell Counts** | **Application in this study** | | | | |
| GSE102130  (Smart-Seq)  (Filbin et al., 2020) | H3K27M-Mutation glioma | children | 6 | 2455 | analysis of immune components | \ | identification of MG and BMDM |  | construction of gene features |
| GSE70630  (Smart-Seq)  (Tirosh et al., 2016) | IDH-Mutation glioma | children | 6 | 4347 | analysis of immune components | \ | identification of MG and BMDM | myeloid immunophenotypes | construction of gene features |
| GSE89567  (Smart-Seq)  (Venteicher et al., 2017) | IDH-Mutation glioma | children | 11 | 5667 | analysis of immune components | \ | identification of MG and BMDM | myeloid immunophenotypes | construction of gene features |
| GSE138794  (10X-scRNAseq)  (Wang et al., 2019) | IDH-Wild glioma | adult | 5 | 6684 | \ | \ | \ | \ | construction of gene features |
| GSE182109  (10X-scRNAseq)  (  Abdelfattah et al., 2022) | IDH-Mutation glioma | adult | 4 | 21643 | analysis of immune components | T cell immunophenotypes | identification of MG and BMDM | myeloid immunophenotypes | validation of gene features |
| GSE155446  (10X-scRNAseq)  (Riemondy et al., 2022) | medulloblastoma | children | 28 | 39946 | analysis of immune components | T cell immunophenotypes | identification of MG and BMDM | myeloid immunophenotypes | construction of gene features |
| **Newly Produced**  (10X-snRNAseq) | medulloblastoma | children | 4 | 40972 | \ | T cell immunophenotypes | \ | myeloid immunophenotypes | validation of gene features |
| GSE141460  (10X-scRNAseq)  (Gojo et al., 2020) | ependymoma | children | 6 | 66847 | \ | T cell immunophenotypes | \ | myeloid immunophenotypes | construction of gene features |
| GSE141460 (Smart-Seq)  (Gojo et al., 2020) | ependymoma | children | 22 | 6441 | \ | \ | \ | \ | construction of gene features |
| GSE125969  (10X-scRNAseq)  (Gillen et al., 2020) | ependymoma | children | 26 | 18500 | analysis of immune components | T cell immunophenotypes | identification of MG and BMDM | myeloid immunophenotypes | validation of gene features |
| GSE131928 (10X-scRNAseq)  (Neftel et al., 2020) | IDH-Wild glioma | adult | 10 | 16201 | \ | \ | \ | \ | construction of gene features |
| GSE131928 (Smart-Seq)  (Neftel et al., 2020) | IDH-Wild glioma | adult | 18 | 7930 | \ | \ | \ | \ | construction of gene features |
| GSE138794  (10X-scRNAseq)  (Wang et al., 2019) | IDH-Wild glioma | adult | 4 | 9968 | \ | \ | \ | \ | construction of gene features |
| GSE147275  (10X-scRNAseq)  (Zamler et al., 2022) | IDH-Wild glioma | adult | 10 | 6918 | \ | \ | \ | \ | construction of gene features |
| GSE182109  (10X-scRNAseq)  (Abdelfattah et al., 2022) | IDH-Wild glioma | adult | 11 | 69929 | analysis of immune components | T cell immunophenotypes | identification of MG and BMDM | myeloid immunophenotypes | validation of gene features |

Abdelfattah N, Kumar P, Wang C, Leu JS et al. Single-cell analysis of human glioma and immune cells identifies S100A4 as an immunotherapy target. Nat Commun 2022 Feb 9;13(1):767. PMID: 35140215.

Filbin MG, Tirosh I, Hovestadt V, Shaw ML et al. Developmental and oncogenic programs in H3K27M gliomas dissected by single-cell RNA-seq. Science 2018 Apr 20;360(6386):331-335. PMID: 29674595

Gillen AE, Riemondy KA, Amani V, Griesinger AM et al. Single-Cell RNA Sequencing of Childhood Ependymoma Reveals Neoplastic Cell Subpopulations That Impact Molecular Classification and Etiology. Cell Rep 2020 Aug 11;32(6):108023. PMID: 32783945

Gojo J, Englinger B, Jiang L, Hübner JM et al. Single-Cell RNA-Seq Reveals Cellular Hierarchies and Impaired Developmental Trajectories in Pediatric Ependymoma. Cancer Cell 2020 Jul 13;38(1):44-59.e9. PMID: 32663469

Neftel C, Laffy J, Filbin MG, Hara T et al. An Integrative Model of Cellular States, Plasticity, and Genetics for Glioblastoma. Cell 2019 Aug 8;178(4):835-849.e21. PMID: 31327527

Riemondy KA, Venkataraman S, Willard N, Nellan A et al. Neoplastic and immune single-cell transcriptomics define subgroup-specific intra-tumoral heterogeneity of childhood medulloblastoma. Neuro Oncol 2022 Feb 1;24(2):273-286. PMID: 34077540

Tirosh I, Venteicher AS, Hebert C, Escalante LE et al. Single-cell RNA-seq supports a developmental hierarchy in human oligodendroglioma. Nature 2016 Nov 10;539(7628):309-313. PMID: 27806376

Venteicher AS, Tirosh I, Hebert C, Yizhak K et al. Decoupling genetics, lineages, and microenvironment in IDH-mutant gliomas by single-cell RNA-seq. Science 2017 Mar 31;355(6332). PMID: 28360267

Wang L, Babikir H, Müller S, Yagnik G et al. The Phenotypes of Proliferating Glioblastoma Cells Reside on a Single Axis of Variation. Cancer Discov 2019 Dec;9(12):1708-1719. PMID: 31554641

Wang L, Babikir H, Müller S, Yagnik G et al. The Phenotypes of Proliferating Glioblastoma Cells Reside on a Single Axis of Variation. Cancer Discov 2019 Dec;9(12):1708-1719. PMID: 31554641

Zamler DB, Shingu T, Kahn LM, Huntoon K et al. Immune landscape of a genetically engineered murine model of glioma compared with human glioma. JCI Insight 2022 Jun 22;7(12). PMID: 35653194

| **Supplemental Table 2. Gene features of major cell types(scFes)** | | | | | | | | | | | | | | | | | | | | | | | | | | | |
| --- | --- | --- | --- | --- | --- | --- | --- | --- | --- | --- | --- | --- | --- | --- | --- | --- | --- | --- | --- | --- | --- | --- | --- | --- | --- | --- | --- |
| **Endo** | | **OG** | **NK** | **T cell** | | **B cell** | **Myeloid** | **CD4 T** | **CD8 T** | **Treg** | **CD4 Tm** | | **CD8 Tem** | **BAM** | | **MG** | **BMDM** | **Monocyte** | **cDC1** | | **cDC2** | **Plasma** | **Pericyte** | | **Tumor Cell** | **Positive IR** | **Negative ir** |
| CTGF | | MAG | CD247 | CD3D | | IGKC | C3 | CD4 | CD8A | IL2RA | IL7R | | CD8A | F13A1 | | C3 | S100A10 | CSTA | WDFY4 | | FCER1A | DERL3 | RGS5 | | CNN3 | CNN3 | NRXN1 |
| GNG11 | | MOBP | GNLY | CD52 | | JCHAIN | MSR1 | CD40LG | CD8B | FOXP3 | CD52 | | CD8B | MPEG1 | | TREM2 | S100A6 | FCN1 | CLNK | | CD1E | CD38 | SLC38A11 | | GPM6A | DPYSL3 | MAP2 |
| MFSD2A | | PPP1R14A | SPON2 | IL7R | | BANK1 | CSF1R | IL7R | NKG7 | IL32 | S100A4 | | GZMK | MS4A6A | | EPB41L2 | VIM | S100A8 | C1orf54 | | CD1C | MZB1 | MYO1B | | MAGED1 | JAM2 | SCG3 |
| IGFBP3 | | ERMN | FGFBP2 | GZMK | | MS4A1 | HLA-DRB1 | KLRB1 | CCL5 | BATF | TPT1 | | GZMA | MS4A4A | | SLC1A3 | ANXA1 | S100A9 | CLEC9A | | CLEC10A | JCHAIN | CARMN | | MAP2 |  | CHGB |
| IGFBP7 | | CNDP1 | CLIC3 | CD3G | | TCL1A | HLA-DRA | SELL | CTSW | TNFRSF18 | SPOCK2 | | GZMH | MRC1 | | SCIN | EMP3 | CFP | IDO1 | |  | IGHG1 | FHL5 | | PTPRS |  | TTC3 |
| ADIRF | | KLK6 | PRF1 | IL32 | | IGHD | PLXDC2 |  | KLRD1 | CD27 |  | | CCL5 | CD163 | | PADI2 | TAGLN2 | FGR |  | |  | IGHGP | LAMC3 | | DBN1 |  | GPM6A |
| CLDN5 | | MBP | KLRB1 | CD2 | | IGHM | CTSB |  | GZMK | TIGIT |  | |  | MS4A7 | | MEF2C | ANXA2 | NCF2 |  | |  | IGLL5 | PLXDC1 | | TSPAN7 |  | MAGED1 |
| ITM2A | |  | KLRF1 |  | | CD79A |  |  | GZMH | UGP2 |  | |  |  | | FRMD4A | CD44 | LYZ |  | |  |  | GJC1 | | DPP6 |  | PTPRS |
| IFI27 | |  | KLRD1 |  | | IGLC2 |  |  | ZNF683 | CCR8 |  | |  |  | | ST6GAL1 | S100A4 | CFD |  | |  |  | NOTCH3 | | DPYSL3 |  | DBN1 |
|  | |  | GZMB |  | | IGLC3 |  |  | ITM2C |  |  | |  |  | | LPCAT2 | LYZ | LILRB3 |  | |  |  | TFPI | | MDK |  | TSPAN7 |
|  | |  | CST7 |  | | TNFRSF13C |  |  | XCL2 |  |  | |  |  | | OLR1 | SH3BP5 | S100A4 |  | |  |  | LHFPL6 | | MLLT11 |  | DPP6 |
|  | |  | NKG7 |  | | RALGPS2 |  |  |  |  |  | |  |  | |  | TMSB10 | S100A6 |  | |  |  | CCDC3 | | GRIA2 |  | MLLT11 |
|  | |  | CD7 |  | | CD79B |  |  |  |  |  | |  |  | |  | LCP1 |  |  | |  |  | SMOC2 | | GOLM1 |  | GRIA2 |
|  | |  | ARL4C |  | | IGHA1 |  |  |  |  |  | |  |  | |  | CSGALNACT2 |  |  | |  |  | PDGFRB | | SCG3 |  | SEZ6L |
|  | |  | PTGDS |  | | LTB |  |  |  |  |  | |  |  | |  |  |  |  | |  |  | FN1 | | CRMP1 |  | ABAT |
|  | |  | PLAC8 |  | | CD37 |  |  |  |  |  | |  |  | |  |  |  |  | |  |  | ATP1A2 | | TTC3 |  |  |
|  | |  |  |  | |  |  |  |  |  |  | |  |  | |  |  |  |  | |  |  | CDH6 | | SEZ6 |  |  |
|  | |  |  |  | |  |  |  |  |  |  | |  |  | |  |  |  |  | |  |  | FILIP1L | | ABAT |  |  |
|  | |  |  |  | |  |  |  |  |  |  | |  |  | |  |  |  |  | |  |  | CALD1 | | JAM2 |  |  |
|  | |  |  |  | |  |  |  |  |  |  | |  |  | |  |  |  |  | |  |  | EPS8 | | NRXN1 |  |  |
|  | |  |  |  | |  |  |  |  |  |  | |  |  | |  |  |  |  | |  |  |  | | CHGB |  |  |
|  | |  |  |  | |  |  |  |  |  |  | |  |  | |  |  |  |  | |  |  |  | | RCN1 |  |  |
| **Supplemental Table 3. Immune patterns in CBTTC cohort** | | | | | | | | | | | | | | | | | | | | | | |  |  |  |  |  |
| **sample** | | | | **Histological Diagnosis (Source Text)** | | | | | | | **Age(days)** | | | **9features-immune paterns** | | | | | **3features-immune paterns** | | | |  |  |  |  |  |
| PT_00G007DM_7316-272 | | | | Medulloblastoma | | | | | | | 464 | | | Immune Desert Tumor Rejected | | | | | Immune Desert | | | |  |  |  |  |  |
| PT_02J5CWN5_7316-2989 | | | | Low-grade glioma/astrocytoma (WHO grade I/II) | | | | | | | 2223 | | | Immune Stromal Rich | | | | | Immune Stromal Rich | | | |  |  |  |  |  |
| PT_02J5CWN5_7316-898 | | | | Low-grade glioma/astrocytoma (WHO grade I/II) | | | | | | | 3722 | | | Immune Rich Tumor Recruitment | | | | | Immune Rich | | | |  |  |  |  |  |
| PT_04YTK82N_7316-746 | | | | Medulloblastoma | | | | | | | 1067 | | | Immune Desert Tumor Rejected | | | | | Immune Desert | | | |  |  |  |  |  |
| PT_06H29FCG_7316-1944 | | | | Ependymoma | | | | | | | 813 | | | Tumor Recruitment BMDM bias | | | | | Immune Rich | | | |  |  |  |  |  |
| PT_0CE0HFYB_7316-2581 | | | | Low-grade glioma/astrocytoma (WHO grade I/II) | | | | | | | 3747 | | | Immune Rich Tumor Recruitment | | | | | Immune Rich | | | |  |  |  |  |  |
| PT_0CRPE7YP_7316-2158 | | | | Craniopharyngioma | | | | | | | 3004 | | | Stromal Centered Myeloid bias | | | | | Immune Stromal Rich | | | |  |  |  |  |  |
| PT_0CVRX4SJ_7316-764 | | | | Ependymoma | | | | | | | 766 | | | Immune Desert | | | | | Immune Desert | | | |  |  |  |  |  |
| PT_0DWRY9ZX_7316-2221 | | | | Medulloblastoma | | | | | | | 3804 | | | Immune Desert Tumor Rejected | | | | | Immune Desert | | | |  |  |  |  |  |
| PT_0DXHDZJR_7316-2857 | | | | High-grade glioma/astrocytoma (WHO grade III/IV) | | | | | | | 493 | | | Immune Desert | | | | | Immune Desert | | | |  |  |  |  |  |
| PT_0EWY6S6S_7316-3491 | | | | Low-grade glioma/astrocytoma (WHO grade I/II) | | | | | | | 3999 | | | Immune Stromal Rich | | | | | Immune Stromal Rich | | | |  |  |  |  |  |
| PT_0K0DPVF6_7316-121 | | | | Low-grade glioma/astrocytoma (WHO grade I/II) | | | | | | | 2480 | | | Immune Stromal Myeloid bias | | | | | Myeloid Centric | | | |  |  |  |  |  |
| PT_0MHKP408_7316-726 | | | | Low-grade glioma/astrocytoma (WHO grade I/II) | | | | | | | 2581 | | | Immune Stromal Rich | | | | | Immune Stromal Rich | | | |  |  |  |  |  |
| PT_0MJEAART_7316-549 | | | | Brainstem glioma- Diffuse intrinsic pontine glioma | | | | | | | 4098 | | | Tumor Recruitment BMDM bias | | | | | Immune Stromal Rich | | | |  |  |  |  |  |
| PT_0NY38X3W_7316-1706 | | | | Ependymoma | | | | | | | 563 | | | Tumor Recruitment BMDM bias | | | | | Immune Desert | | | |  |  |  |  |  |
| PT_0PT33SCF_7316-1100 | | | | Supratentorial or Spinal Cord PNET | | | | | | | 1454 | | | Immune Desert Tumor Rejected | | | | | Immune Desert | | | |  |  |  |  |  |
| PT_0SPKM4S8_7316-2640 | | | | High-grade glioma/astrocytoma (WHO grade III/IV) | | | | | | | 2005 | | | Immune Rich | | | | | Immune Rich | | | |  |  |  |  |  |
| PT_0WKX8Q5X_7316-88 | | | | Ependymoma | | | | | | | 5358 | | | Immune Rich Tumor Recruitment | | | | | Immune Rich | | | |  |  |  |  |  |
| PT_0WMP712K_7316-922 | | | | Medulloblastoma | | | | | | | 494 | | | Immune Rich | | | | | T cell Centric | | | |  |  |  |  |  |
| PT_12JTZFC2_7316-3504 | | | | Low-grade glioma/astrocytoma (WHO grade I/II) | | | | | | | 1615 | | | Immune Rich Tumor Rich | | | | | Immune Rich | | | |  |  |  |  |  |
| PT_13MKXR8G_7316-2251 | | | | Choroid plexus papilloma | | | | | | | 3470 | | | Tumor Rejected cDC2 bias | | | | | Immune Desert | | | |  |  |  |  |  |
| PT_164RNWTT_7316-1078 | | | | Ependymoma | | | | | | | 2549 | | | Tumor Rejected cDC2 bias | | | | | T cell Centric | | | |  |  |  |  |  |
| PT_17NG0KW3_7316-1945 | | | | Medulloblastoma | | | | | | | 2513 | | | Immune Desert | | | | | Immune Desert | | | |  |  |  |  |  |
| PT_17W3GJPT_7316-2306 | | | | Ganglioglioma | | | | | | | 663 | | | Stromal Centered Myeloid bias | | | | | Immune Stromal Desert | | | |  |  |  |  |  |
| PT_18QG3Z4H_7316-1052 | | | | High-grade glioma/astrocytoma (WHO grade III/IV) | | | | | | | 4098 | | | Stromal Centered Myeloid bias | | | | | Immune Desert | | | |  |  |  |  |  |
| PT_1B1Z7BRQ_7316-287 | | | | High-grade glioma/astrocytoma (WHO grade III/IV) | | | | | | | 5284 | | | Tumor Rejected cDC2 bias | | | | | T cell Centric | | | |  |  |  |  |  |
| PT_1BV6ND1D_7316-3203 | | | | Low-grade glioma/astrocytoma (WHO grade I/II) | | | | | | | 1453 | | | Immune Stromal Myeloid bias | | | | | Immune Stromal Rich | | | |  |  |  |  |  |
| PT_1DSX4VJM_7316-1708 | | | | Brainstem glioma- Diffuse intrinsic pontine glioma | | | | | | | 2627 | | | Tumor Rejected OG bias | | | | | Immune Stromal Desert | | | |  |  |  |  |  |
| PT_1J2DT6MM_7316-2184 | | | | Low-grade glioma/astrocytoma (WHO grade I/II) | | | | | | | 3602 | | | Immune Stromal Myeloid bias | | | | | Immune Stromal Rich | | | |  |  |  |  |  |
| PT_1KEER2Q1_7316-460 | | | | Low-grade glioma/astrocytoma (WHO grade I/II) | | | | | | | 3826 | | | Immune Rich | | | | | Immune Rich | | | |  |  |  |  |  |
| PT_1MW98VR1_7316-2558 | | | | Ependymoma | | | | | | | 1872 | | | Tumor Recruitment BMDM bias | | | | | Immune Rich | | | |  |  |  |  |  |
| PT_1N6ATKJS_7316-2725 | | | | Low-grade glioma/astrocytoma (WHO grade I/II) | | | | | | | 4940 | | | Immune Stromal Tumor Recruitment | | | | | Immune Stromal Rich | | | |  |  |  |  |  |
| PT_1NZGDP3P_7316-1104 | | | | Medulloblastoma | | | | | | | 882 | | | Tumor Rejected cDC2 bias | | | | | T cell Centric | | | |  |  |  |  |  |
| PT_1T7WF5S1_7316-2934 | | | | Medulloblastoma | | | | | | | 1846 | | | Tumor Rejected cDC2 bias | | | | | Immune Desert | | | |  |  |  |  |  |
| PT_1TGKS073_7316-1659 | | | | Low-grade glioma/astrocytoma (WHO grade I/II) | | | | | | | 1314 | | | Immune Stromal Rich | | | | | Immune Stromal Rich | | | |  |  |  |  |  |
| PT_1Z015H6V_7316-3516 | | | | Ependymoma | | | | | | | 6307 | | | Tumor Recruitment BMDM bias | | | | | Immune Desert | | | |  |  |  |  |  |
| PT_1ZAWNGWT_7316-3345 | | | | Ganglioglioma | | | | | | | 6215 | | | Tumor Rejected OG bias | | | | | Immune Stromal Desert | | | |  |  |  |  |  |
| PT_20ZM6THA_7316-3629 | | | | Low-grade glioma/astrocytoma (WHO grade I/II) | | | | | | | 1889 | | | Immune Rich Tumor Rich | | | | | Immune Rich | | | |  |  |  |  |  |
| PT_218SX3G4_7316-529 | | | | Supratentorial or Spinal Cord PNET | | | | | | | 480 | | | Tumor Rejected cDC2 bias | | | | | T cell Centric | | | |  |  |  |  |  |
| PT_23K3YS1P_7316-189 | | | | Ependymoma | | | | | | | 1808 | | | Immune Desert Tumor Rejected | | | | | Immune Desert | | | |  |  |  |  |  |
| PT_23NZGSRJ_7316-2659 | | | | Medulloblastoma | | | | | | | 1484 | | | Immune Desert | | | | | Immune Desert | | | |  |  |  |  |  |
| PT_24DDXEB5_7316-1715 | | | | Medulloblastoma | | | | | | | 2678 | | | Immune Desert Tumor Rejected | | | | | Immune Desert | | | |  |  |  |  |  |
| PT_26V72ZXB_7316-3576 | | | | Medulloblastoma | | | | | | | 4170 | | | Tumor Rejected cDC2 bias | | | | | Immune Desert | | | |  |  |  |  |  |
| PT_27FDWCVZ_7316-184 | | | | Low-grade glioma/astrocytoma (WHO grade I/II) | | | | | | | 3295 | | | Immune Stromal Tumor Recruitment | | | | | Immune Stromal Rich | | | |  |  |  |  |  |
| PT_2BJF83GQ_7316-1745 | | | | Low-grade glioma/astrocytoma (WHO grade I/II) | | | | | | | 3531 | | | Immune Stromal Tumor Recruitment | | | | | Immune Stromal Rich | | | |  |  |  |  |  |
| PT_2BTC5TEN_7316-2183 | | | | Craniopharyngioma | | | | | | | 3529 | | | Immune Rich | | | | | Immune Rich | | | |  |  |  |  |  |
| PT_2C63FJAM_7316-3040 | | | | Low-grade glioma/astrocytoma (WHO grade I/II) | | | | | | | 3825 | | | Immune Desert | | | | | Immune Desert | | | |  |  |  |  |  |
| PT_2CJ31SBX_7316-3624 | | | | Ependymoma | | | | | | | 1364 | | | Tumor Recruitment BMDM bias | | | | | Immune Desert | | | |  |  |  |  |  |
| PT_2ECVKTTQ_7316-173 | | | | Low-grade glioma/astrocytoma (WHO grade I/II) | | | | | | | 4937 | | | Immune Rich Tumor Recruitment | | | | | Immune Rich | | | |  |  |  |  |  |
| PT_2ECVKTTQ_7316-2154 | | | | Low-grade glioma/astrocytoma (WHO grade I/II) | | | | | | | 6061 | | | Immune Stromal Tumor Recruitment | | | | | Immune Stromal Rich | | | |  |  |  |  |  |
| PT_2FK75B27_7316-1700 | | | | Medulloblastoma | | | | | | | 2538 | | | Immune Desert Tumor Rejected | | | | | Immune Desert | | | |  |  |  |  |  |
| PT_2FVTD0WR_7316-2897 | | | | Low-grade glioma/astrocytoma (WHO grade I/II) | | | | | | | 1528 | | | Immune Stromal Tumor Recruitment | | | | | Immune Stromal Rich | | | |  |  |  |  |  |
| PT_2GPH62JS_7316-1775 | | | | Craniopharyngioma | | | | | | | 4994 | | | Immune Rich | | | | | Immune Rich | | | |  |  |  |  |  |
| PT_2JDDX6TJ_7316-409 | | | | High-grade glioma/astrocytoma (WHO grade III/IV) | | | | | | | 4253 | | | Immune Rich | | | | | Immune Stromal Rich | | | |  |  |  |  |  |
| PT_2NQPX1R2_7316-1116 | | | | Craniopharyngioma | | | | | | | 5948 | | | Tumor Rejected cDC2 bias | | | | | T cell Centric | | | |  |  |  |  |  |
| PT_2PRV78FX_7316-176 | | | | Low-grade glioma/astrocytoma (WHO grade I/II) | | | | | | | 1389 | | | Immune Desert | | | | | Myeloid Centric | | | |  |  |  |  |  |
| PT_2QZJ4T95_7316-715 | | | | Brainstem glioma- Diffuse intrinsic pontine glioma | | | | | | | 3035 | | | Tumor Rejected OG bias | | | | | Immune Stromal Desert | | | |  |  |  |  |  |
| PT_2SCVQ2N6_7316-3631 | | | | Ependymoma | | | | | | | 4770 | | | Immune Rich | | | | | Immune Rich | | | |  |  |  |  |  |
| PT_2VSDT9CK_7316-3575 | | | | Low-grade glioma/astrocytoma (WHO grade I/II) | | | | | | | 1956 | | | Immune Stromal Myeloid bias | | | | | Myeloid stromal Centric | | | |  |  |  |  |  |
| PT_2WA5PM32_7316-949 | | | | Craniopharyngioma | | | | | | | 2927 | | | Immune Rich | | | | | Immune Rich | | | |  |  |  |  |  |
| PT_2WVW55DA_7316-1890 | | | | Low-grade glioma/astrocytoma (WHO grade I/II) | | | | | | | 584 | | | Immune Rich Tumor Recruitment | | | | | Immune Rich | | | |  |  |  |  |  |
| PT_2WVW55DA_7316-2092 | | | | High-grade glioma/astrocytoma (WHO grade III/IV) | | | | | | | 970 | | | Tumor Recruitment BMDM bias | | | | | Immune Rich | | | |  |  |  |  |  |
| PT_2WVW55DA_7316-2100 | | | | High-grade glioma/astrocytoma (WHO grade III/IV) | | | | | | | 994 | | | Tumor Recruitment BMDM bias | | | | | Immune Rich | | | |  |  |  |  |  |
| PT_2WVW55DA_7316-502 | | | | Low-grade glioma/astrocytoma (WHO grade I/II) | | | | | | | 245 | | | Immune Rich Tumor Recruitment | | | | | Immune Rich | | | |  |  |  |  |  |
| PT_2YCHME6S_7316-2622 | | | | Low-grade glioma/astrocytoma (WHO grade I/II) | | | | | | | 4797 | | | Tumor Recruitment BMDM bias | | | | | T cell stromal Centric | | | |  |  |  |  |  |
| PT_2YT37G8P_7316-2962 | | | | Low-grade glioma/astrocytoma (WHO grade I/II) | | | | | | | 1310 | | | Immune Rich Tumor Rich | | | | | Immune Rich | | | |  |  |  |  |  |
| PT_2YT37G8P_7316-399 | | | | Low-grade glioma/astrocytoma (WHO grade I/II) | | | | | | | 4304 | | | Immune Rich Tumor Rich | | | | | Immune Rich | | | |  |  |  |  |  |
| PT_2Z7GVDTM_7316-3515 | | | | Low-grade glioma/astrocytoma (WHO grade I/II) | | | | | | | 4633 | | | Immune Stromal Myeloid bias | | | | | Immune Stromal Rich | | | |  |  |  |  |  |
| PT_2ZTXV44P_7316-310 | | | | Medulloblastoma | | | | | | | 1889 | | | Tumor Rejected OG bias | | | | | Immune Stromal Desert | | | |  |  |  |  |  |
| PT_2ZXR2PDN_7316-1636 | | | | Ganglioglioma | | | | | | | 3941 | | | Tumor Rejected OG bias | | | | | Immune Stromal Desert | | | |  |  |  |  |  |
| PT_30H1KV15_7316-1059 | | | | High-grade glioma/astrocytoma (WHO grade III/IV) | | | | | | | 4880 | | | Tumor Recruitment BMDM bias | | | | | T cell Centric | | | |  |  |  |  |  |
| PT_32J909WM_7316-2688 | | | | Atypical Teratoid Rhabdoid Tumor (ATRT) | | | | | | | 860 | | | Tumor Recruitment BMDM bias | | | | | Immune Stromal Rich | | | |  |  |  |  |  |
| PT_37B5JRP1_7316-2217 | | | | High-grade glioma/astrocytoma (WHO grade III/IV) | | | | | | | 5324 | | | Stromal Centered Myeloid bias | | | | | Immune Stromal Desert | | | |  |  |  |  |  |
| PT_37B5JRP1_7316-2554 | | | | High-grade glioma/astrocytoma (WHO grade III/IV) | | | | | | | 5746 | | | Tumor Recruitment BMDM bias | | | | | Immune Desert | | | |  |  |  |  |  |
| PT_387VK1T9_7316-3625 | | | | High-grade glioma/astrocytoma (WHO grade III/IV) | | | | | | | 70 | | | Tumor Recruitment BMDM bias | | | | | Immune Desert | | | |  |  |  |  |  |
| PT_38FS37DP_7316-2686 | | | | Supratentorial or Spinal Cord PNET | | | | | | | 1053 | | | Immune Desert | | | | | Immune Desert | | | |  |  |  |  |  |
| PT_39HBKSGG_7316-908 | | | | Medulloblastoma | | | | | | | 4854 | | | Immune Desert Tumor Rejected | | | | | Immune Desert | | | |  |  |  |  |  |
| PT_3A2Q62RD_7316-2243 | | | | Ependymoma | | | | | | | 2184 | | | Tumor Recruitment BMDM bias | | | | | Immune Rich | | | |  |  |  |  |  |
| PT_3C6QA9MA_7316-1279 | | | | Ependymoma | | | | | | | 3294 | | | Tumor Rejected cDC2 bias | | | | | T cell stromal Centric | | | |  |  |  |  |  |
| PT_3CHB9PK5_7316-2085 | | | | High-grade glioma/astrocytoma (WHO grade III/IV) | | | | | | | 3455 | | | Tumor Recruitment BMDM bias | | | | | Immune Desert | | | |  |  |  |  |  |
| PT_3CHB9PK5_7316-515 | | | | High-grade glioma/astrocytoma (WHO grade III/IV) | | | | | | | 2908 | | | Stromal Centered Myeloid bias | | | | | Myeloid stromal Centric | | | |  |  |  |  |  |
| PT_3CVMBGD7_7316-1963 | | | | Low-grade glioma/astrocytoma (WHO grade I/II) | | | | | | | 3815 | | | Immune Stromal Myeloid bias | | | | | Myeloid stromal Centric | | | |  |  |  |  |  |
| PT_3HRS5CWF_7316-925 | | | | Low-grade glioma/astrocytoma (WHO grade I/II) | | | | | | | 779 | | | Immune Stromal Rich | | | | | Immune Stromal Rich | | | |  |  |  |  |  |
| PT_3J0ASVHN_7316-3622 | | | | Low-grade glioma/astrocytoma (WHO grade I/II) | | | | | | | 946 | | | Immune Stromal Rich | | | | | Immune Stromal Rich | | | |  |  |  |  |  |
| PT_3KK1F95W_7316-1744 | | | | Low-grade glioma/astrocytoma (WHO grade I/II) | | | | | | | 4633 | | | Tumor Rejected OG bias | | | | | T cell stromal Centric | | | |  |  |  |  |  |
| PT_3KM9W8S8_7316-1771 | | | | Atypical Teratoid Rhabdoid Tumor (ATRT) | | | | | | | 190 | | | Immune Desert Tumor Rejected | | | | | Immune Desert | | | |  |  |  |  |  |
| PT_3KM9W8S8_7316-3045 | | | | Atypical Teratoid Rhabdoid Tumor (ATRT) | | | | | | | 713 | | | Tumor Recruitment BMDM bias | | | | | T cell stromal Centric | | | |  |  |  |  |  |
| PT_3KWSRWZN_7316-2648 | | | | Ependymoma | | | | | | | 4590 | | | Immune Desert | | | | | Immune Desert | | | |  |  |  |  |  |
| PT_3MNY9BRY_7316-2583 | | | | Low-grade glioma/astrocytoma (WHO grade I/II) | | | | | | | 4077 | | | Immune Stromal Tumor Recruitment | | | | | Immune Stromal Rich | | | |  |  |  |  |  |
| PT_3NM45TQ8_7316-1077 | | | | Ganglioglioma | | | | | | | 2716 | | | Tumor Rejected OG bias | | | | | T cell stromal Centric | | | |  |  |  |  |  |
| PT_3PK66TJY_7316-2609 | | | | Ependymoma | | | | | | | 629 | | | Tumor Recruitment BMDM bias | | | | | Immune Rich | | | |  |  |  |  |  |
| PT_3R0P995B_7316-873 | | | | Ependymoma | | | | | | | 481 | | | Tumor Recruitment BMDM bias | | | | | Immune Desert | | | |  |  |  |  |  |
| PT_3R0P995B_7316-892 | | | | Ependymoma | | | | | | | 576 | | | Immune Desert | | | | | Immune Desert | | | |  |  |  |  |  |
| PT_3RN24N9X_7316-20 | | | | Low-grade glioma/astrocytoma (WHO grade I/II) | | | | | | | 1469 | | | Immune Stromal Myeloid bias | | | | | Myeloid Centric | | | |  |  |  |  |  |
| PT_3SW2VHD5_7316-2984 | | | | Low-grade glioma/astrocytoma (WHO grade I/II) | | | | | | | 4691 | | | Immune Rich | | | | | Immune Stromal Rich | | | |  |  |  |  |  |
| PT_3T3VGWC6_7316-375 | | | | Low-grade glioma/astrocytoma (WHO grade I/II) | | | | | | | 4493 | | | Immune Stromal Myeloid bias | | | | | Myeloid stromal Centric | | | |  |  |  |  |  |
| PT_3T3VGWC6_7316-3923 | | | | Low-grade glioma/astrocytoma (WHO grade I/II) | | | | | | | 5955 | | | Immune Stromal Tumor Recruitment | | | | | Immune Stromal Rich | | | |  |  |  |  |  |
| PT_3V5GTCJR_7316-1646 | | | | Low-grade glioma/astrocytoma (WHO grade I/II) | | | | | | | 3371 | | | Tumor Rejected OG bias | | | | | Immune Stromal Desert | | | |  |  |  |  |  |
| PT_3VCS1PPF_7316-198 | | | | Ependymoma | | | | | | | 4710 | | | Tumor Recruitment BMDM bias | | | | | Immune Rich | | | |  |  |  |  |  |
| PT_3VCS1PPF_7316-490 | | | | Ependymoma | | | | | | | 5233 | | | Tumor Recruitment BMDM bias | | | | | Immune Desert | | | |  |  |  |  |  |
| PT_3VQEVNDD_7316-1680 | | | | Medulloblastoma | | | | | | | 722 | | | Tumor Rejected cDC2 bias | | | | | Immune Stromal Desert | | | |  |  |  |  |  |
| PT_3WA1XB8G_7316-70 | | | | Ganglioglioma | | | | | | | 6528 | | | Stromal Centered Myeloid bias | | | | | T cell stromal Centric | | | |  |  |  |  |  |
| PT_3WA7SBQ6_7316-2248 | | | | Choroid plexus carcinoma | | | | | | | 698 | | | Immune Desert | | | | | Immune Desert | | | |  |  |  |  |  |
| PT_409Y4N1N_7316-84 | | | | Low-grade glioma/astrocytoma (WHO grade I/II) | | | | | | | 4334 | | | Stromal Centered Myeloid bias | | | | | Immune Stromal Rich | | | |  |  |  |  |  |
| PT_40HTPY49_7316-466 | | | | High-grade glioma/astrocytoma (WHO grade III/IV) | | | | | | | 2606 | | | Stromal Centered Myeloid bias | | | | | Myeloid stromal Centric | | | |  |  |  |  |  |
| PT_4159VCJY_7316-389 | | | | Ganglioglioma | | | | | | | 5094 | | | Stromal Centered Myeloid bias | | | | | Immune Stromal Desert | | | |  |  |  |  |  |
| PT_41BCPB7R_7316-406 | | | | Choroid plexus carcinoma | | | | | | | 864 | | | Tumor Recruitment BMDM bias | | | | | Immune Desert | | | |  |  |  |  |  |
| PT_41QYPSGV_7316-884 | | | | Low-grade glioma/astrocytoma (WHO grade I/II) | | | | | | | 5203 | | | Immune Rich Tumor Recruitment | | | | | Immune Rich | | | |  |  |  |  |  |
| PT_41WETNRC_7316-3065 | | | | Low-grade glioma/astrocytoma (WHO grade I/II) | | | | | | | 978 | | | Immune Rich Tumor Rich | | | | | Immune Rich | | | |  |  |  |  |  |
| PT_44HPG5YT_7316-3067 | | | | Ganglioglioma | | | | | | | 301 | | | Stromal Centered Myeloid bias | | | | | Myeloid stromal Centric | | | |  |  |  |  |  |
| PT_45P9F9MD_7316-314 | | | | Medulloblastoma | | | | | | | 3856 | | | Immune Desert Tumor Rejected | | | | | Immune Desert | | | |  |  |  |  |  |
| PT_45YH276T_7316-499 | | | | Low-grade glioma/astrocytoma (WHO grade I/II) | | | | | | | 623 | | | Immune Rich Tumor Rich | | | | | Immune Rich | | | |  |  |  |  |  |
| PT_46F28BFH_7316-1799 | | | | Ependymoma | | | | | | | 6091 | | | Immune Rich Tumor Recruitment | | | | | Immune Rich | | | |  |  |  |  |  |
| PT_48N4Y6YP_7316-1866 | | | | Low-grade glioma/astrocytoma (WHO grade I/II) | | | | | | | 4436 | | | Immune Stromal Rich | | | | | Immune Stromal Rich | | | |  |  |  |  |  |
| PT_4A2H0KNR_7316-2599 | | | | High-grade glioma/astrocytoma (WHO grade III/IV) | | | | | | | 1007 | | | Tumor Recruitment BMDM bias | | | | | Immune Rich | | | |  |  |  |  |  |
| PT_4ADZ9F53_7316-3060 | | | | Ganglioglioma | | | | | | | 4366 | | | Immune Rich | | | | | T cell Centric | | | |  |  |  |  |  |
| PT_4EDBZRM2_7316-1952 | | | | Ganglioglioma | | | | | | | 3940 | | | Immune Rich Tumor Rich | | | | | Immune Rich | | | |  |  |  |  |  |
| PT_4J24F353_7316-1137 | | | | Atypical Teratoid Rhabdoid Tumor (ATRT) | | | | | | | 575 | | | Immune Rich | | | | | Immune Rich | | | |  |  |  |  |  |
| PT_4JG5KWE8_7316-50 | | | | Low-grade glioma/astrocytoma (WHO grade I/II) | | | | | | | 3872 | | | Stromal Centered Myeloid bias | | | | | Myeloid stromal Centric | | | |  |  |  |  |  |
| PT_4RJ2EATN_7316-1653 | | | | Craniopharyngioma | | | | | | | 1351 | | | Immune Rich Tumor Recruitment | | | | | Immune Stromal Rich | | | |  |  |  |  |  |
| PT_4TASAQKA_7316-3566 | | | | Low-grade glioma/astrocytoma (WHO grade I/II) | | | | | | | 4386 | | | Immune Stromal Myeloid bias | | | | | Immune Stromal Rich | | | |  |  |  |  |  |
| PT_4XB3YTJM_7316-388 | | | | High-grade glioma/astrocytoma (WHO grade III/IV) | | | | | | | 1897 | | | Tumor Recruitment BMDM bias | | | | | Immune Stromal Rich | | | |  |  |  |  |  |
| PT_4XVA57J7_7316-1760 | | | | Low-grade glioma/astrocytoma (WHO grade I/II) | | | | | | | 3402 | | | Tumor Recruitment BMDM bias | | | | | Myeloid Centric | | | |  |  |  |  |  |
| PT_4YWZ538V_7316-719 | | | | Brainstem glioma- Diffuse intrinsic pontine glioma | | | | | | | 2955 | | | Immune Desert | | | | | Immune Desert | | | |  |  |  |  |  |
| PT_4Z34K4XB_7316-733 | | | | Low-grade glioma/astrocytoma (WHO grade I/II) | | | | | | | 3509 | | | Immune Rich Tumor Rich | | | | | Immune Rich | | | |  |  |  |  |  |
| PT_4ZSR5DY8_7316-264 | | | | Low-grade glioma/astrocytoma (WHO grade I/II) | | | | | | | 4640 | | | Immune Stromal Myeloid bias | | | | | Immune Rich | | | |  |  |  |  |  |
| PT_50ZFWMZE_7316-1942 | | | | Low-grade glioma/astrocytoma (WHO grade I/II) | | | | | | | 1570 | | | Immune Stromal Rich | | | | | Immune Stromal Rich | | | |  |  |  |  |  |
| PT_54MH6Q46_7316-2591 | | | | Low-grade glioma/astrocytoma (WHO grade I/II) | | | | | | | 1957 | | | Immune Rich | | | | | Myeloid Centric | | | |  |  |  |  |  |
| PT_59D00MBQ_7316-1936 | | | | High-grade glioma/astrocytoma (WHO grade III/IV) | | | | | | | 3283 | | | Stromal Centered Myeloid bias | | | | | Immune Stromal Desert | | | |  |  |  |  |  |
| PT_59D00MBQ_7316-1937 | | | | High-grade glioma/astrocytoma (WHO grade III/IV) | | | | | | | 3291 | | | Immune Stromal Rich | | | | | Immune Stromal Rich | | | |  |  |  |  |  |
| PT_59D00MBQ_7316-2810 | | | | High-grade glioma/astrocytoma (WHO grade III/IV) | | | | | | | 3702 | | | Tumor Rejected OG bias | | | | | Immune Stromal Desert | | | |  |  |  |  |  |
| PT_59FJRPVX_7316-3769 | | | | High-grade glioma/astrocytoma (WHO grade III/IV) | | | | | | | 3105 | | | Tumor Rejected OG bias | | | | | Immune Stromal Desert | | | |  |  |  |  |  |
| PT_5ABCFCSK_7316-1851 | | | | Low-grade glioma/astrocytoma (WHO grade I/II) | | | | | | | 5804 | | | Stromal Centered Myeloid bias | | | | | Myeloid Centric | | | |  |  |  |  |  |
| PT_5AF1501D_7316-309 | | | | Medulloblastoma | | | | | | | 4869 | | | Immune Desert Tumor Rejected | | | | | Immune Desert | | | |  |  |  |  |  |
| PT_5BWZA0NT_7316-1102 | | | | Supratentorial or Spinal Cord PNET | | | | | | | 4456 | | | Tumor Rejected OG bias | | | | | Immune Rich | | | |  |  |  |  |  |
| PT_5CKDR2SF_7316-525 | | | | Atypical Teratoid Rhabdoid Tumor (ATRT) | | | | | | | 394 | | | Tumor Rejected cDC2 bias | | | | | T cell Centric | | | |  |  |  |  |  |
| PT_5CNZ47XV_7316-1926 | | | | Low-grade glioma/astrocytoma (WHO grade I/II) | | | | | | | 5020 | | | Immune Stromal Rich | | | | | Immune Stromal Rich | | | |  |  |  |  |  |
| PT_5F4N0CAP_7316-219 | | | | Medulloblastoma | | | | | | | 3722 | | | Tumor Rejected OG bias | | | | | Immune Stromal Desert | | | |  |  |  |  |  |
| PT_5FR2YA6E_7316-109 | | | | Medulloblastoma | | | | | | | 4234 | | | Tumor Rejected OG bias | | | | | Immune Stromal Desert | | | |  |  |  |  |  |
| PT_5GQ76YZJ_7316-168 | | | | Low-grade glioma/astrocytoma (WHO grade I/II) | | | | | | | 1933 | | | Immune Stromal Rich | | | | | Immune Stromal Rich | | | |  |  |  |  |  |
| PT_5JA1N2H4_7316-839 | | | | Medulloblastoma | | | | | | | 1816 | | | Tumor Rejected OG bias | | | | | Immune Stromal Desert | | | |  |  |  |  |  |
| PT_5KRVE8E4_7316-1671 | | | | Craniopharyngioma | | | | | | | 4180 | | | Immune Rich | | | | | Immune Rich | | | |  |  |  |  |  |
| PT_5M8A47WW_7316-436 | | | | Low-grade glioma/astrocytoma (WHO grade I/II) | | | | | | | 603 | | | Immune Rich Tumor Rich | | | | | Immune Rich | | | |  |  |  |  |  |
| PT_5P3XJ97K_7316-390 | | | | Low-grade glioma/astrocytoma (WHO grade I/II) | | | | | | | 4667 | | | Immune Rich Tumor Rich | | | | | Immune Rich | | | |  |  |  |  |  |
| PT_5VAKED5F_7316-2689 | | | | Low-grade glioma/astrocytoma (WHO grade I/II) | | | | | | | 1607 | | | Immune Stromal Myeloid bias | | | | | Myeloid stromal Centric | | | |  |  |  |  |  |
| PT_625X34JX_7316-2170 | | | | Ependymoma | | | | | | | 6152 | | | Tumor Recruitment BMDM bias | | | | | Immune Desert | | | |  |  |  |  |  |
| PT_62VNQ8C4_7316-1884 | | | | Medulloblastoma | | | | | | | 5655 | | | Immune Desert | | | | | Immune Desert | | | |  |  |  |  |  |
| PT_62VNQ8C4_7316-968 | | | | Medulloblastoma | | | | | | | 5612 | | | Immune Rich | | | | | Immune Rich | | | |  |  |  |  |  |
| PT_63F63QY0_7316-3521 | | | | High-grade glioma/astrocytoma (WHO grade III/IV) | | | | | | | 5205 | | | Immune Rich Tumor Recruitment | | | | | Immune Rich | | | |  |  |  |  |  |
| PT_63HDZHCN_7316-2980 | | | | High-grade glioma/astrocytoma (WHO grade III/IV) | | | | | | | 2938 | | | Tumor Rejected cDC2 bias | | | | | Immune Stromal Desert | | | |  |  |  |  |  |
| PT_66XN3MT1_7316-936 | | | | Low-grade glioma/astrocytoma (WHO grade I/II) | | | | | | | 3902 | | | Immune Rich Tumor Recruitment | | | | | Immune Rich | | | |  |  |  |  |  |
| PT_67R1AF5R_7316-889 | | | | Ependymoma | | | | | | | 395 | | | Tumor Recruitment BMDM bias | | | | | Immune Desert | | | |  |  |  |  |  |
| PT_6BX46NVR_7316-3893 | | | | Ependymoma | | | | | | | 324 | | | Tumor Rejected OG bias | | | | | Immune Stromal Rich | | | |  |  |  |  |  |
| PT_6D9C46GC_7316-620 | | | | High-grade glioma/astrocytoma (WHO grade III/IV) | | | | | | | 192 | | | Tumor Recruitment BMDM bias | | | | | Immune Desert | | | |  |  |  |  |  |
| PT_6DW5NFKX_7316-3150 | | | | Low-grade glioma/astrocytoma (WHO grade I/II) | | | | | | | 4393 | | | Immune Rich Tumor Recruitment | | | | | Immune Rich | | | |  |  |  |  |  |
| PT_6E8JYRXM_7316-2729 | | | | Low-grade glioma/astrocytoma (WHO grade I/II) | | | | | | | 996 | | | Immune Rich Tumor Rich | | | | | Myeloid Centric | | | |  |  |  |  |  |
| PT_6GDPNFA6_7316-2610 | | | | High-grade glioma/astrocytoma (WHO grade III/IV) | | | | | | | 1820 | | | Tumor Rejected OG bias | | | | | Immune Stromal Desert | | | |  |  |  |  |  |
| PT_6MB6RED3_7316-226 | | | | Craniopharyngioma | | | | | | | 2800 | | | Immune Rich | | | | | Immune Rich | | | |  |  |  |  |  |
| PT_6MWPJ96F_7316-1711 | | | | Low-grade glioma/astrocytoma (WHO grade I/II) | | | | | | | 2889 | | | Immune Stromal Myeloid bias | | | | | Myeloid stromal Centric | | | |  |  |  |  |  |
| PT_6MWPJ96F_7316-1928 | | | | Low-grade glioma/astrocytoma (WHO grade I/II) | | | | | | | 2897 | | | Immune Stromal Myeloid bias | | | | | Myeloid stromal Centric | | | |  |  |  |  |  |
| PT_6N1QWCXE_7316-3169 | | | | Low-grade glioma/astrocytoma (WHO grade I/II) | | | | | | | 2869 | | | Immune Stromal Myeloid bias | | | | | Immune Stromal Rich | | | |  |  |  |  |  |
| PT_6Q0NPVP3_7316-2255 | | | | Ganglioglioma | | | | | | | 871 | | | Tumor Rejected cDC2 bias | | | | | Immune Stromal Desert | | | |  |  |  |  |  |
| PT_6R95FQT2_7316-459 | | | | Low-grade glioma/astrocytoma (WHO grade I/II) | | | | | | | 5262 | | | Immune Rich Tumor Rich | | | | | Immune Rich | | | |  |  |  |  |  |
| PT_6SH7PV1N_7316-535 | | | | High-grade glioma/astrocytoma (WHO grade III/IV) | | | | | | | 1405 | | | Tumor Rejected OG bias | | | | | Immune Stromal Desert | | | |  |  |  |  |  |
| PT_6TKJRSPW_7316-162 | | | | Low-grade glioma/astrocytoma (WHO grade I/II) | | | | | | | 6286 | | | Tumor Rejected OG bias | | | | | Myeloid stromal Centric | | | |  |  |  |  |  |
| PT_6TZR2DH1_7316-100 | | | | Craniopharyngioma | | | | | | | 2660 | | | Immune Rich | | | | | Immune Stromal Rich | | | |  |  |  |  |  |
| PT_6VJ5PNKS_7316-2174 | | | | Low-grade glioma/astrocytoma (WHO grade I/II) | | | | | | | 3979 | | | Stromal Centered Myeloid bias | | | | | Immune Stromal Desert | | | |  |  |  |  |  |
| PT_74XM7BV7_7316-312 | | | | Medulloblastoma | | | | | | | 1875 | | | Immune Desert | | | | | Immune Desert | | | |  |  |  |  |  |
| PT_7570EHD9_7316-1064 | | | | High-grade glioma/astrocytoma (WHO grade III/IV) | | | | | | | 2136 | | | Immune Desert Tumor Rejected | | | | | Immune Desert | | | |  |  |  |  |  |
| PT_76Q72M5T_7316-397 | | | | Low-grade glioma/astrocytoma (WHO grade I/II) | | | | | | | 4740 | | | Immune Rich | | | | | Immune Stromal Rich | | | |  |  |  |  |  |
| PT_78NQ0QQS_7316-1651 | | | | Medulloblastoma | | | | | | | 4406 | | | Tumor Rejected OG bias | | | | | Immune Stromal Desert | | | |  |  |  |  |  |
| PT_7BJBEBZT_7316-1670 | | | | Medulloblastoma | | | | | | | 2511 | | | Immune Desert Tumor Rejected | | | | | Immune Desert | | | |  |  |  |  |  |
| PT_7DTGJYA7_7316-1695 | | | | Brainstem glioma- Diffuse intrinsic pontine glioma | | | | | | | 2073 | | | Stromal Centered Myeloid bias | | | | | Immune Stromal Desert | | | |  |  |  |  |  |
| PT_7DTGJYA7_7316-639 | | | | Brainstem glioma- Diffuse intrinsic pontine glioma | | | | | | | 1070 | | | Stromal Centered Myeloid bias | | | | | Immune Stromal Desert | | | |  |  |  |  |  |
| PT_7E3V3JFX_7316-506 | | | | Ependymoma | | | | | | | 594 | | | Tumor Recruitment BMDM bias | | | | | Myeloid Centric | | | |  |  |  |  |  |
| PT_7F485CXV_7316-1114 | | | | Ganglioglioma | | | | | | | 308 | | | Tumor Rejected OG bias | | | | | Immune Stromal Desert | | | |  |  |  |  |  |
| PT_7FQ6VECA_7316-3846 | | | | Medulloblastoma | | | | | | | 5918 | | | Tumor Rejected OG bias | | | | | Immune Stromal Desert | | | |  |  |  |  |  |
| PT_7FZ8SE69_7316-2152 | | | | High-grade glioma/astrocytoma (WHO grade III/IV) | | | | | | | 4402 | | | Immune Rich | | | | | Immune Stromal Rich | | | |  |  |  |  |  |
| PT_7H67Q7VS_7316-2966 | | | | Ependymoma | | | | | | | 498 | | | Immune Desert | | | | | Immune Desert | | | |  |  |  |  |  |
| PT_7JQ24F35_7316-1681 | | | | Low-grade glioma/astrocytoma (WHO grade I/II) | | | | | | | 341 | | | Immune Rich Tumor Recruitment | | | | | Immune Rich | | | |  |  |  |  |  |
| PT_7JQ24F35_7316-1957 | | | | Low-grade glioma/astrocytoma (WHO grade I/II) | | | | | | | 492 | | | Immune Rich Tumor Recruitment | | | | | Immune Rich | | | |  |  |  |  |  |
| PT_7KBMN6C2_7316-553 | | | | Medulloblastoma | | | | | | | 2859 | | | Immune Desert Tumor Rejected | | | | | Immune Desert | | | |  |  |  |  |  |
| PT_7KTAH3RD_7316-2671 | | | | Craniopharyngioma | | | | | | | 2834 | | | Immune Rich | | | | | Immune Rich | | | |  |  |  |  |  |
| PT_7MZRW1DH_7316-2582 | | | | Atypical Teratoid Rhabdoid Tumor (ATRT) | | | | | | | 5688 | | | Immune Rich | | | | | Immune Stromal Rich | | | |  |  |  |  |  |
| PT_7PXN3B4J_7316-784 | | | | Supratentorial or Spinal Cord PNET | | | | | | | 690 | | | Tumor Rejected cDC2 bias | | | | | T cell Centric | | | |  |  |  |  |  |
| PT_7QKGGMKV_7316-347 | | | | Medulloblastoma | | | | | | | 3557 | | | Immune Desert Tumor Rejected | | | | | Immune Desert | | | |  |  |  |  |  |
| PT_7RMS6K08_7316-1748 | | | | Craniopharyngioma | | | | | | | 2033 | | | Immune Rich | | | | | Immune Rich | | | |  |  |  |  |  |
| PT_7T13FDWK_7316-1082 | | | | Craniopharyngioma | | | | | | | 4764 | | | Immune Rich | | | | | Immune Rich | | | |  |  |  |  |  |
| PT_7TEHW6VQ_7316-2755 | | | | Low-grade glioma/astrocytoma (WHO grade I/II) | | | | | | | 3998 | | | Stromal Centered Myeloid bias | | | | | Myeloid Centric | | | |  |  |  |  |  |
| PT_7TRGHZBK_7316-431 | | | | Choroid plexus carcinoma | | | | | | | 1098 | | | Immune Rich | | | | | Immune Stromal Rich | | | |  |  |  |  |  |
| PT_7WT6P5M8_7316-2308 | | | | High-grade glioma/astrocytoma (WHO grade III/IV) | | | | | | | 1422 | | | Stromal Centered Myeloid bias | | | | | Myeloid stromal Centric | | | |  |  |  |  |  |
| PT_7YGZ526E_7316-1978 | | | | Low-grade glioma/astrocytoma (WHO grade I/II) | | | | | | | 1168 | | | Immune Rich Tumor Rich | | | | | Immune Stromal Rich | | | |  |  |  |  |  |
| PT_7YSCM82T_7316-1658 | | | | Low-grade glioma/astrocytoma (WHO grade I/II) | | | | | | | 327 | | | Tumor Recruitment BMDM bias | | | | | Myeloid Centric | | | |  |  |  |  |  |
| PT_80NVYCBS_7316-765 | | | | Ependymoma | | | | | | | 5589 | | | Immune Desert | | | | | Immune Desert | | | |  |  |  |  |  |
| PT_82A9SDRN_7316-2313 | | | | Ependymoma | | | | | | | 4234 | | | Immune Desert | | | | | Myeloid Centric | | | |  |  |  |  |  |
| PT_82MX6J77_7316-302 | | | | Medulloblastoma | | | | | | | 1384 | | | Tumor Rejected cDC2 bias | | | | | Immune Desert | | | |  |  |  |  |  |
| PT_84Q11Z5X_7316-288 | | | | High-grade glioma/astrocytoma (WHO grade III/IV) | | | | | | | 5362 | | | Stromal Centered Myeloid bias | | | | | Immune Stromal Desert | | | |  |  |  |  |  |
| PT_85RSG67R_7316-2986 | | | | Medulloblastoma | | | | | | | 4068 | | | Immune Desert Tumor Rejected | | | | | Immune Desert | | | |  |  |  |  |  |
| PT_8ECE2D92_7316-914 | | | | Ganglioglioma | | | | | | | 3676 | | | Immune Rich Tumor Rich | | | | | Immune Rich | | | |  |  |  |  |  |
| PT_8FWP7BR3_7316-449 | | | | Low-grade glioma/astrocytoma (WHO grade I/II) | | | | | | | 4574 | | | Stromal Centered Myeloid bias | | | | | Immune Stromal Desert | | | |  |  |  |  |  |
| PT_8GBF5MTD_7316-166 | | | | Craniopharyngioma | | | | | | | 1985 | | | Immune Stromal Tumor Recruitment | | | | | Immune Stromal Rich | | | |  |  |  |  |  |
| PT_8GN3TQRM_7316-2069 | | | | High-grade glioma/astrocytoma (WHO grade III/IV) | | | | | | | 2515 | | | Immune Rich Tumor Recruitment | | | | | Immune Rich | | | |  |  |  |  |  |
| PT_8GN3TQRM_7316-2091 | | | | High-grade glioma/astrocytoma (WHO grade III/IV) | | | | | | | 2596 | | | Stromal Centered Myeloid bias | | | | | Myeloid stromal Centric | | | |  |  |  |  |  |
| PT_8GN3TQRM_7316-2737 | | | | High-grade glioma/astrocytoma (WHO grade III/IV) | | | | | | | 2842 | | | Tumor Recruitment BMDM bias | | | | | Immune Stromal Rich | | | |  |  |  |  |  |
| PT_8M6W215T_7316-305 | | | | Medulloblastoma | | | | | | | 6033 | | | Tumor Rejected cDC2 bias | | | | | T cell Centric | | | |  |  |  |  |  |
| PT_8PV84CNE_7316-532 | | | | Ependymoma | | | | | | | 2993 | | | Immune Desert | | | | | Myeloid Centric | | | |  |  |  |  |  |
| PT_8R00BMHH_7316-1845 | | | | Low-grade glioma/astrocytoma (WHO grade I/II) | | | | | | | 2595 | | | Immune Stromal Myeloid bias | | | | | Myeloid stromal Centric | | | |  |  |  |  |  |
| PT_8RB3EKNP_7316-4028 | | | | Medulloblastoma | | | | | | | 2059 | | | Tumor Rejected OG bias | | | | | Immune Stromal Desert | | | |  |  |  |  |  |
| PT_8RB7TPS2_7316-3 | | | | Ependymoma | | | | | | | 4199 | | | Tumor Rejected cDC2 bias | | | | | Immune Stromal Desert | | | |  |  |  |  |  |
| PT_8RB7TPS2_7316-322 | | | | Ependymoma | | | | | | | 4073 | | | Tumor Recruitment BMDM bias | | | | | Immune Desert | | | |  |  |  |  |  |
| PT_8SDJXNFR_7316-3755 | | | | Ependymoma | | | | | | | 1580 | | | Tumor Rejected OG bias | | | | | Immune Stromal Desert | | | |  |  |  |  |  |
| PT_8VRZRZ7R_7316-536 | | | | Supratentorial or Spinal Cord PNET | | | | | | | 3298 | | | Tumor Recruitment BMDM bias | | | | | Immune Desert | | | |  |  |  |  |  |
| PT_8XZY6MM1_7316-957 | | | | Low-grade glioma/astrocytoma (WHO grade I/II) | | | | | | | 2810 | | | Immune Stromal Myeloid bias | | | | | Immune Stromal Rich | | | |  |  |  |  |  |
| PT_90W4HCNR_7316-2672 | | | | Choroid plexus papilloma | | | | | | | 360 | | | Immune Desert | | | | | Immune Desert | | | |  |  |  |  |  |
| PT_94EHCMXA_7316-217 | | | | Low-grade glioma/astrocytoma (WHO grade I/II) | | | | | | | 1855 | | | Tumor Rejected OG bias | | | | | Immune Stromal Desert | | | |  |  |  |  |  |
| PT_962TCBVR_7316-2744 | | | | Low-grade glioma/astrocytoma (WHO grade I/II) | | | | | | | 2402 | | | Immune Rich Tumor Rich | | | | | Immune Rich | | | |  |  |  |  |  |
| PT_962TCBVR_7316-391 | | | | Low-grade glioma/astrocytoma (WHO grade I/II) | | | | | | | 1359 | | | Stromal Centered Myeloid bias | | | | | Myeloid Centric | | | |  |  |  |  |  |
| PT_962TCBVR_7316-896 | | | | Low-grade glioma/astrocytoma (WHO grade I/II) | | | | | | | 1717 | | | Immune Rich Tumor Rich | | | | | Immune Rich | | | |  |  |  |  |  |
| PT_98QMQZY7_7316-464 | | | | Supratentorial or Spinal Cord PNET | | | | | | | 1963 | | | Immune Desert | | | | | Immune Desert | | | |  |  |  |  |  |
| PT_9A6RPJ97_7316-906 | | | | Medulloblastoma | | | | | | | 2994 | | | Immune Desert Tumor Rejected | | | | | Immune Desert | | | |  |  |  |  |  |
| PT_9AHA6KW3_7316-395 | | | | Ganglioglioma | | | | | | | 2096 | | | Immune Stromal Tumor Recruitment | | | | | Immune Stromal Rich | | | |  |  |  |  |  |
| PT_9B6KWJT2_7316-1226 | | | | Low-grade glioma/astrocytoma (WHO grade I/II) | | | | | | | 4947 | | | Immune Stromal Rich | | | | | Immune Stromal Rich | | | |  |  |  |  |  |
| PT_9C2G356W_7316-3555 | | | | Low-grade glioma/astrocytoma (WHO grade I/II) | | | | | | | 1982 | | | Immune Rich Tumor Rich | | | | | Immune Rich | | | |  |  |  |  |  |
| PT_9CSZBV2P_7316-1473 | | | | Supratentorial or Spinal Cord PNET | | | | | | | 5157 | | | Immune Desert Tumor Rejected | | | | | Immune Desert | | | |  |  |  |  |  |
| PT_9D2N4S5Z_7316-93 | | | | Low-grade glioma/astrocytoma (WHO grade I/II) | | | | | | | 2738 | | | Tumor Rejected OG bias | | | | | Immune Stromal Desert | | | |  |  |  |  |  |
| PT_9DER14KH_7316-2230 | | | | Medulloblastoma | | | | | | | 3503 | | | Immune Desert | | | | | Immune Desert | | | |  |  |  |  |  |
| PT_9DMMZ08D_7316-1666 | | | | Medulloblastoma | | | | | | | 1433 | | | Immune Desert | | | | | Immune Desert | | | |  |  |  |  |  |
| PT_9DMMZ08D_7316-1676 | | | | Medulloblastoma | | | | | | | 1540 | | | Immune Desert | | | | | Immune Desert | | | |  |  |  |  |  |
| PT_9HMVHQEH_7316-22 | | | | Medulloblastoma | | | | | | | 2644 | | | Immune Desert Tumor Rejected | | | | | Immune Desert | | | |  |  |  |  |  |
| PT_9JT1441X_7316-544 | | | | Brainstem glioma- Diffuse intrinsic pontine glioma | | | | | | | 3989 | | | Tumor Recruitment BMDM bias | | | | | Immune Desert | | | |  |  |  |  |  |
| PT_9N0SR1D7_7316-249 | | | | High-grade glioma/astrocytoma (WHO grade III/IV) | | | | | | | 5418 | | | Tumor Rejected OG bias | | | | | Immune Stromal Desert | | | |  |  |  |  |  |
| PT_9S6WMQ92_7316-278 | | | | Medulloblastoma | | | | | | | 5534 | | | Tumor Rejected cDC2 bias | | | | | T cell Centric | | | |  |  |  |  |  |
| PT_9S6WMQ92_7316-95 | | | | Medulloblastoma | | | | | | | 6555 | | | Tumor Rejected OG bias | | | | | Immune Stromal Desert | | | |  |  |  |  |  |
| PT_9W4WTVFZ_7316-1312 | | | | High-grade glioma/astrocytoma (WHO grade III/IV) | | | | | | | 2870 | | | Immune Desert Tumor Rejected | | | | | Immune Desert | | | |  |  |  |  |  |
| PT_9YMRNZHH_7316-2178 | | | | Medulloblastoma | | | | | | | 5119 | | | Tumor Rejected OG bias | | | | | Immune Stromal Desert | | | |  |  |  |  |  |
| PT_A0F23R7E_7316-452 | | | | Ganglioglioma | | | | | | | 1464 | | | Tumor Rejected OG bias | | | | | T cell stromal Centric | | | |  |  |  |  |  |
| PT_A325EYR2_7316-2557 | | | | Brainstem glioma- Diffuse intrinsic pontine glioma | | | | | | | 2774 | | | Stromal Centered Myeloid bias | | | | | Immune Stromal Desert | | | |  |  |  |  |  |
| PT_A594A46X_7316-91 | | | | Ganglioglioma | | | | | | | 2507 | | | Tumor Rejected OG bias | | | | | Myeloid stromal Centric | | | |  |  |  |  |  |
| PT_A9F8Y6MA_7316-476 | | | | Low-grade glioma/astrocytoma (WHO grade I/II) | | | | | | | 199 | | | Immune Stromal Myeloid bias | | | | | Myeloid stromal Centric | | | |  |  |  |  |  |
| PT_ABV3RS3T_7316-471 | | | | Choroid plexus papilloma | | | | | | | 209 | | | Immune Desert | | | | | Immune Desert | | | |  |  |  |  |  |
| PT_AC5SD9AC_7316-2291 | | | | Low-grade glioma/astrocytoma (WHO grade I/II) | | | | | | | 1598 | | | Immune Stromal Myeloid bias | | | | | Immune Stromal Rich | | | |  |  |  |  |  |
| PT_ADTP7EHN_7316-1664 | | | | Low-grade glioma/astrocytoma (WHO grade I/II) | | | | | | | 2938 | | | Immune Stromal Myeloid bias | | | | | Immune Stromal Rich | | | |  |  |  |  |  |
| PT_AP658JQV_7316-1694 | | | | High-grade glioma/astrocytoma (WHO grade III/IV) | | | | | | | 5275 | | | Tumor Recruitment BMDM bias | | | | | Immune Stromal Desert | | | |  |  |  |  |  |
| PT_AP658JQV_7316-519 | | | | High-grade glioma/astrocytoma (WHO grade III/IV) | | | | | | | 4582 | | | Stromal Centered Myeloid bias | | | | | Immune Stromal Desert | | | |  |  |  |  |  |
| PT_ARCM3PXM_7316-1187 | | | | High-grade glioma/astrocytoma (WHO grade III/IV) | | | | | | | 4938 | | | Tumor Recruitment BMDM bias | | | | | Immune Desert | | | |  |  |  |  |  |
| PT_ATE3YRH5_7316-3308 | | | | Low-grade glioma/astrocytoma (WHO grade I/II) | | | | | | | 3106 | | | Immune Stromal Myeloid bias | | | | | Immune Rich | | | |  |  |  |  |  |
| PT_AWBPNFM5_7316-1854 | | | | Ganglioglioma | | | | | | | 5012 | | | Immune Stromal Myeloid bias | | | | | Immune Stromal Rich | | | |  |  |  |  |  |
| PT_AWMZ12NP_7316-2203 | | | | Ganglioglioma | | | | | | | 2514 | | | Immune Stromal Tumor Recruitment | | | | | Immune Stromal Rich | | | |  |  |  |  |  |
| PT_AWMZ12NP_7316-2216 | | | | Ganglioglioma | | | | | | | 2448 | | | Immune Stromal Tumor Recruitment | | | | | Immune Stromal Rich | | | |  |  |  |  |  |
| PT_AYVKQZRB_7316-612 | | | | Low-grade glioma/astrocytoma (WHO grade I/II) | | | | | | | 5247 | | | Immune Rich Tumor Recruitment | | | | | Immune Rich | | | |  |  |  |  |  |
| PT_AYZA2WCW_7316-197 | | | | Choroid plexus papilloma | | | | | | | 3206 | | | Immune Rich | | | | | Myeloid Centric | | | |  |  |  |  |  |
| PT_AZQ230WT_7316-443 | | | | Ependymoma | | | | | | | 371 | | | Immune Rich Tumor Recruitment | | | | | Immune Rich | | | |  |  |  |  |  |
| PT_B0JFCN5W_7316-1773 | | | | Craniopharyngioma | | | | | | | 4295 | | | Immune Stromal Tumor Recruitment | | | | | Immune Stromal Rich | | | |  |  |  |  |  |
| PT_B38P3Y5T_7316-3208 | | | | Brainstem glioma- Diffuse intrinsic pontine glioma | | | | | | | 3569 | | | Tumor Rejected OG bias | | | | | Immune Stromal Desert | | | |  |  |  |  |  |
| PT_B5K3A4F4_7316-1214 | | | | Ependymoma | | | | | | | 5573 | | | Tumor Recruitment BMDM bias | | | | | Immune Rich | | | |  |  |  |  |  |
| PT_B5VQ16GW_7316-488 | | | | Low-grade glioma/astrocytoma (WHO grade I/II) | | | | | | | 2687 | | | Immune Rich Tumor Rich | | | | | Immune Rich | | | |  |  |  |  |  |
| PT_B7QRTN72_7316-1802 | | | | Medulloblastoma | | | | | | | 1694 | | | Tumor Rejected cDC2 bias | | | | | T cell stromal Centric | | | |  |  |  |  |  |
| PT_BDNE55YT_7316-1710 | | | | Low-grade glioma/astrocytoma (WHO grade I/II) | | | | | | | 1472 | | | Immune Rich Tumor Rich | | | | | Immune Rich | | | |  |  |  |  |  |
| PT_BG7GAFK6_7316-3626 | | | | Medulloblastoma | | | | | | | 3385 | | | Tumor Rejected OG bias | | | | | Immune Stromal Desert | | | |  |  |  |  |  |
| PT_BK92NM09_7316-2669 | | | | Low-grade glioma/astrocytoma (WHO grade I/II) | | | | | | | 1357 | | | Immune Stromal Myeloid bias | | | | | Myeloid Centric | | | |  |  |  |  |  |
| PT_BNARR0N6_7316-1954 | | | | Ependymoma | | | | | | | 376 | | | Tumor Recruitment BMDM bias | | | | | Myeloid Centric | | | |  |  |  |  |  |
| PT_BPKVVWVB_7316-2250 | | | | Medulloblastoma | | | | | | | 3266 | | | Immune Desert Tumor Rejected | | | | | Immune Desert | | | |  |  |  |  |  |
| PT_BRTVGGRK_7316-2893 | | | | Medulloblastoma | | | | | | | 3424 | | | Immune Desert Tumor Rejected | | | | | Immune Desert | | | |  |  |  |  |  |
| PT_BTTM2HS5_7316-2287 | | | | Medulloblastoma | | | | | | | 1689 | | | Immune Desert | | | | | Immune Desert | | | |  |  |  |  |  |
| PT_BWGZBVD1_7316-1678 | | | | Low-grade glioma/astrocytoma (WHO grade I/II) | | | | | | | 315 | | | Immune Rich Tumor Recruitment | | | | | Immune Rich | | | |  |  |  |  |  |
| PT_BWN251DF_7316-659 | | | | Ependymoma | | | | | | | 6288 | | | Immune Desert | | | | | T cell Centric | | | |  |  |  |  |  |
| PT_BXXZE0CQ_7316-381 | | | | Low-grade glioma/astrocytoma (WHO grade I/II) | | | | | | | 3153 | | | Immune Stromal Rich | | | | | Immune Stromal Rich | | | |  |  |  |  |  |
| PT_BYJ428GA_7316-290 | | | | Low-grade glioma/astrocytoma (WHO grade I/II) | | | | | | | 2796 | | | Immune Rich Tumor Recruitment | | | | | Immune Rich | | | |  |  |  |  |  |
| PT_BZKJR4GK_7316-561 | | | | Craniopharyngioma | | | | | | | 6565 | | | Immune Rich | | | | | Immune Rich | | | |  |  |  |  |  |
| PT_C1M4YF1R_7316-127 | | | | Low-grade glioma/astrocytoma (WHO grade I/II) | | | | | | | 2857 | | | Immune Stromal Myeloid bias | | | | | Immune Rich | | | |  |  |  |  |  |
| PT_C2VY5NM6_7316-875 | | | | Low-grade glioma/astrocytoma (WHO grade I/II) | | | | | | | 735 | | | Tumor Rejected OG bias | | | | | T cell stromal Centric | | | |  |  |  |  |  |
| PT_C94VR1Y2_7316-2278 | | | | Ependymoma | | | | | | | 2814 | | | Tumor Recruitment BMDM bias | | | | | Immune Desert | | | |  |  |  |  |  |
| PT_C95KPXSW_7316-869 | | | | Low-grade glioma/astrocytoma (WHO grade I/II) | | | | | | | 4438 | | | Immune Stromal Myeloid bias | | | | | Immune Stromal Rich | | | |  |  |  |  |  |
| PT_CB1DN0V8_7316-2241 | | | | High-grade glioma/astrocytoma (WHO grade III/IV) | | | | | | | 2373 | | | Tumor Recruitment BMDM bias | | | | | Immune Desert | | | |  |  |  |  |  |
| PT_CB1XPA3W_7316-1767 | | | | Low-grade glioma/astrocytoma (WHO grade I/II) | | | | | | | 5083 | | | Immune Stromal Tumor Recruitment | | | | | Immune Stromal Rich | | | |  |  |  |  |  |
| PT_CB9PD1YP_7316-97 | | | | Low-grade glioma/astrocytoma (WHO grade I/II) | | | | | | | 6145 | | | Immune Stromal Myeloid bias | | | | | Immune Stromal Desert | | | |  |  |  |  |  |
| PT_CEWNZVKF_7316-124 | | | | Craniopharyngioma | | | | | | | 3725 | | | Tumor Rejected cDC2 bias | | | | | Immune Desert | | | |  |  |  |  |  |
| PT_CG2S2H2R_7316-974 | | | | Low-grade glioma/astrocytoma (WHO grade I/II) | | | | | | | 3007 | | | Immune Rich Tumor Recruitment | | | | | Immune Rich | | | |  |  |  |  |  |
| PT_CGPJY881_7316-891 | | | | Low-grade glioma/astrocytoma (WHO grade I/II) | | | | | | | 5213 | | | Immune Rich Tumor Rich | | | | | Immune Rich | | | |  |  |  |  |  |
| PT_CGWPWSXM_7316-2005 | | | | Ganglioglioma | | | | | | | 600 | | | Tumor Rejected OG bias | | | | | T cell stromal Centric | | | |  |  |  |  |  |
| PT_CK41DW03_7316-329 | | | | Low-grade glioma/astrocytoma (WHO grade I/II) | | | | | | | 4344 | | | Immune Rich Tumor Recruitment | | | | | Immune Rich | | | |  |  |  |  |  |
| PT_CM32ZCX6_7316-2215 | | | | Low-grade glioma/astrocytoma (WHO grade I/II) | | | | | | | 2548 | | | Stromal Centered Myeloid bias | | | | | Immune Stromal Rich | | | |  |  |  |  |  |
| PT_CMB6TASJ_7316-505 | | | | Supratentorial or Spinal Cord PNET | | | | | | | 772 | | | Immune Desert | | | | | Immune Desert | | | |  |  |  |  |  |
| PT_CQ7EGCMB_7316-3809 | | | | Medulloblastoma | | | | | | | 744 | | | Tumor Rejected cDC2 bias | | | | | Immune Desert | | | |  |  |  |  |  |
| PT_CR2YBSPT_7316-359 | | | | Ganglioglioma | | | | | | | 362 | | | Tumor Rejected OG bias | | | | | Immune Stromal Desert | | | |  |  |  |  |  |
| PT_CSZ9QA1N_7316-1464 | | | | High-grade glioma/astrocytoma (WHO grade III/IV) | | | | | | | 1886 | | | Immune Rich Tumor Rich | | | | | Immune Rich | | | |  |  |  |  |  |
| PT_CSZ9QA1N_7316-371 | | | | High-grade glioma/astrocytoma (WHO grade III/IV) | | | | | | | 3633 | | | Tumor Rejected OG bias | | | | | Immune Stromal Desert | | | |  |  |  |  |  |
| PT_CTSQ5FHH_7316-438 | | | | Medulloblastoma | | | | | | | 1663 | | | Immune Desert Tumor Rejected | | | | | Immune Desert | | | |  |  |  |  |  |
| PT_CW0BJE0Y_7316-41 | | | | Atypical Teratoid Rhabdoid Tumor (ATRT) | | | | | | | 883 | | | Immune Rich | | | | | Immune Rich | | | |  |  |  |  |  |
| PT_CWA1405H_7316-2252 | | | | Medulloblastoma | | | | | | | 3475 | | | Immune Desert Tumor Rejected | | | | | Immune Desert | | | |  |  |  |  |  |
| PT_CWD717Q0_7316-101 | | | | Ganglioglioma | | | | | | | 3472 | | | Tumor Rejected OG bias | | | | | Immune Stromal Desert | | | |  |  |  |  |  |
| PT_CXT81GRM_7316-1772 | | | | Medulloblastoma | | | | | | | 1545 | | | Tumor Rejected OG bias | | | | | Immune Stromal Desert | | | |  |  |  |  |  |
| PT_CYBYN18G_7316-2567 | | | | Ependymoma | | | | | | | 3474 | | | Tumor Rejected cDC2 bias | | | | | Immune Desert | | | |  |  |  |  |  |
| PT_CYVVA9AB_7316-3299 | | | | Ependymoma | | | | | | | 4738 | | | Tumor Recruitment BMDM bias | | | | | Myeloid Centric | | | |  |  |  |  |  |
| PT_CZRDBM1Q_7316-435 | | | | Medulloblastoma | | | | | | | 2907 | | | Immune Desert Tumor Rejected | | | | | Immune Desert | | | |  |  |  |  |  |
| PT_D1WJ3BJX_7316-358 | | | | Medulloblastoma | | | | | | | 5845 | | | Tumor Rejected cDC2 bias | | | | | Immune Desert | | | |  |  |  |  |  |
| PT_D215EQAP_7316-747 | | | | Medulloblastoma | | | | | | | 2759 | | | Immune Desert Tumor Rejected | | | | | Immune Desert | | | |  |  |  |  |  |
| PT_D3AN5M5W_7316-47 | | | | Ependymoma | | | | | | | 4337 | | | Tumor Recruitment BMDM bias | | | | | Immune Desert | | | |  |  |  |  |  |
| PT_D5KHK0SS_7316-882 | | | | Low-grade glioma/astrocytoma (WHO grade I/II) | | | | | | | 6552 | | | Immune Stromal Tumor Recruitment | | | | | Immune Stromal Rich | | | |  |  |  |  |  |
| PT_D7KMPYTZ_7316-2133 | | | | Medulloblastoma | | | | | | | 4729 | | | Immune Desert | | | | | Immune Desert | | | |  |  |  |  |  |
| PT_DACV00WF_7316-489 | | | | Medulloblastoma | | | | | | | 116 | | | Immune Desert Tumor Rejected | | | | | Immune Desert | | | |  |  |  |  |  |
| PT_DB800CE1_7316-261 | | | | Ganglioglioma | | | | | | | 2093 | | | Immune Stromal Rich | | | | | Immune Stromal Rich | | | |  |  |  |  |  |
| PT_DDFAZ345_7316-321 | | | | Ganglioglioma | | | | | | | 1441 | | | Tumor Rejected OG bias | | | | | Immune Stromal Desert | | | |  |  |  |  |  |
| PT_DESHZ91V_7316-2009 | | | | Medulloblastoma | | | | | | | 5110 | | | Tumor Rejected cDC2 bias | | | | | Immune Desert | | | |  |  |  |  |  |
| PT_DEYCW197_7316-175 | | | | Low-grade glioma/astrocytoma (WHO grade I/II) | | | | | | | 3917 | | | Immune Stromal Tumor Recruitment | | | | | Immune Stromal Rich | | | |  |  |  |  |  |
| PT_DFQAH7RS_7316-2760 | | | | Ganglioglioma | | | | | | | 1645 | | | Tumor Rejected OG bias | | | | | Immune Stromal Desert | | | |  |  |  |  |  |
| PT_DFXQFYPV_7316-2155 | | | | Medulloblastoma | | | | | | | 3606 | | | Tumor Rejected cDC2 bias | | | | | Immune Desert | | | |  |  |  |  |  |
| PT_DJ106XYS_7316-3935 | | | | High-grade glioma/astrocytoma (WHO grade III/IV) | | | | | | | 4489 | | | Immune Rich Tumor Recruitment | | | | | Immune Rich | | | |  |  |  |  |  |
| PT_DMHFS0HX_7316-3055 | | | | Low-grade glioma/astrocytoma (WHO grade I/II) | | | | | | | 3765 | | | Immune Stromal Rich | | | | | Immune Stromal Rich | | | |  |  |  |  |  |
| PT_DMMKT7X1_7316-1635 | | | | Low-grade glioma/astrocytoma (WHO grade I/II) | | | | | | | 5113 | | | Immune Rich Tumor Rich | | | | | Immune Rich | | | |  |  |  |  |  |
| PT_DNAJYFZT_7316-4035 | | | | Ependymoma | | | | | | | 5083 | | | Stromal Centered Myeloid bias | | | | | Immune Stromal Desert | | | |  |  |  |  |  |
| PT_DNH1VP67_7316-1062 | | | | High-grade glioma/astrocytoma (WHO grade III/IV) | | | | | | | 2224 | | | Tumor Recruitment BMDM bias | | | | | Immune Stromal Desert | | | |  |  |  |  |  |
| PT_DQ3ZCHB6_7316-2195 | | | | Low-grade glioma/astrocytoma (WHO grade I/II) | | | | | | | 809 | | | Immune Stromal Myeloid bias | | | | | T cell stromal Centric | | | |  |  |  |  |  |
| PT_DW2XRPC9_7316-430 | | | | High-grade glioma/astrocytoma (WHO grade III/IV) | | | | | | | 4384 | | | Immune Rich | | | | | Immune Rich | | | |  |  |  |  |  |
| PT_DW6V5V8E_7316-924 | | | | Low-grade glioma/astrocytoma (WHO grade I/II) | | | | | | | 1069 | | | Immune Rich Tumor Rich | | | | | Immune Rich | | | |  |  |  |  |  |
| PT_DXF7ZZQZ_7316-2809 | | | | Ganglioglioma | | | | | | | 1559 | | | Tumor Rejected OG bias | | | | | Myeloid stromal Centric | | | |  |  |  |  |  |
| PT_E1TRJP8V_7316-134 | | | | Low-grade glioma/astrocytoma (WHO grade I/II) | | | | | | | 2419 | | | Immune Rich Tumor Recruitment | | | | | Immune Rich | | | |  |  |  |  |  |
| PT_E3ADF4ZB_7316-2664 | | | | Low-grade glioma/astrocytoma (WHO grade I/II) | | | | | | | 1352 | | | Immune Rich Tumor Recruitment | | | | | Immune Rich | | | |  |  |  |  |  |
| PT_E3S6H59E_7316-133 | | | | Oligodendroglioma | | | | | | | 4921 | | | Tumor Rejected OG bias | | | | | T cell stromal Centric | | | |  |  |  |  |  |
| PT_E3Y5BBSF_7316-3071 | | | | Craniopharyngioma | | | | | | | 1709 | | | Immune Rich | | | | | Immune Rich | | | |  |  |  |  |  |
| PT_E4ECAFEN_7316-38 | | | | Medulloblastoma | | | | | | | 5666 | | | Tumor Rejected cDC2 bias | | | | | Immune Desert | | | |  |  |  |  |  |
| PT_E6FTW238_7316-3890 | | | | Atypical Teratoid Rhabdoid Tumor (ATRT) | | | | | | | 557 | | | Tumor Rejected cDC2 bias | | | | | T cell Centric | | | |  |  |  |  |  |
| PT_E78NHRWQ_7316-1081 | | | | Low-grade glioma/astrocytoma (WHO grade I/II) | | | | | | | 1825 | | | Immune Rich Tumor Rich | | | | | Immune Rich | | | |  |  |  |  |  |
| PT_E79807KT_7316-1087 | | | | Low-grade glioma/astrocytoma (WHO grade I/II) | | | | | | | 2809 | | | Immune Rich Tumor Recruitment | | | | | Immune Rich | | | |  |  |  |  |  |
| PT_E8XM8JFZ_7316-324 | | | | Ganglioglioma | | | | | | | 1302 | | | Immune Rich Tumor Rich | | | | | Myeloid Centric | | | |  |  |  |  |  |
| PT_EB89G70N_7316-2858 | | | | Ependymoma | | | | | | | 4822 | | | Immune Rich Tumor Recruitment | | | | | Immune Rich | | | |  |  |  |  |  |
| PT_EC8R84NM_7316-341 | | | | Craniopharyngioma | | | | | | | 1344 | | | Immune Rich | | | | | Immune Stromal Rich | | | |  |  |  |  |  |
| PT_ECMZCNTF_7316-736 | | | | Ependymoma | | | | | | | 479 | | | Immune Rich | | | | | Immune Rich | | | |  |  |  |  |  |
| PT_EDWA0XTP_7316-670 | | | | Medulloblastoma | | | | | | | 1032 | | | Tumor Rejected cDC2 bias | | | | | T cell Centric | | | |  |  |  |  |  |
| PT_EEKE0V9T_7316-32 | | | | Low-grade glioma/astrocytoma (WHO grade I/II) | | | | | | | 6259 | | | Immune Stromal Tumor Recruitment | | | | | Immune Stromal Rich | | | |  |  |  |  |  |
| PT_EF398HVC_7316-1975 | | | | Low-grade glioma/astrocytoma (WHO grade I/II) | | | | | | | 2252 | | | Immune Rich Tumor Rich | | | | | Immune Rich | | | |  |  |  |  |  |
| PT_EG7PH32E_7316-2144 | | | | Ependymoma | | | | | | | 5028 | | | Tumor Rejected cDC2 bias | | | | | Immune Stromal Desert | | | |  |  |  |  |  |
| PT_EGGY1XC0_7316-2865 | | | | Medulloblastoma | | | | | | | 1475 | | | Immune Desert Tumor Rejected | | | | | Immune Desert | | | |  |  |  |  |  |
| PT_EHE800JJ_7316-229 | | | | Craniopharyngioma | | | | | | | 1738 | | | Immune Rich | | | | | T cell Centric | | | |  |  |  |  |  |
| PT_EJ8TKG2Z_7316-3571 | | | | Craniopharyngioma | | | | | | | 3511 | | | Immune Rich | | | | | Immune Rich | | | |  |  |  |  |  |
| PT_EQ5C5TEA_7316-3922 | | | | Low-grade glioma/astrocytoma (WHO grade I/II) | | | | | | | 4043 | | | Immune Stromal Rich | | | | | Immune Stromal Rich | | | |  |  |  |  |  |
| PT_ESHACWF6_7316-1790 | | | | Atypical Teratoid Rhabdoid Tumor (ATRT) | | | | | | | 1904 | | | Tumor Recruitment BMDM bias | | | | | Immune Desert | | | |  |  |  |  |  |
| PT_ESHACWF6_7316-878 | | | | Atypical Teratoid Rhabdoid Tumor (ATRT) | | | | | | | 1506 | | | Tumor Recruitment BMDM bias | | | | | Immune Desert | | | |  |  |  |  |  |
| PT_EW604JB3_7316-1134 | | | | Low-grade glioma/astrocytoma (WHO grade I/II) | | | | | | | 1244 | | | Immune Stromal Myeloid bias | | | | | Myeloid stromal Centric | | | |  |  |  |  |  |
| PT_EXYXBDGP_7316-3140 | | | | Low-grade glioma/astrocytoma (WHO grade I/II) | | | | | | | 6045 | | | Immune Stromal Rich | | | | | Immune Stromal Rich | | | |  |  |  |  |  |
| PT_EYHJQQ8E_7316-77 | | | | Low-grade glioma/astrocytoma (WHO grade I/II) | | | | | | | 4457 | | | Stromal Centered Myeloid bias | | | | | Immune Stromal Desert | | | |  |  |  |  |  |
| PT_EYZ25B99_7316-470 | | | | Low-grade glioma/astrocytoma (WHO grade I/II) | | | | | | | 5738 | | | Tumor Rejected OG bias | | | | | Immune Stromal Desert | | | |  |  |  |  |  |
| PT_EZEMVRVT_7316-3154 | | | | Low-grade glioma/astrocytoma (WHO grade I/II) | | | | | | | 1171 | | | Immune Rich Tumor Rich | | | | | Immune Rich | | | |  |  |  |  |  |
| PT_EZS4SRH6_7316-676 | | | | Medulloblastoma | | | | | | | 4044 | | | Tumor Rejected OG bias | | | | | Immune Stromal Desert | | | |  |  |  |  |  |
| PT_F0N89D15_7316-3756 | | | | Medulloblastoma | | | | | | | 2031 | | | Immune Desert Tumor Rejected | | | | | Immune Desert | | | |  |  |  |  |  |
| PT_F27SA15G_7316-1661 | | | | Low-grade glioma/astrocytoma (WHO grade I/II) | | | | | | | 4641 | | | Immune Rich Tumor Rich | | | | | Immune Rich | | | |  |  |  |  |  |
| PT_F3S9PB94_7316-2240 | | | | Medulloblastoma | | | | | | | 3049 | | | Immune Desert Tumor Rejected | | | | | Immune Desert | | | |  |  |  |  |  |
| PT_F9G665B1_7316-1886 | | | | Ependymoma | | | | | | | 660 | | | Immune Rich Tumor Recruitment | | | | | Immune Rich | | | |  |  |  |  |  |
| PT_FAD8FQ6S_7316-120 | | | | Ganglioglioma | | | | | | | 6287 | | | Stromal Centered Myeloid bias | | | | | Immune Stromal Rich | | | |  |  |  |  |  |
| PT_FAPEH6R1_7316-1660 | | | | Low-grade glioma/astrocytoma (WHO grade I/II) | | | | | | | 4379 | | | Stromal Centered Myeloid bias | | | | | Immune Stromal Desert | | | |  |  |  |  |  |
| PT_FAQWD7GX_7316-2311 | | | | Craniopharyngioma | | | | | | | 2195 | | | Immune Stromal Tumor Recruitment | | | | | Immune Stromal Rich | | | |  |  |  |  |  |
| PT_FC0EF5ZT_7316-3572 | | | | Ganglioglioma | | | | | | | 4482 | | | Tumor Rejected OG bias | | | | | Myeloid stromal Centric | | | |  |  |  |  |  |
| PT_FCM7XPVR_7316-2285 | | | | Low-grade glioma/astrocytoma (WHO grade I/II) | | | | | | | 6440 | | | Tumor Rejected OG bias | | | | | Immune Stromal Rich | | | |  |  |  |  |  |
| PT_FF0MSK4D_7316-2987 | | | | Supratentorial or Spinal Cord PNET | | | | | | | 6082 | | | Immune Desert | | | | | Immune Desert | | | |  |  |  |  |  |
| PT_FF0NV7ZM_7316-716 | | | | High-grade glioma/astrocytoma (WHO grade III/IV) | | | | | | | 537 | | | Tumor Recruitment BMDM bias | | | | | Immune Desert | | | |  |  |  |  |  |
| PT_FFRHWB74_7316-932 | | | | Low-grade glioma/astrocytoma (WHO grade I/II) | | | | | | | 4774 | | | Immune Rich Tumor Rich | | | | | Immune Rich | | | |  |  |  |  |  |
| PT_FHDZNN40_7316-405 | | | | Ependymoma | | | | | | | 130 | | | Tumor Recruitment BMDM bias | | | | | Immune Rich | | | |  |  |  |  |  |
| PT_FKWCVXNC_7316-332 | | | | Low-grade glioma/astrocytoma (WHO grade I/II) | | | | | | | 1178 | | | Immune Stromal Myeloid bias | | | | | Myeloid stromal Centric | | | |  |  |  |  |  |
| PT_FMK5VFYW_7316-950 | | | | Supratentorial or Spinal Cord PNET | | | | | | | 374 | | | Immune Desert Tumor Rejected | | | | | Immune Desert | | | |  |  |  |  |  |
| PT_FN4GEEFR_7316-3010 | | | | Medulloblastoma | | | | | | | 5041 | | | Immune Desert Tumor Rejected | | | | | Immune Desert | | | |  |  |  |  |  |
| PT_FN4GEEFR_7316-729 | | | | Medulloblastoma | | | | | | | 3977 | | | Immune Desert Tumor Rejected | | | | | Immune Desert | | | |  |  |  |  |  |
| PT_FWD7NZQ0_7316-1108 | | | | Ganglioglioma | | | | | | | 5179 | | | Immune Stromal Rich | | | | | Immune Stromal Rich | | | |  |  |  |  |  |
| PT_FXPJM0MW_7316-207 | | | | Low-grade glioma/astrocytoma (WHO grade I/II) | | | | | | | 1857 | | | Immune Stromal Rich | | | | | Immune Stromal Rich | | | |  |  |  |  |  |
| PT_FYECBRPP_7316-1778 | | | | Atypical Teratoid Rhabdoid Tumor (ATRT) | | | | | | | 562 | | | Tumor Rejected OG bias | | | | | Immune Stromal Desert | | | |  |  |  |  |  |
| PT_FYPWZBH0_7316-1085 | | | | High-grade glioma/astrocytoma (WHO grade III/IV) | | | | | | | 3360 | | | Immune Desert Tumor Rejected | | | | | Immune Desert | | | |  |  |  |  |  |
| PT_FZP24XYK_7316-1060 | | | | High-grade glioma/astrocytoma (WHO grade III/IV) | | | | | | | 6006 | | | Immune Rich | | | | | Immune Stromal Rich | | | |  |  |  |  |  |
| PT_G1QNGVCZ_7316-1118 | | | | Medulloblastoma | | | | | | | 3712 | | | Stromal Centered Myeloid bias | | | | | Immune Stromal Desert | | | |  |  |  |  |  |
| PT_G3AVDN4E_7316-2227 | | | | Medulloblastoma | | | | | | | 2320 | | | Immune Desert Tumor Rejected | | | | | Immune Desert | | | |  |  |  |  |  |
| PT_G4N67N0D_7316-1057 | | | | High-grade glioma/astrocytoma (WHO grade III/IV) | | | | | | | 4550 | | | Stromal Centered Myeloid bias | | | | | Immune Stromal Desert | | | |  |  |  |  |  |
| PT_GAM3E6M3_7316-872 | | | | Ependymoma | | | | | | | 2365 | | | Immune Stromal Tumor Recruitment | | | | | Immune Stromal Rich | | | |  |  |  |  |  |
| PT_GCKT4FJX_7316-3919 | | | | Low-grade glioma/astrocytoma (WHO grade I/II) | | | | | | | 3242 | | | Immune Rich Tumor Rich | | | | | Immune Rich | | | |  |  |  |  |  |
| PT_GFTCNWQ0_7316-775 | | | | Ependymoma | | | | | | | 3907 | | | Immune Desert | | | | | Myeloid Centric | | | |  |  |  |  |  |
| PT_GGCQAGPS_7316-304 | | | | Medulloblastoma | | | | | | | 2265 | | | Immune Desert Tumor Rejected | | | | | Immune Desert | | | |  |  |  |  |  |
| PT_GH27VJBD_7316-2266 | | | | Ependymoma | | | | | | | 2471 | | | Tumor Rejected OG bias | | | | | Immune Stromal Desert | | | |  |  |  |  |  |
| PT_GH693TT8_7316-2181 | | | | Atypical Teratoid Rhabdoid Tumor (ATRT) | | | | | | | 738 | | | Tumor Rejected cDC2 bias | | | | | Immune Rich | | | |  |  |  |  |  |
| PT_GH6HM5QF_7316-3202 | | | | Medulloblastoma | | | | | | | 526 | | | Tumor Rejected cDC2 bias | | | | | T cell Centric | | | |  |  |  |  |  |
| PT_GHHY5AAE_7316-741 | | | | Low-grade glioma/astrocytoma (WHO grade I/II) | | | | | | | 5054 | | | Immune Stromal Myeloid bias | | | | | Myeloid stromal Centric | | | |  |  |  |  |  |
| PT_GKSABESN_7316-3485 | | | | Choroid plexus papilloma | | | | | | | 108 | | | Immune Desert | | | | | Immune Desert | | | |  |  |  |  |  |
| PT_GKWWWJ0X_7316-1071 | | | | Ganglioglioma | | | | | | | 432 | | | Immune Stromal Rich | | | | | Immune Stromal Rich | | | |  |  |  |  |  |
| PT_GQDHZFJP_7316-265 | | | | Low-grade glioma/astrocytoma (WHO grade I/II) | | | | | | | 5654 | | | Immune Rich Tumor Recruitment | | | | | Immune Rich | | | |  |  |  |  |  |
| PT_GQZ84ACS_7316-487 | | | | Low-grade glioma/astrocytoma (WHO grade I/II) | | | | | | | 560 | | | Stromal Centered Myeloid bias | | | | | Myeloid stromal Centric | | | |  |  |  |  |  |
| PT_GY0QSY4H_7316-1075 | | | | Craniopharyngioma | | | | | | | 4406 | | | Immune Rich | | | | | Immune Rich | | | |  |  |  |  |  |
| PT_GY99ZJHV_7316-1654 | | | | Choroid plexus papilloma | | | | | | | 670 | | | Tumor Rejected OG bias | | | | | Myeloid stromal Centric | | | |  |  |  |  |  |
| PT_H0HWHRPB_7316-1679 | | | | Low-grade glioma/astrocytoma (WHO grade I/II) | | | | | | | 3379 | | | Tumor Rejected OG bias | | | | | Immune Stromal Desert | | | |  |  |  |  |  |
| PT_H2HT2WQX_7316-619 | | | | High-grade glioma/astrocytoma (WHO grade III/IV) | | | | | | | 1292 | | | Immune Rich Tumor Rich | | | | | Immune Rich | | | |  |  |  |  |  |
| PT_H3WWDMW9_7316-2118 | | | | Medulloblastoma | | | | | | | 486 | | | Immune Desert Tumor Rejected | | | | | Immune Desert | | | |  |  |  |  |  |
| PT_H3WWDMW9_7316-737 | | | | Medulloblastoma | | | | | | | 222 | | | Immune Desert Tumor Rejected | | | | | Immune Desert | | | |  |  |  |  |  |
| PT_H45M7M2T_7316-1652 | | | | Low-grade glioma/astrocytoma (WHO grade I/II) | | | | | | | 4420 | | | Immune Stromal Tumor Recruitment | | | | | Immune Stromal Rich | | | |  |  |  |  |  |
| PT_H45M7M2T_7316-3074 | | | | Low-grade glioma/astrocytoma (WHO grade I/II) | | | | | | | 4532 | | | Immune Stromal Myeloid bias | | | | | Immune Stromal Rich | | | |  |  |  |  |  |
| PT_H57TEQDT_7316-2324 | | | | Low-grade glioma/astrocytoma (WHO grade I/II) | | | | | | | 1227 | | | Immune Stromal Myeloid bias | | | | | Myeloid Centric | | | |  |  |  |  |  |
| PT_H6E09982_7316-5 | | | | Medulloblastoma | | | | | | | 2857 | | | Tumor Rejected OG bias | | | | | Immune Stromal Desert | | | |  |  |  |  |  |
| PT_H876VB6R_7316-1806 | | | | Ependymoma | | | | | | | 372 | | | Tumor Recruitment BMDM bias | | | | | Myeloid Centric | | | |  |  |  |  |  |
| PT_H8Z52K09_7316-2224 | | | | Atypical Teratoid Rhabdoid Tumor (ATRT) | | | | | | | 624 | | | Tumor Recruitment BMDM bias | | | | | Immune Desert | | | |  |  |  |  |  |
| PT_HEGTYPQJ_7316-2562 | | | | Medulloblastoma | | | | | | | 4850 | | | Immune Desert Tumor Rejected | | | | | T cell Centric | | | |  |  |  |  |  |
| PT_HGHV85PZ_7316-2300 | | | | Ganglioglioma | | | | | | | 5209 | | | Stromal Centered Myeloid bias | | | | | Immune Desert | | | |  |  |  |  |  |
| PT_HKWE1GCK_7316-259 | | | | Ganglioglioma | | | | | | | 472 | | | Immune Rich Tumor Recruitment | | | | | Immune Rich | | | |  |  |  |  |  |
| PT_HM2GMT2H_7316-6 | | | | Medulloblastoma | | | | | | | 604 | | | Immune Desert Tumor Rejected | | | | | Immune Desert | | | |  |  |  |  |  |
| PT_HMF8J4EG_7316-644 | | | | High-grade glioma/astrocytoma (WHO grade III/IV) | | | | | | | 2879 | | | Tumor Recruitment BMDM bias | | | | | Myeloid Centric | | | |  |  |  |  |  |
| PT_HMY997GX_7316-3554 | | | | Low-grade glioma/astrocytoma (WHO grade I/II) | | | | | | | 2234 | | | Tumor Recruitment BMDM bias | | | | | Myeloid Centric | | | |  |  |  |  |  |
| PT_HV6C0T34_7316-3062 | | | | Low-grade glioma/astrocytoma (WHO grade I/II) | | | | | | | 1612 | | | Stromal Centered Myeloid bias | | | | | Immune Stromal Rich | | | |  |  |  |  |  |
| PT_HVZTF42R_7316-2187 | | | | Atypical Teratoid Rhabdoid Tumor (ATRT) | | | | | | | 170 | | | Immune Rich Tumor Recruitment | | | | | Immune Rich | | | |  |  |  |  |  |
| PT_HYXSSCJ5_7316-1088 | | | | Low-grade glioma/astrocytoma (WHO grade I/II) | | | | | | | 2522 | | | Tumor Rejected OG bias | | | | | Immune Stromal Desert | | | |  |  |  |  |  |
| PT_J0SZ1SX5_7316-2589 | | | | Low-grade glioma/astrocytoma (WHO grade I/II) | | | | | | | 210 | | | Immune Rich | | | | | Myeloid stromal Centric | | | |  |  |  |  |  |
| PT_J302B8Y5_7316-386 | | | | Atypical Teratoid Rhabdoid Tumor (ATRT) | | | | | | | 826 | | | Immune Desert | | | | | Immune Desert | | | |  |  |  |  |  |
| PT_JC4DHFJR_7316-2536 | | | | High-grade glioma/astrocytoma (WHO grade III/IV) | | | | | | | 4656 | | | Immune Desert | | | | | Immune Desert | | | |  |  |  |  |  |
| PT_JCECCFPB_7316-479 | | | | Atypical Teratoid Rhabdoid Tumor (ATRT) | | | | | | | 398 | | | Tumor Rejected OG bias | | | | | Immune Stromal Desert | | | |  |  |  |  |  |
| PT_JEA0AEMZ_7316-2169 | | | | Medulloblastoma | | | | | | | 2327 | | | Immune Desert Tumor Rejected | | | | | Immune Desert | | | |  |  |  |  |  |
| PT_JJ8QNAM8_7316-714 | | | | High-grade glioma/astrocytoma (WHO grade III/IV) | | | | | | | 4493 | | | Immune Desert Tumor Rejected | | | | | Immune Desert | | | |  |  |  |  |  |
| PT_JMW6Q6NH_7316-1648 | | | | Low-grade glioma/astrocytoma (WHO grade I/II) | | | | | | | 432 | | | Immune Rich Tumor Rich | | | | | Immune Rich | | | |  |  |  |  |  |
| PT_JN3EN2HR_7316-2267 | | | | Ependymoma | | | | | | | 3887 | | | Tumor Recruitment BMDM bias | | | | | Immune Rich | | | |  |  |  |  |  |
| PT_JNEV57VK_7316-2594 | | | | High-grade glioma/astrocytoma (WHO grade III/IV) | | | | | | | 2869 | | | Immune Rich Tumor Rich | | | | | Immune Rich | | | |  |  |  |  |  |
| PT_JP1FDKN9_7316-2959 | | | | Ependymoma | | | | | | | 3143 | | | Immune Rich | | | | | Immune Rich | | | |  |  |  |  |  |
| PT_JQ45JK94_7316-222 | | | | Low-grade glioma/astrocytoma (WHO grade I/II) | | | | | | | 2750 | | | Tumor Recruitment BMDM bias | | | | | Myeloid Centric | | | |  |  |  |  |  |
| PT_JSFBMK5V_7316-392 | | | | High-grade glioma/astrocytoma (WHO grade III/IV) | | | | | | | 5653 | | | Stromal Centered Myeloid bias | | | | | Immune Stromal Rich | | | |  |  |  |  |  |
| PT_JT9HH7M6_7316-1455 | | | | High-grade glioma/astrocytoma (WHO grade III/IV) | | | | | | | 636 | | | Immune Rich | | | | | Immune Rich | | | |  |  |  |  |  |
| PT_JV9G8MFD_7316-2269 | | | | Atypical Teratoid Rhabdoid Tumor (ATRT) | | | | | | | 362 | | | Tumor Recruitment BMDM bias | | | | | Immune Desert | | | |  |  |  |  |  |
| PT_JWW33RTB_7316-1793 | | | | Ganglioglioma | | | | | | | 4186 | | | Immune Rich | | | | | Immune Stromal Rich | | | |  |  |  |  |  |
| PT_JXGMAHHN_7316-874 | | | | Low-grade glioma/astrocytoma (WHO grade I/II) | | | | | | | 796 | | | Immune Stromal Myeloid bias | | | | | Immune Stromal Rich | | | |  |  |  |  |  |
| PT_JYZA0PNV_7316-437 | | | | Low-grade glioma/astrocytoma (WHO grade I/II) | | | | | | | 2321 | | | Immune Stromal Myeloid bias | | | | | T cell Centric | | | |  |  |  |  |  |
| PT_K2YDNZDA_7316-429 | | | | Ependymoma | | | | | | | 809 | | | Immune Desert | | | | | Immune Desert | | | |  |  |  |  |  |
| PT_K30AWQ8A_7316-393 | | | | Low-grade glioma/astrocytoma (WHO grade I/II) | | | | | | | 4087 | | | Immune Stromal Myeloid bias | | | | | Immune Rich | | | |  |  |  |  |  |
| PT_K63972GG_7316-666 | | | | Low-grade glioma/astrocytoma (WHO grade I/II) | | | | | | | 5769 | | | Immune Stromal Myeloid bias | | | | | Myeloid Centric | | | |  |  |  |  |  |
| PT_K6G6BE6G_7316-1113 | | | | Low-grade glioma/astrocytoma (WHO grade I/II) | | | | | | | 2480 | | | Immune Desert Tumor Rejected | | | | | Immune Desert | | | |  |  |  |  |  |
| PT_K94ZVZMX_7316-521 | | | | Medulloblastoma | | | | | | | 4526 | | | Tumor Rejected cDC2 bias | | | | | Immune Stromal Desert | | | |  |  |  |  |  |
| PT_KEQ53KRH_7316-440 | | | | High-grade glioma/astrocytoma (WHO grade III/IV) | | | | | | | 3424 | | | Tumor Recruitment BMDM bias | | | | | Immune Stromal Rich | | | |  |  |  |  |  |
| PT_KFYJBQB8_7316-3075 | | | | High-grade glioma/astrocytoma (WHO grade III/IV) | | | | | | | 257 | | | Tumor Recruitment BMDM bias | | | | | Myeloid Centric | | | |  |  |  |  |  |
| PT_KHX1WQ4H_7316-186 | | | | Low-grade glioma/astrocytoma (WHO grade I/II) | | | | | | | 4417 | | | Immune Stromal Tumor Recruitment | | | | | Immune Stromal Rich | | | |  |  |  |  |  |
| PT_KMHGNCNR_7316-350 | | | | Low-grade glioma/astrocytoma (WHO grade I/II) | | | | | | | 421 | | | Immune Rich Tumor Recruitment | | | | | Immune Rich | | | |  |  |  |  |  |
| PT_KMHGNCNR_7316-944 | | | | Low-grade glioma/astrocytoma (WHO grade I/II) | | | | | | | 1083 | | | Immune Rich Tumor Rich | | | | | Immune Rich | | | |  |  |  |  |  |
| PT_KN25EYMG_7316-2309 | | | | Medulloblastoma | | | | | | | 1750 | | | Immune Desert | | | | | Immune Desert | | | |  |  |  |  |  |
| PT_KQK98ZVP_7316-1803 | | | | Low-grade glioma/astrocytoma (WHO grade I/II) | | | | | | | 1119 | | | Immune Stromal Myeloid bias | | | | | Immune Stromal Rich | | | |  |  |  |  |  |
| PT_KVMF5TFW_7316-3030 | | | | High-grade glioma/astrocytoma (WHO grade III/IV) | | | | | | | 2600 | | | Stromal Centered Myeloid bias | | | | | Immune Stromal Desert | | | |  |  |  |  |  |
| PT_KX84KH6K_7316-2703 | | | | High-grade glioma/astrocytoma (WHO grade III/IV) | | | | | | | 4810 | | | Tumor Rejected OG bias | | | | | Immune Stromal Desert | | | |  |  |  |  |  |
| PT_KXB1PJV0_7316-206 | | | | Low-grade glioma/astrocytoma (WHO grade I/II) | | | | | | | 4216 | | | Immune Stromal Myeloid bias | | | | | Myeloid Centric | | | |  |  |  |  |  |
| PT_KZ04SDDS_7316-1774 | | | | High-grade glioma/astrocytoma (WHO grade III/IV) | | | | | | | 2531 | | | Tumor Recruitment BMDM bias | | | | | Myeloid Centric | | | |  |  |  |  |  |
| PT_M04EC74B_7316-918 | | | | Atypical Teratoid Rhabdoid Tumor (ATRT) | | | | | | | 544 | | | Tumor Rejected cDC2 bias | | | | | T cell Centric | | | |  |  |  |  |  |
| PT_M1RYN7B0_7316-1781 | | | | Atypical Teratoid Rhabdoid Tumor (ATRT) | | | | | | | 1038 | | | Tumor Recruitment BMDM bias | | | | | Immune Desert | | | |  |  |  |  |  |
| PT_M5HDHQNQ_7316-475 | | | | Low-grade glioma/astrocytoma (WHO grade I/II) | | | | | | | 1986 | | | Immune Stromal Myeloid bias | | | | | Immune Stromal Rich | | | |  |  |  |  |  |
| PT_MBAXTJET_7316-275 | | | | Low-grade glioma/astrocytoma (WHO grade I/II) | | | | | | | 1896 | | | Immune Stromal Rich | | | | | Immune Stromal Rich | | | |  |  |  |  |  |
| PT_MDWPRDBT_7316-2146 | | | | High-grade glioma/astrocytoma (WHO grade III/IV) | | | | | | | 1840 | | | Immune Rich Tumor Recruitment | | | | | Immune Rich | | | |  |  |  |  |  |
| PT_MDWPRDBT_7316-2660 | | | | High-grade glioma/astrocytoma (WHO grade III/IV) | | | | | | | 2119 | | | Immune Rich Tumor Recruitment | | | | | Immune Rich | | | |  |  |  |  |  |
| PT_MDWPRDBT_7316-2751 | | | | High-grade glioma/astrocytoma (WHO grade III/IV) | | | | | | | 2203 | | | Immune Stromal Tumor Recruitment | | | | | Immune Stromal Rich | | | |  |  |  |  |  |
| PT_MDWPRDBT_7316-895 | | | | High-grade glioma/astrocytoma (WHO grade III/IV) | | | | | | | 1500 | | | Tumor Recruitment BMDM bias | | | | | Immune Desert | | | |  |  |  |  |  |
| PT_MKDQZ17N_7316-3292 | | | | Ependymoma | | | | | | | 55 | | | Immune Rich Tumor Recruitment | | | | | T cell Centric | | | |  |  |  |  |  |
| PT_MKDQZ17N_7316-3300 | | | | Ependymoma | | | | | | | 54 | | | Immune Rich | | | | | Immune Rich | | | |  |  |  |  |  |
| PT_MNR94DEV_7316-370 | | | | Low-grade glioma/astrocytoma (WHO grade I/II) | | | | | | | 3870 | | | Immune Rich | | | | | Myeloid stromal Centric | | | |  |  |  |  |  |
| PT_MPRBGGEJ_7316-2172 | | | | Low-grade glioma/astrocytoma (WHO grade I/II) | | | | | | | 5937 | | | Immune Stromal Myeloid bias | | | | | Immune Rich | | | |  |  |  |  |  |
| PT_MQHST8YC_7316-3316 | | | | Craniopharyngioma | | | | | | | 2143 | | | Immune Rich | | | | | Immune Rich | | | |  |  |  |  |  |
| PT_MSBPMJ56_7316-306 | | | | Medulloblastoma | | | | | | | 3408 | | | Immune Desert Tumor Rejected | | | | | Immune Desert | | | |  |  |  |  |  |
| PT_MSQ6GSVP_7316-2274 | | | | High-grade glioma/astrocytoma (WHO grade III/IV) | | | | | | | 83 | | | Tumor Recruitment BMDM bias | | | | | Immune Desert | | | |  |  |  |  |  |
| PT_MTE126WM_7316-376 | | | | Atypical Teratoid Rhabdoid Tumor (ATRT) | | | | | | | 1936 | | | Immune Desert | | | | | Myeloid Centric | | | |  |  |  |  |  |
| PT_MXK5R7RJ_7316-1055 | | | | High-grade glioma/astrocytoma (WHO grade III/IV) | | | | | | | 5789 | | | Stromal Centered Myeloid bias | | | | | Immune Stromal Rich | | | |  |  |  |  |  |
| PT_N32BYY8A_7316-89 | | | | High-grade glioma/astrocytoma (WHO grade III/IV) | | | | | | | 6381 | | | Immune Stromal Myeloid bias | | | | | Immune Stromal Rich | | | |  |  |  |  |  |
| PT_N3HVMV06_7316-2075 | | | | Low-grade glioma/astrocytoma (WHO grade I/II) | | | | | | | 5416 | | | Immune Stromal Tumor Recruitment | | | | | Immune Stromal Rich | | | |  |  |  |  |  |
| PT_N4PR21ME_7316-330 | | | | Ependymoma | | | | | | | 887 | | | Tumor Recruitment BMDM bias | | | | | Immune Desert | | | |  |  |  |  |  |
| PT_N56EYD4Z_7316-3857 | | | | Medulloblastoma | | | | | | | 3724 | | | Immune Desert Tumor Rejected | | | | | Immune Desert | | | |  |  |  |  |  |
| PT_N673SYWW_7316-1751 | | | | Craniopharyngioma | | | | | | | 2963 | | | Immune Rich Tumor Rich | | | | | Immune Rich | | | |  |  |  |  |  |
| PT_N8W26H19_7316-267 | | | | Low-grade glioma/astrocytoma (WHO grade I/II) | | | | | | | 5401 | | | Immune Rich Tumor Recruitment | | | | | Immune Rich | | | |  |  |  |  |  |
| PT_N9CGVG02_7316-2197 | | | | Low-grade glioma/astrocytoma (WHO grade I/II) | | | | | | | 4114 | | | Immune Stromal Tumor Recruitment | | | | | Immune Stromal Rich | | | |  |  |  |  |  |
| PT_N9KYV4WB_7316-749 | | | | Ependymoma | | | | | | | 334 | | | Immune Desert | | | | | Immune Desert | | | |  |  |  |  |  |
| PT_NA8NZ0BN_7316-2135 | | | | Low-grade glioma/astrocytoma (WHO grade I/II) | | | | | | | 3641 | | | Immune Stromal Rich | | | | | Immune Stromal Rich | | | |  |  |  |  |  |
| PT_NCDHZ8H8_7316-2780 | | | | Medulloblastoma | | | | | | | 1514 | | | Tumor Rejected cDC2 bias | | | | | T cell Centric | | | |  |  |  |  |  |
| PT_ND34A6PR_7316-122 | | | | Atypical Teratoid Rhabdoid Tumor (ATRT) | | | | | | | 2982 | | | Immune Rich | | | | | Immune Rich | | | |  |  |  |  |  |
| PT_NDVJE438_7316-2811 | | | | Medulloblastoma | | | | | | | 2820 | | | Immune Desert Tumor Rejected | | | | | Immune Desert | | | |  |  |  |  |  |
| PT_NEB4WEAT_7316-3020 | | | | Craniopharyngioma | | | | | | | 4116 | | | Immune Rich | | | | | Immune Rich | | | |  |  |  |  |  |
| PT_NESAQHB1_7316-3689 | | | | Supratentorial or Spinal Cord PNET | | | | | | | 729 | | | Immune Desert Tumor Rejected | | | | | Immune Desert | | | |  |  |  |  |  |
| PT_NESAQHB1_7316-3844 | | | | Supratentorial or Spinal Cord PNET | | | | | | | 798 | | | Tumor Rejected OG bias | | | | | Immune Stromal Desert | | | |  |  |  |  |  |
| PT_NNZQ09SH_7316-1109 | | | | Low-grade glioma/astrocytoma (WHO grade I/II) | | | | | | | 4683 | | | Tumor Rejected OG bias | | | | | T cell Centric | | | |  |  |  |  |  |
| PT_NPETR8RY_7316-160 | | | | Ependymoma | | | | | | | 1223 | | | Tumor Recruitment BMDM bias | | | | | Myeloid Centric | | | |  |  |  |  |  |
| PT_NPETR8RY_7316-1784 | | | | Ependymoma | | | | | | | 2338 | | | Tumor Recruitment BMDM bias | | | | | Immune Desert | | | |  |  |  |  |  |
| PT_NPHZQW6A_7316-1985 | | | | Ganglioglioma | | | | | | | 1245 | | | Tumor Rejected OG bias | | | | | Immune Stromal Desert | | | |  |  |  |  |  |
| PT_NPPBNRVV_7316-1943 | | | | Low-grade glioma/astrocytoma (WHO grade I/II) | | | | | | | 3918 | | | Immune Rich Tumor Recruitment | | | | | Immune Rich | | | |  |  |  |  |  |
| PT_NTEKMKTX_7316-1650 | | | | Medulloblastoma | | | | | | | 167 | | | Tumor Rejected cDC2 bias | | | | | Immune Desert | | | |  |  |  |  |  |
| PT_NTK39NNV_7316-3076 | | | | Low-grade glioma/astrocytoma (WHO grade I/II) | | | | | | | 5518 | | | Immune Stromal Rich | | | | | Immune Stromal Rich | | | |  |  |  |  |  |
| PT_NV584F7Q_7316-3192 | | | | Medulloblastoma | | | | | | | 2066 | | | Immune Desert Tumor Rejected | | | | | Immune Desert | | | |  |  |  |  |  |
| PT_NYBH025T_7316-3069 | | | | Ependymoma | | | | | | | 797 | | | Stromal Centered Myeloid bias | | | | | Immune Stromal Desert | | | |  |  |  |  |  |
| PT_NZFFMZ0B_7316-923 | | | | Low-grade glioma/astrocytoma (WHO grade I/II) | | | | | | | 5524 | | | Tumor Rejected OG bias | | | | | Immune Stromal Desert | | | |  |  |  |  |  |
| PT_P2NGYMG5_7316-1956 | | | | High-grade glioma/astrocytoma (WHO grade III/IV) | | | | | | | 4559 | | | Tumor Rejected OG bias | | | | | Myeloid stromal Centric | | | |  |  |  |  |  |
| PT_P4ZEFY19_7316-937 | | | | Medulloblastoma | | | | | | | 4601 | | | Immune Desert Tumor Rejected | | | | | Immune Desert | | | |  |  |  |  |  |
| PT_P5HHJJPH_7316-870 | | | | High-grade glioma/astrocytoma (WHO grade III/IV) | | | | | | | 3739 | | | Tumor Recruitment BMDM bias | | | | | Immune Rich | | | |  |  |  |  |  |
| PT_P75ZXB1S_7316-692 | | | | Low-grade glioma/astrocytoma (WHO grade I/II) | | | | | | | 6447 | | | Immune Rich Tumor Rich | | | | | Immune Rich | | | |  |  |  |  |  |
| PT_P9RT6693_7316-2566 | | | | Low-grade glioma/astrocytoma (WHO grade I/II) | | | | | | | 3260 | | | Immune Rich Tumor Recruitment | | | | | Immune Rich | | | |  |  |  |  |  |
| PT_PABK01H0_7316-1669 | | | | Medulloblastoma | | | | | | | 1076 | | | Immune Desert Tumor Rejected | | | | | Immune Desert | | | |  |  |  |  |  |
| PT_PF9EC93Q_7316-3765 | | | | High-grade glioma/astrocytoma (WHO grade III/IV) | | | | | | | 2446 | | | Tumor Rejected OG bias | | | | | Immune Stromal Desert | | | |  |  |  |  |  |
| PT_PFA762TK_7316-368 | | | | Low-grade glioma/astrocytoma (WHO grade I/II) | | | | | | | 796 | | | Immune Stromal Myeloid bias | | | | | Myeloid stromal Centric | | | |  |  |  |  |  |
| PT_PFA762TK_7316-462 | | | | Low-grade glioma/astrocytoma (WHO grade I/II) | | | | | | | 1018 | | | Immune Stromal Rich | | | | | Immune Stromal Rich | | | |  |  |  |  |  |
| PT_PFP1ZVHD_7316-2310 | | | | Low-grade glioma/astrocytoma (WHO grade I/II) | | | | | | | 5216 | | | Immune Rich Tumor Recruitment | | | | | Immune Rich | | | |  |  |  |  |  |
| PT_PGVQ4XRZ_7316-946 | | | | Low-grade glioma/astrocytoma (WHO grade I/II) | | | | | | | 4513 | | | Immune Stromal Rich | | | | | Immune Stromal Rich | | | |  |  |  |  |  |
| PT_PHKSYE6Q_7316-220 | | | | Choroid plexus papilloma | | | | | | | 4957 | | | Tumor Rejected cDC2 bias | | | | | Immune Desert | | | |  |  |  |  |  |
| PT_PJSV0X4X_7316-843 | | | | Medulloblastoma | | | | | | | 2638 | | | Tumor Rejected OG bias | | | | | Immune Stromal Desert | | | |  |  |  |  |  |
| PT_PKP1250P_7316-2556 | | | | Ependymoma | | | | | | | 5251 | | | Tumor Rejected cDC2 bias | | | | | Immune Desert | | | |  |  |  |  |  |
| PT_PNED3GG3_7316-387 | | | | Craniopharyngioma | | | | | | | 2241 | | | Tumor Rejected cDC2 bias | | | | | T cell Centric | | | |  |  |  |  |  |
| PT_PR4YBBH3_7316-116 | | | | Brainstem glioma- Diffuse intrinsic pontine glioma | | | | | | | 3739 | | | Tumor Recruitment BMDM bias | | | | | Immune Stromal Rich | | | |  |  |  |  |  |
| PT_PR4YBBH3_7316-126 | | | | Brainstem glioma- Diffuse intrinsic pontine glioma | | | | | | | 3778 | | | Immune Rich | | | | | Myeloid stromal Centric | | | |  |  |  |  |  |
| PT_PX4E85V1_7316-1083 | | | | Medulloblastoma | | | | | | | 1680 | | | Immune Desert Tumor Rejected | | | | | Immune Desert | | | |  |  |  |  |  |
| PT_Q25V9Y6M_7316-1668 | | | | Low-grade glioma/astrocytoma (WHO grade I/II) | | | | | | | 1401 | | | Immune Stromal Myeloid bias | | | | | Immune Stromal Rich | | | |  |  |  |  |  |
| PT_Q38AT6JY_7316-3773 | | | | Craniopharyngioma | | | | | | | 1828 | | | Immune Rich | | | | | Immune Stromal Rich | | | |  |  |  |  |  |
| PT_Q3EP24V1_7316-154 | | | | Low-grade glioma/astrocytoma (WHO grade I/II) | | | | | | | 2399 | | | Immune Rich Tumor Recruitment | | | | | Immune Rich | | | |  |  |  |  |  |
| PT_Q90R6M3E_7316-897 | | | | Medulloblastoma | | | | | | | 3026 | | | Immune Desert Tumor Rejected | | | | | Immune Desert | | | |  |  |  |  |  |
| PT_QAX38GM7_7316-3570 | | | | Low-grade glioma/astrocytoma (WHO grade I/II) | | | | | | | 4786 | | | Stromal Centered Myeloid bias | | | | | Immune Stromal Rich | | | |  |  |  |  |  |
| PT_QEP13FH4_7316-3025 | | | | Medulloblastoma | | | | | | | 2696 | | | Immune Desert Tumor Rejected | | | | | Immune Desert | | | |  |  |  |  |  |
| PT_QJ3144WH_7316-3204 | | | | Choroid plexus papilloma | | | | | | | 6330 | | | Immune Desert | | | | | Immune Rich | | | |  |  |  |  |  |
| PT_QJDY4Y9P_7316-562 | | | | Low-grade glioma/astrocytoma (WHO grade I/II) | | | | | | | 4754 | | | Immune Stromal Myeloid bias | | | | | Myeloid Centric | | | |  |  |  |  |  |
| PT_QK4XB83F_7316-663 | | | | Low-grade glioma/astrocytoma (WHO grade I/II) | | | | | | | 1546 | | | Immune Rich | | | | | Immune Rich | | | |  |  |  |  |  |
| PT_QT3MK7BZ_7316-1977 | | | | Low-grade glioma/astrocytoma (WHO grade I/II) | | | | | | | 4744 | | | Immune Rich | | | | | Myeloid Centric | | | |  |  |  |  |  |
| PT_QT9T89S5_7316-297 | | | | Low-grade glioma/astrocytoma (WHO grade I/II) | | | | | | | 480 | | | Immune Stromal Myeloid bias | | | | | Myeloid stromal Centric | | | |  |  |  |  |  |
| PT_QTDY0Y9A_7316-3771 | | | | High-grade glioma/astrocytoma (WHO grade III/IV) | | | | | | | 4290 | | | Tumor Rejected OG bias | | | | | Immune Stromal Desert | | | |  |  |  |  |  |
| PT_QW5VW2PY_7316-313 | | | | Medulloblastoma | | | | | | | 3655 | | | Tumor Rejected cDC2 bias | | | | | T cell Centric | | | |  |  |  |  |  |
| PT_QWR0EECG_7316-1480 | | | | Ependymoma | | | | | | | 2082 | | | Immune Rich | | | | | Immune Stromal Rich | | | |  |  |  |  |  |
| PT_QYEF3JAT_7316-1460 | | | | Medulloblastoma | | | | | | | 4389 | | | Immune Desert Tumor Rejected | | | | | Immune Desert | | | |  |  |  |  |  |
| PT_QZN7FZER_7316-2757 | | | | Ependymoma | | | | | | | 6256 | | | Tumor Recruitment BMDM bias | | | | | T cell Centric | | | |  |  |  |  |  |
| PT_R0M1QBY4_7316-905 | | | | Ganglioglioma | | | | | | | 2964 | | | Tumor Rejected OG bias | | | | | T cell stromal Centric | | | |  |  |  |  |  |
| PT_R10BSB9F_7316-1965 | | | | Ganglioglioma | | | | | | | 451 | | | Immune Rich Tumor Recruitment | | | | | Immune Rich | | | |  |  |  |  |  |
| PT_R4T1V1K4_7316-3206 | | | | Brainstem glioma- Diffuse intrinsic pontine glioma | | | | | | | 3716 | | | Immune Desert | | | | | Immune Desert | | | |  |  |  |  |  |
| PT_R63RHJWX_7316-739 | | | | Medulloblastoma | | | | | | | 4058 | | | Tumor Rejected OG bias | | | | | Immune Stromal Desert | | | |  |  |  |  |  |
| PT_R6WWH1QX_7316-423 | | | | Ependymoma | | | | | | | 2896 | | | Immune Rich | | | | | Immune Rich | | | |  |  |  |  |  |
| PT_R9V5R1SY_7316-1940 | | | | Ganglioglioma | | | | | | | 1243 | | | Immune Rich Tumor Rich | | | | | Immune Rich | | | |  |  |  |  |  |
| PT_RA6AGBA7_7316-461 | | | | High-grade glioma/astrocytoma (WHO grade III/IV) | | | | | | | 3591 | | | Tumor Rejected OG bias | | | | | Myeloid stromal Centric | | | |  |  |  |  |  |
| PT_RDJ40M73_7316-2561 | | | | High-grade glioma/astrocytoma (WHO grade III/IV) | | | | | | | 6100 | | | Tumor Recruitment BMDM bias | | | | | Immune Desert | | | |  |  |  |  |  |
| PT_RFB07MZS_7316-2107 | | | | Medulloblastoma | | | | | | | 1385 | | | Immune Desert Tumor Rejected | | | | | Immune Desert | | | |  |  |  |  |  |
| PT_RFF7MKTC_7316-954 | | | | Low-grade glioma/astrocytoma (WHO grade I/II) | | | | | | | 1818 | | | Immune Stromal Rich | | | | | Immune Stromal Rich | | | |  |  |  |  |  |
| PT_RFGZ2JGP_7316-135 | | | | Medulloblastoma | | | | | | | 4240 | | | Tumor Rejected cDC2 bias | | | | | Immune Desert | | | |  |  |  |  |  |
| PT_RGX23JFP_7316-156 | | | | Ganglioglioma | | | | | | | 4776 | | | Tumor Recruitment BMDM bias | | | | | Immune Rich | | | |  |  |  |  |  |
| PT_RGX23JFP_7316-2901 | | | | Ganglioglioma | | | | | | | 155 | | | Immune Rich Tumor Rich | | | | | Immune Rich | | | |  |  |  |  |  |
| PT_RH59ET3M_7316-1655 | | | | Medulloblastoma | | | | | | | 3336 | | | Immune Desert Tumor Rejected | | | | | Immune Desert | | | |  |  |  |  |  |
| PT_RJ1TJ2KH_7316-2110 | | | | Atypical Teratoid Rhabdoid Tumor (ATRT) | | | | | | | 218 | | | Tumor Rejected OG bias | | | | | Immune Stromal Desert | | | |  |  |  |  |  |
| PT_RJ1TJ2KH_7316-3680 | | | | Atypical Teratoid Rhabdoid Tumor (ATRT) | | | | | | | 626 | | | Tumor Recruitment BMDM bias | | | | | Immune Stromal Rich | | | |  |  |  |  |  |
| PT_RJ98S3KZ_7316-556 | | | | Medulloblastoma | | | | | | | 4452 | | | Tumor Rejected OG bias | | | | | Immune Stromal Desert | | | |  |  |  |  |  |
| PT_RKECV80K_7316-9 | | | | Low-grade glioma/astrocytoma (WHO grade I/II) | | | | | | | 4138 | | | Tumor Recruitment BMDM bias | | | | | Myeloid Centric | | | |  |  |  |  |  |
| PT_RM5S859Q_7316-111 | | | | Ependymoma | | | | | | | 4519 | | | Tumor Rejected cDC2 bias | | | | | Immune Desert | | | |  |  |  |  |  |
| PT_RMK36R2E_7316-2275 | | | | Low-grade glioma/astrocytoma (WHO grade I/II) | | | | | | | 3746 | | | Immune Stromal Myeloid bias | | | | | Myeloid stromal Centric | | | |  |  |  |  |  |
| PT_RQ21EBK9_7316-367 | | | | Medulloblastoma | | | | | | | 3651 | | | Tumor Rejected cDC2 bias | | | | | T cell Centric | | | |  |  |  |  |  |
| PT_RRG1V7EP_7316-820 | | | | Atypical Teratoid Rhabdoid Tumor (ATRT) | | | | | | | 230 | | | Immune Desert | | | | | Immune Desert | | | |  |  |  |  |  |
| PT_RRT2E7HK_7316-136 | | | | Low-grade glioma/astrocytoma (WHO grade I/II) | | | | | | | 5970 | | | Immune Rich | | | | | Immune Stromal Rich | | | |  |  |  |  |  |
| PT_RSDTCFDA_7316-204 | | | | High-grade glioma/astrocytoma (WHO grade III/IV) | | | | | | | 4946 | | | Stromal Centered Myeloid bias | | | | | Immune Stromal Desert | | | |  |  |  |  |  |
| PT_RT1J9P8J_7316-307 | | | | Medulloblastoma | | | | | | | 2589 | | | Immune Desert Tumor Rejected | | | | | Immune Desert | | | |  |  |  |  |  |
| PT_RV0RDER2_7316-262 | | | | Low-grade glioma/astrocytoma (WHO grade I/II) | | | | | | | 853 | | | Tumor Rejected cDC2 bias | | | | | Immune Desert | | | |  |  |  |  |  |
| PT_RX1EWBP9_7316-2276 | | | | Atypical Teratoid Rhabdoid Tumor (ATRT) | | | | | | | 1876 | | | Immune Rich | | | | | Immune Stromal Rich | | | |  |  |  |  |  |
| PT_S0PBN4M7_7316-1805 | | | | Low-grade glioma/astrocytoma (WHO grade I/II) | | | | | | | 392 | | | Immune Stromal Myeloid bias | | | | | Myeloid stromal Centric | | | |  |  |  |  |  |
| PT_S0Q27J13_7316-2307 | | | | High-grade glioma/astrocytoma (WHO grade III/IV) | | | | | | | 6258 | | | Stromal Centered Myeloid bias | | | | | Immune Stromal Rich | | | |  |  |  |  |  |
| PT_S21ZTKPS_7316-362 | | | | Low-grade glioma/astrocytoma (WHO grade I/II) | | | | | | | 4938 | | | Immune Stromal Tumor Recruitment | | | | | Immune Stromal Rich | | | |  |  |  |  |  |
| PT_S2ASFBP7_7316-2157 | | | | Craniopharyngioma | | | | | | | 3355 | | | Immune Rich Tumor Recruitment | | | | | Immune Rich | | | |  |  |  |  |  |
| PT_S2BEPYQ0_7316-3000 | | | | High-grade glioma/astrocytoma (WHO grade III/IV) | | | | | | | 3431 | | | Tumor Recruitment BMDM bias | | | | | Immune Stromal Rich | | | |  |  |  |  |  |
| PT_S2CXXZCF_7316-643 | | | | Low-grade glioma/astrocytoma (WHO grade I/II) | | | | | | | 1373 | | | Immune Stromal Myeloid bias | | | | | Immune Rich | | | |  |  |  |  |  |
| PT_S2SQJVGK_7316-344 | | | | Low-grade glioma/astrocytoma (WHO grade I/II) | | | | | | | 3991 | | | Stromal Centered Myeloid bias | | | | | Immune Desert | | | |  |  |  |  |  |
| PT_S2SQJVGK_7316-442 | | | | Low-grade glioma/astrocytoma (WHO grade I/II) | | | | | | | 4222 | | | Immune Rich Tumor Rich | | | | | Immune Rich | | | |  |  |  |  |  |
| PT_S3FJSBJT_7316-1794 | | | | Low-grade glioma/astrocytoma (WHO grade I/II) | | | | | | | 6188 | | | Immune Rich Tumor Rich | | | | | Immune Rich | | | |  |  |  |  |  |
| PT_S4H6KA09_7316-2134 | | | | Ependymoma | | | | | | | 1412 | | | Tumor Rejected OG bias | | | | | Immune Stromal Desert | | | |  |  |  |  |  |
| PT_S4YNE17X_7316-2899 | | | | Low-grade glioma/astrocytoma (WHO grade I/II) | | | | | | | 1571 | | | Tumor Rejected cDC2 bias | | | | | T cell Centric | | | |  |  |  |  |  |
| PT_S7WJ99PC_7316-677 | | | | Low-grade glioma/astrocytoma (WHO grade I/II) | | | | | | | 2936 | | | Immune Rich Tumor Recruitment | | | | | Myeloid Centric | | | |  |  |  |  |  |
| PT_SBHN5F68_7316-516 | | | | Low-grade glioma/astrocytoma (WHO grade I/II) | | | | | | | 2539 | | | Immune Stromal Myeloid bias | | | | | Myeloid stromal Centric | | | |  |  |  |  |  |
| PT_SBP79T7F_7316-315 | | | | Medulloblastoma | | | | | | | 3542 | | | Tumor Rejected OG bias | | | | | T cell stromal Centric | | | |  |  |  |  |  |
| PT_SDK91A16_7316-247 | | | | Low-grade glioma/astrocytoma (WHO grade I/II) | | | | | | | 5017 | | | Immune Stromal Rich | | | | | Immune Stromal Rich | | | |  |  |  |  |  |
| PT_SDPA6AAP_7316-624 | | | | High-grade glioma/astrocytoma (WHO grade III/IV) | | | | | | | 3740 | | | Tumor Rejected OG bias | | | | | Immune Stromal Desert | | | |  |  |  |  |  |
| PT_SJEG0MKD_7316-146 | | | | Low-grade glioma/astrocytoma (WHO grade I/II) | | | | | | | 2396 | | | Immune Stromal Tumor Recruitment | | | | | Immune Stromal Rich | | | |  |  |  |  |  |
| PT_SMDEQVHJ_7316-119 | | | | Low-grade glioma/astrocytoma (WHO grade I/II) | | | | | | | 675 | | | Immune Stromal Rich | | | | | Immune Stromal Rich | | | |  |  |  |  |  |
| PT_SRWZDG3N_7316-361 | | | | Low-grade glioma/astrocytoma (WHO grade I/II) | | | | | | | 5666 | | | Immune Stromal Tumor Recruitment | | | | | Immune Stromal Rich | | | |  |  |  |  |  |
| PT_STRDJQ01_7316-1038 | | | | Medulloblastoma | | | | | | | 475 | | | Immune Desert Tumor Rejected | | | | | Immune Desert | | | |  |  |  |  |  |
| PT_T03AT4BJ_7316-2708 | | | | Medulloblastoma | | | | | | | 4428 | | | Immune Desert Tumor Rejected | | | | | Immune Desert | | | |  |  |  |  |  |
| PT_T1FV709B_7316-681 | | | | Low-grade glioma/astrocytoma (WHO grade I/II) | | | | | | | 2217 | | | Immune Stromal Myeloid bias | | | | | Immune Rich | | | |  |  |  |  |  |
| PT_T2M1338J_7316-258 | | | | Craniopharyngioma | | | | | | | 4342 | | | Immune Rich | | | | | Immune Stromal Rich | | | |  |  |  |  |  |
| PT_T3REPTTC_7316-3488 | | | | Medulloblastoma | | | | | | | 754 | | | Immune Desert Tumor Rejected | | | | | Immune Desert | | | |  |  |  |  |  |
| PT_T4VKB29K_7316-2125 | | | | Ependymoma | | | | | | | 1873 | | | Tumor Recruitment BMDM bias | | | | | Myeloid Centric | | | |  |  |  |  |  |
| PT_T8V9ES93_7316-1106 | | | | High-grade glioma/astrocytoma (WHO grade III/IV) | | | | | | | 6081 | | | Immune Rich Tumor Rich | | | | | Immune Rich | | | |  |  |  |  |  |
| PT_TB8HPJBN_7316-2663 | | | | Low-grade glioma/astrocytoma (WHO grade I/II) | | | | | | | 1436 | | | Immune Stromal Myeloid bias | | | | | Myeloid stromal Centric | | | |  |  |  |  |  |
| PT_TD6ST5MG_7316-235 | | | | Low-grade glioma/astrocytoma (WHO grade I/II) | | | | | | | 129 | | | Stromal Centered Myeloid bias | | | | | Myeloid stromal Centric | | | |  |  |  |  |  |
| PT_TEBD1SES_7316-3054 | | | | Low-grade glioma/astrocytoma (WHO grade I/II) | | | | | | | 1038 | | | Immune Rich | | | | | Immune Stromal Rich | | | |  |  |  |  |  |
| PT_TH18S0JQ_7316-3068 | | | | Low-grade glioma/astrocytoma (WHO grade I/II) | | | | | | | 4932 | | | Tumor Rejected OG bias | | | | | T cell stromal Centric | | | |  |  |  |  |  |
| PT_TH8M3W34_7316-2130 | | | | Low-grade glioma/astrocytoma (WHO grade I/II) | | | | | | | 2834 | | | Immune Stromal Tumor Recruitment | | | | | Immune Stromal Rich | | | |  |  |  |  |  |
| PT_TKWTTRQ7_7316-2971 | | | | Medulloblastoma | | | | | | | 5075 | | | Tumor Rejected cDC2 bias | | | | | T cell Centric | | | |  |  |  |  |  |
| PT_TPDB1WC8_7316-2777 | | | | High-grade glioma/astrocytoma (WHO grade III/IV) | | | | | | | 1845 | | | Tumor Rejected cDC2 bias | | | | | Immune Desert | | | |  |  |  |  |  |
| PT_TPSMT960_7316-400 | | | | Low-grade glioma/astrocytoma (WHO grade I/II) | | | | | | | 637 | | | Immune Stromal Myeloid bias | | | | | Immune Stromal Rich | | | |  |  |  |  |  |
| PT_TS0FW57Z_7316-2167 | | | | Low-grade glioma/astrocytoma (WHO grade I/II) | | | | | | | 5766 | | | Immune Rich Tumor Rich | | | | | Immune Rich | | | |  |  |  |  |  |
| PT_TS0H697Y_7316-394 | | | | Ependymoma | | | | | | | 4379 | | | Tumor Recruitment BMDM bias | | | | | Immune Rich | | | |  |  |  |  |  |
| PT_TV5DYHNC_7316-1073 | | | | Atypical Teratoid Rhabdoid Tumor (ATRT) | | | | | | | 1113 | | | Immune Rich | | | | | Immune Stromal Rich | | | |  |  |  |  |  |
| PT_TVK2KY36_7316-2626 | | | | Medulloblastoma | | | | | | | 3630 | | | Tumor Rejected OG bias | | | | | Immune Stromal Desert | | | |  |  |  |  |  |
| PT_TXFYRDP4_7316-193 | | | | Low-grade glioma/astrocytoma (WHO grade I/II) | | | | | | | 1232 | | | Stromal Centered Myeloid bias | | | | | Immune Stromal Rich | | | |  |  |  |  |  |
| PT_V07ZH5JS_7316-472 | | | | Low-grade glioma/astrocytoma (WHO grade I/II) | | | | | | | 5399 | | | Immune Rich Tumor Rich | | | | | Immune Rich | | | |  |  |  |  |  |
| PT_V0X73HEQ_7316-2182 | | | | Ependymoma | | | | | | | 1163 | | | Tumor Recruitment BMDM bias | | | | | Immune Desert | | | |  |  |  |  |  |
| PT_V2F0865M_7316-642 | | | | Low-grade glioma/astrocytoma (WHO grade I/II) | | | | | | | 2196 | | | Tumor Rejected OG bias | | | | | Immune Stromal Desert | | | |  |  |  |  |  |
| PT_V3Q78E6F_7316-455 | | | | Ependymoma | | | | | | | 337 | | | Immune Rich Tumor Recruitment | | | | | Immune Rich | | | |  |  |  |  |  |
| PT_V8714N9H_7316-2322 | | | | Medulloblastoma | | | | | | | 761 | | | Immune Desert Tumor Rejected | | | | | Immune Desert | | | |  |  |  |  |  |
| PT_VABP80N2_7316-1949 | | | | Low-grade glioma/astrocytoma (WHO grade I/II) | | | | | | | 4443 | | | Immune Rich | | | | | Immune Stromal Rich | | | |  |  |  |  |  |
| PT_VBG3Z18B_7316-512 | | | | Supratentorial or Spinal Cord PNET | | | | | | | 2784 | | | Stromal Centered Myeloid bias | | | | | Immune Stromal Desert | | | |  |  |  |  |  |
| PT_VBNP6JFB_7316-1095 | | | | Low-grade glioma/astrocytoma (WHO grade I/II) | | | | | | | 371 | | | Immune Stromal Tumor Recruitment | | | | | Immune Stromal Rich | | | |  |  |  |  |  |
| PT_VBVZ2NAR_7316-2156 | | | | Ependymoma | | | | | | | 230 | | | Tumor Rejected cDC2 bias | | | | | Immune Desert | | | |  |  |  |  |  |
| PT_VCD0W8HG_7316-2756 | | | | High-grade glioma/astrocytoma (WHO grade III/IV) | | | | | | | 4320 | | | Stromal Centered Myeloid bias | | | | | Myeloid stromal Centric | | | |  |  |  |  |  |
| PT_VE0Q731Y_7316-1641 | | | | Ependymoma | | | | | | | 1464 | | | Tumor Recruitment BMDM bias | | | | | Immune Desert | | | |  |  |  |  |  |
| PT_VGXHFMND_7316-1643 | | | | Low-grade glioma/astrocytoma (WHO grade I/II) | | | | | | | 4642 | | | Immune Stromal Myeloid bias | | | | | Immune Stromal Rich | | | |  |  |  |  |  |
| PT_VH6S71AC_7316-735 | | | | Medulloblastoma | | | | | | | 2590 | | | Immune Desert Tumor Rejected | | | | | Immune Desert | | | |  |  |  |  |  |
| PT_VJ0M4E8Y_7316-2099 | | | | Low-grade glioma/astrocytoma (WHO grade I/II) | | | | | | | 2154 | | | Immune Stromal Myeloid bias | | | | | Immune Stromal Desert | | | |  |  |  |  |  |
| PT_VJBVXB08_7316-155 | | | | Supratentorial or Spinal Cord PNET | | | | | | | 1039 | | | Tumor Rejected OG bias | | | | | T cell stromal Centric | | | |  |  |  |  |  |
| PT_VJZN69T9_7316-3510 | | | | Medulloblastoma | | | | | | | 880 | | | Immune Desert Tumor Rejected | | | | | Immune Desert | | | |  |  |  |  |  |
| PT_VR8T8JX8_7316-762 | | | | Ependymoma | | | | | | | 3521 | | | Tumor Rejected cDC2 bias | | | | | Immune Desert | | | |  |  |  |  |  |
| PT_VT6PJVQN_7316-205 | | | | Craniopharyngioma | | | | | | | 2759 | | | Immune Rich | | | | | Immune Stromal Rich | | | |  |  |  |  |  |
| PT_VTF65Y63_7316-3513 | | | | Craniopharyngioma | | | | | | | 3195 | | | Tumor Recruitment BMDM bias | | | | | Immune Stromal Rich | | | |  |  |  |  |  |
| PT_VWZZTK38_7316-1844 | | | | High-grade glioma/astrocytoma (WHO grade III/IV) | | | | | | | 4231 | | | Tumor Rejected cDC2 bias | | | | | T cell Centric | | | |  |  |  |  |  |
| PT_VY45XTT7_7316-1801 | | | | Medulloblastoma | | | | | | | 4284 | | | Tumor Rejected cDC2 bias | | | | | Immune Desert | | | |  |  |  |  |  |
| PT_W17NV5YG_7316-2079 | | | | Ependymoma | | | | | | | 102 | | | Tumor Recruitment BMDM bias | | | | | T cell stromal Centric | | | |  |  |  |  |  |
| PT_W17NV5YG_7316-2106 | | | | Ependymoma | | | | | | | 221 | | | Immune Desert Tumor Rejected | | | | | Immune Desert | | | |  |  |  |  |  |
| PT_W3FSA7NH_7316-3522 | | | | Low-grade glioma/astrocytoma (WHO grade I/II) | | | | | | | 3271 | | | Immune Rich Tumor Recruitment | | | | | Immune Rich | | | |  |  |  |  |  |
| PT_W4XVT4AJ_7316-548 | | | | Brainstem glioma- Diffuse intrinsic pontine glioma | | | | | | | 2020 | | | Immune Rich Tumor Recruitment | | | | | Immune Rich | | | |  |  |  |  |  |
| PT_W6AWJJK7_7316-230 | | | | Ganglioglioma | | | | | | | 271 | | | Immune Stromal Rich | | | | | Immune Stromal Rich | | | |  |  |  |  |  |
| PT_W6AWJJK7_7316-893 | | | | Ganglioglioma | | | | | | | 2816 | | | Immune Stromal Tumor Recruitment | | | | | Immune Stromal Rich | | | |  |  |  |  |  |
| PT_W6CW60K9_7316-380 | | | | Craniopharyngioma | | | | | | | 2541 | | | Immune Rich | | | | | Immune Rich | | | |  |  |  |  |  |
| PT_WG2Z95B5_7316-2141 | | | | Atypical Teratoid Rhabdoid Tumor (ATRT) | | | | | | | 383 | | | Tumor Rejected cDC2 bias | | | | | T cell Centric | | | |  |  |  |  |  |
| PT_WGWMA5AZ_7316-148 | | | | Low-grade glioma/astrocytoma (WHO grade I/II) | | | | | | | 5984 | | | Stromal Centered Myeloid bias | | | | | T cell stromal Centric | | | |  |  |  |  |  |
| PT_WKNKNYHH_7316-1107 | | | | Ependymoma | | | | | | | 888 | | | Tumor Recruitment BMDM bias | | | | | Immune Rich | | | |  |  |  |  |  |
| PT_WPYCXMDA_7316-3891 | | | | Craniopharyngioma | | | | | | | 5628 | | | Tumor Rejected OG bias | | | | | T cell stromal Centric | | | |  |  |  |  |  |
| PT_WVC247PK_7316-1090 | | | | Low-grade glioma/astrocytoma (WHO grade I/II) | | | | | | | 1024 | | | Immune Stromal Myeloid bias | | | | | Immune Stromal Rich | | | |  |  |  |  |  |
| PT_WWME595X_7316-245 | | | | Choroid plexus papilloma | | | | | | | 4259 | | | Immune Rich | | | | | Immune Rich | | | |  |  |  |  |  |
| PT_WWZ2Q14R_7316-3138 | | | | Medulloblastoma | | | | | | | 306 | | | Immune Desert Tumor Rejected | | | | | Immune Desert | | | |  |  |  |  |  |
| PT_WY8REZ3P_7316-1783 | | | | Ganglioglioma | | | | | | | 5210 | | | Tumor Rejected OG bias | | | | | Immune Stromal Desert | | | |  |  |  |  |  |
| PT_WYF217NZ_7316-345 | | | | Low-grade glioma/astrocytoma (WHO grade I/II) | | | | | | | 2478 | | | Stromal Centered Myeloid bias | | | | | Myeloid Centric | | | |  |  |  |  |  |
| PT_WYR1DVHX_7316-1950 | | | | Craniopharyngioma | | | | | | | 2537 | | | Immune Rich | | | | | Immune Rich | | | |  |  |  |  |  |
| PT_WYVMWYVA_7316-257 | | | | Choroid plexus papilloma | | | | | | | 1223 | | | Immune Desert | | | | | Myeloid Centric | | | |  |  |  |  |  |
| PT_X1PRY0FC_7316-1779 | | | | Low-grade glioma/astrocytoma (WHO grade I/II) | | | | | | | 736 | | | Immune Stromal Rich | | | | | Immune Stromal Rich | | | |  |  |  |  |  |
| PT_X1XVE1XX_7316-3511 | | | | Medulloblastoma | | | | | | | 4917 | | | Immune Desert Tumor Rejected | | | | | Immune Desert | | | |  |  |  |  |  |
| PT_X5TQR8V2_7316-343 | | | | High-grade glioma/astrocytoma (WHO grade III/IV) | | | | | | | 6470 | | | Tumor Recruitment BMDM bias | | | | | Immune Desert | | | |  |  |  |  |  |
| PT_X648RVMK_7316-238 | | | | High-grade glioma/astrocytoma (WHO grade III/IV) | | | | | | | 1732 | | | Tumor Recruitment BMDM bias | | | | | Immune Desert | | | |  |  |  |  |  |
| PT_X71D7G5S_7316-1105 | | | | Supratentorial or Spinal Cord PNET | | | | | | | 5422 | | | Stromal Centered Myeloid bias | | | | | T cell stromal Centric | | | |  |  |  |  |  |
| PT_X7HC5YCY_7316-255 | | | | High-grade glioma/astrocytoma (WHO grade III/IV) | | | | | | | 6096 | | | Tumor Rejected cDC2 bias | | | | | Immune Stromal Desert | | | |  |  |  |  |  |
| PT_XA8ASFJK_7316-3762 | | | | Low-grade glioma/astrocytoma (WHO grade I/II) | | | | | | | 6166 | | | Immune Stromal Tumor Recruitment | | | | | Immune Stromal Rich | | | |  |  |  |  |  |
| PT_XA98HG1C_7316-3023 | | | | Medulloblastoma | | | | | | | 4021 | | | Tumor Rejected OG bias | | | | | Immune Stromal Desert | | | |  |  |  |  |  |
| PT_XA98HG1C_7316-931 | | | | Medulloblastoma | | | | | | | 3332 | | | Immune Desert Tumor Rejected | | | | | Immune Desert | | | |  |  |  |  |  |
| PT_XDV8W16W_7316-3751 | | | | Low-grade glioma/astrocytoma (WHO grade I/II) | | | | | | | 5108 | | | Immune Stromal Tumor Recruitment | | | | | Immune Stromal Rich | | | |  |  |  |  |  |
| PT_XKVFVBY4_7316-550 | | | | Brainstem glioma- Diffuse intrinsic pontine glioma | | | | | | | 2372 | | | Tumor Rejected OG bias | | | | | Immune Stromal Desert | | | |  |  |  |  |  |
| PT_XNMKQ4MR_7316-2006 | | | | Low-grade glioma/astrocytoma (WHO grade I/II) | | | | | | | 1918 | | | Immune Stromal Myeloid bias | | | | | Immune Stromal Rich | | | |  |  |  |  |  |
| PT_XRQHMBHB_7316-3937 | | | | Atypical Teratoid Rhabdoid Tumor (ATRT) | | | | | | | 2847 | | | Immune Rich | | | | | Immune Rich | | | |  |  |  |  |  |
| PT_XSS1ANXD_7316-495 | | | | Low-grade glioma/astrocytoma (WHO grade I/II) | | | | | | | 1391 | | | Immune Stromal Myeloid bias | | | | | Immune Stromal Rich | | | |  |  |  |  |  |
| PT_XTVQB9S4_7316-2186 | | | | Atypical Teratoid Rhabdoid Tumor (ATRT) | | | | | | | 291 | | | Immune Desert | | | | | Immune Desert | | | |  |  |  |  |  |
| PT_XTVQB9S4_7316-2933 | | | | Atypical Teratoid Rhabdoid Tumor (ATRT) | | | | | | | 535 | | | Immune Desert | | | | | Immune Desert | | | |  |  |  |  |  |
| PT_XWVQW6JX_7316-3556 | | | | Low-grade glioma/astrocytoma (WHO grade I/II) | | | | | | | 6149 | | | Stromal Centered Myeloid bias | | | | | Immune Stromal Rich | | | |  |  |  |  |  |
| PT_XX2VW3D9_7316-96 | | | | Medulloblastoma | | | | | | | 2038 | | | Immune Desert | | | | | Immune Desert | | | |  |  |  |  |  |
| PT_XXZHRPNM_7316-2572 | | | | Low-grade glioma/astrocytoma (WHO grade I/II) | | | | | | | 1667 | | | Immune Stromal Rich | | | | | Immune Stromal Rich | | | |  |  |  |  |  |
| PT_Y4FN0XN1_7316-1252 | | | | Ependymoma | | | | | | | 4262 | | | Immune Rich | | | | | Immune Rich | | | |  |  |  |  |  |
| PT_Y572BRC1_7316-2147 | | | | Low-grade glioma/astrocytoma (WHO grade I/II) | | | | | | | 4520 | | | Immune Rich Tumor Rich | | | | | Immune Rich | | | |  |  |  |  |  |
| PT_Y572BRC1_7316-2193 | | | | Low-grade glioma/astrocytoma (WHO grade I/II) | | | | | | | 4676 | | | Immune Rich Tumor Rich | | | | | Immune Rich | | | |  |  |  |  |  |
| PT_Y572BRC1_7316-485 | | | | Low-grade glioma/astrocytoma (WHO grade I/II) | | | | | | | 3977 | | | Immune Rich Tumor Rich | | | | | Immune Rich | | | |  |  |  |  |  |
| PT_Y660CKCB_7316-3122 | | | | High-grade glioma/astrocytoma (WHO grade III/IV) | | | | | | | 5769 | | | Tumor Rejected cDC2 bias | | | | | Immune Desert | | | |  |  |  |  |  |
| PT_Y6Y9JJ9P_7316-425 | | | | Ependymoma | | | | | | | 3013 | | | Immune Rich | | | | | Immune Stromal Rich | | | |  |  |  |  |  |
| PT_Y76A7PBA_7316-2288 | | | | High-grade glioma/astrocytoma (WHO grade III/IV) | | | | | | | 4166 | | | Tumor Recruitment BMDM bias | | | | | Immune Desert | | | |  |  |  |  |  |
| PT_Y7F2JZMQ_7316-2680 | | | | Ganglioglioma | | | | | | | 6132 | | | Immune Stromal Tumor Recruitment | | | | | Immune Stromal Rich | | | |  |  |  |  |  |
| PT_Y8HSH496_7316-196 | | | | Low-grade glioma/astrocytoma (WHO grade I/II) | | | | | | | 3109 | | | Immune Stromal Myeloid bias | | | | | Immune Stromal Rich | | | |  |  |  |  |  |
| PT_Y8RNS9JE_7316-1656 | | | | High-grade glioma/astrocytoma (WHO grade III/IV) | | | | | | | 3215 | | | Immune Rich | | | | | Immune Rich | | | |  |  |  |  |  |
| PT_YBJYA2CD_7316-2244 | | | | Choroid plexus papilloma | | | | | | | 5456 | | | Immune Rich | | | | | Immune Desert | | | |  |  |  |  |  |
| PT_YFS3AWC0_7316-153 | | | | Medulloblastoma | | | | | | | 1575 | | | Immune Desert | | | | | Immune Desert | | | |  |  |  |  |  |
| PT_YGN06RPZ_7316-2204 | | | | Low-grade glioma/astrocytoma (WHO grade I/II) | | | | | | | 1383 | | | Immune Rich | | | | | Immune Stromal Rich | | | |  |  |  |  |  |
| PT_YGN06RPZ_7316-2208 | | | | Low-grade glioma/astrocytoma (WHO grade I/II) | | | | | | | 2740 | | | Immune Stromal Rich | | | | | Immune Stromal Rich | | | |  |  |  |  |  |
| PT_YHTK536P_7316-1714 | | | | Medulloblastoma | | | | | | | 1711 | | | Immune Desert Tumor Rejected | | | | | Immune Desert | | | |  |  |  |  |  |
| PT_YJGTKDV0_7316-1683 | | | | Low-grade glioma/astrocytoma (WHO grade I/II) | | | | | | | 2520 | | | Immune Rich Tumor Rich | | | | | Immune Rich | | | |  |  |  |  |  |
| PT_YKVK5HTK_7316-441 | | | | Low-grade glioma/astrocytoma (WHO grade I/II) | | | | | | | 2211 | | | Immune Stromal Rich | | | | | Immune Stromal Rich | | | |  |  |  |  |  |
| PT_YMN5GV47_7316-658 | | | | Low-grade glioma/astrocytoma (WHO grade I/II) | | | | | | | 1455 | | | Immune Rich Tumor Rich | | | | | Immune Rich | | | |  |  |  |  |  |
| PT_YPCR2XRS_7316-554 | | | | Low-grade glioma/astrocytoma (WHO grade I/II) | | | | | | | 6437 | | | Immune Stromal Rich | | | | | Immune Stromal Rich | | | |  |  |  |  |  |
| PT_YRMG61G8_7316-2305 | | | | Low-grade glioma/astrocytoma (WHO grade I/II) | | | | | | | 2264 | | | Immune Stromal Myeloid bias | | | | | Immune Stromal Rich | | | |  |  |  |  |  |
| PT_YVG4H0F1_7316-3070 | | | | Craniopharyngioma | | | | | | | 5536 | | | Immune Rich | | | | | Immune Stromal Rich | | | |  |  |  |  |  |
| PT_YXJQGT69_7316-934 | | | | Low-grade glioma/astrocytoma (WHO grade I/II) | | | | | | | 6236 | | | Stromal Centered Myeloid bias | | | | | Immune Stromal Rich | | | |  |  |  |  |  |
| PT_YZ8A8A36_7316-723 | | | | Choroid plexus carcinoma | | | | | | | 643 | | | Tumor Rejected cDC2 bias | | | | | Immune Rich | | | |  |  |  |  |  |
| PT_YZME6QCY_7316-2245 | | | | Low-grade glioma/astrocytoma (WHO grade I/II) | | | | | | | 6001 | | | Tumor Rejected OG bias | | | | | Immune Stromal Desert | | | |  |  |  |  |  |
| PT_Z4BF2NSB_7316-161 | | | | High-grade glioma/astrocytoma (WHO grade III/IV) | | | | | | | 5908 | | | Immune Rich Tumor Recruitment | | | | | Immune Rich | | | |  |  |  |  |  |
| PT_Z9QE51C3_7316-373 | | | | Ganglioglioma | | | | | | | 1400 | | | Tumor Rejected cDC2 bias | | | | | T cell stromal Centric | | | |  |  |  |  |  |
| PT_ZA95JQEB_7316-384 | | | | Ependymoma | | | | | | | 2589 | | | Tumor Recruitment BMDM bias | | | | | Myeloid Centric | | | |  |  |  |  |  |
| PT_ZD45GXZ0_7316-2109 | | | | Ependymoma | | | | | | | 1860 | | | Tumor Rejected cDC2 bias | | | | | T cell Centric | | | |  |  |  |  |  |
| PT_ZD9AYB6F_7316-3323 | | | | Low-grade glioma/astrocytoma (WHO grade I/II) | | | | | | | 1850 | | | Immune Stromal Myeloid bias | | | | | Immune Stromal Rich | | | |  |  |  |  |  |
| PT_ZG4J0EK0_7316-3774 | | | | Medulloblastoma | | | | | | | 2050 | | | Immune Desert Tumor Rejected | | | | | Immune Desert | | | |  |  |  |  |  |
| PT_ZMKMKCFQ_7316-2753 | | | | Low-grade glioma/astrocytoma (WHO grade I/II) | | | | | | | 6135 | | | Immune Desert Tumor Rejected | | | | | Immune Desert | | | |  |  |  |  |  |
| PT_ZMKMKCFQ_7316-2988 | | | | Low-grade glioma/astrocytoma (WHO grade I/II) | | | | | | | 3924 | | | Immune Rich Tumor Recruitment | | | | | Immune Rich | | | |  |  |  |  |  |
| PT_ZR8MQRVG_7316-346 | | | | Low-grade glioma/astrocytoma (WHO grade I/II) | | | | | | | 3578 | | | Immune Rich Tumor Rich | | | | | Immune Rich | | | |  |  |  |  |  |
| PT_ZRQQC2S9_7316-3139 | | | | Low-grade glioma/astrocytoma (WHO grade I/II) | | | | | | | 5407 | | | Immune Stromal Tumor Recruitment | | | | | Myeloid stromal Centric | | | |  |  |  |  |  |
| PT_ZS2YDFBE_7316-2860 | | | | Choroid plexus papilloma | | | | | | | 5917 | | | Immune Rich | | | | | Myeloid Centric | | | |  |  |  |  |  |
| PT_ZVV78QP5_7316-117 | | | | Low-grade glioma/astrocytoma (WHO grade I/II) | | | | | | | 3354 | | | Stromal Centered Myeloid bias | | | | | Immune Stromal Desert | | | |  |  |  |  |  |
| PT_ZVYRFGEN_7316-1068 | | | | High-grade glioma/astrocytoma (WHO grade III/IV) | | | | | | | 1407 | | | Stromal Centered Myeloid bias | | | | | Myeloid stromal Centric | | | |  |  |  |  |  |
| PT_ZWY04DCR_7316-2666 | | | | Ependymoma | | | | | | | 6022 | | | Tumor Rejected cDC2 bias | | | | | Immune Desert | | | |  |  |  |  |  |
| PT_ZZ8SBJJ5_7316-725 | | | | Choroid plexus papilloma | | | | | | | 881 | | | Tumor Rejected OG bias | | | | | Immune Stromal Desert | | | |  |  |  |  |  |
| PT_ZZRBX5JT_7316-2661 | | | | Ependymoma | | | | | | | 2667 | | | Tumor Recruitment BMDM bias | | | | | Immune Desert | | | |  |  |  |  |  |
| PT_ZZRBX5JT_7316-3319 | | | | Ependymoma | | | | | | | 2967 | | | Immune Desert | | | | | Immune Desert | | | |  |  |  |  |  |
| PT_ZZRBX5JT_7316-496 | | | | Ependymoma | | | | | | | 1878 | | | Tumor Recruitment BMDM bias | | | | | Immune Rich | | | |  |  |  |  |  |
| PT_ZZTQQVCT_7316-2723 | | | | Low-grade glioma/astrocytoma (WHO grade I/II) | | | | | | | 4326 | | | Immune Rich | | | | | Immune Stromal Rich | | | |  |  |  |  |  |
| PT_ZZWYKZZ8_7316-2758 | | | | Ependymoma | | | | | | | 3941 | | | Tumor Rejected OG bias | | | | | Immune Stromal Desert | | | |  |  |  |  |  |

| **Supplemental Table 4. Gene features of 12-immune pattern** | | | | | | | | | | | |
| --- | --- | --- | --- | --- | --- | --- | --- | --- | --- | --- | --- |
| **Immune Desert** | **Immune Desert Tumor Rejected** | **Immune Rich** | **Immune Rich Tumor Recruitment** | **Immune Rich Tumor Rich** | **Immune Stromal Myeloid bias** | **Immune Stromal Rich** | **Immune Stromal Tumor Recruitment** | **Stromal Centered Myeloid bias** | **Tumor Recruitment BMDM bias** | **Tumor Rejected cDC2 bias** | **Tumor Rejected OG bias** |
| CCDC33 | STMN1 | SPINT2 | GDF15 | HOPX | GPR17 | APLNR | C1orf87 | GSX1 | VEGFA | TWIST1 | SYT1 |
| AP003396.2 | AFDN | FAM83H | AL163636.1 | FETUB | AL118505.1 | HMCN2 | TNFSF14 | SLC6A11 | KCNG1 | CD24 | CNTNAP4 |
| AC005487.1 | ZNF300 | LPIN3 | FNDC1 | SNTG1 | FERMT1 | AC124804.1 | AC007325.1 | TECTB | LAMA1 | MIR217HG | PPP2R2C |
| AC013470.2 | RBFOX2 | S100A9 |  | IGF2BP2 | KIF19 | LRRC55 |  | LINC01235 | LOX | SLC6A3 | SNCB |
| WNT11 | MLLT11 | TXK |  | GLDCP1 | AC009041.2 |  |  | ARHGAP36 | MECOM | SIM1 | ARHGDIG |
| ANKRD26P4 | PPM1E | PROSER2 |  | FJX1 | LINC01254 |  |  |  | AC092675.1 | PRL | PDIA2 |
|  | FAM117B | ITGA2 |  | CPNE8 | SAPCD2 |  |  |  | FAM110C | XKR5 | CHD5 |
|  | CELSR3 | MPZL2 |  | GLDC | SMOC1 |  |  |  | ANGPTL1 | AL157895.1 | NPM2 |
|  | MKRN3 | ACP5 |  | HNF4G | C2orf27B |  |  |  | EYA4 |  | GABRA2 |
|  | HIST1H4C | SDC1 |  |  | KRT8P42 |  |  |  | CA9 |  | GRIN1 |
|  | RACGAP1 | PERP |  |  | TMC2 |  |  |  | CHRDL2 |  | SEC14L5 |
|  | YPEL1 | CAPN12 |  |  | ATP2C2 |  |  |  | AL136131.3 |  | KCNJ3 |
|  | ZNF737 | SPINT1 |  |  |  |  |  |  | CXCL5 |  | CACNA1I |
|  | RNF165 | VGLL3 |  |  |  |  |  |  | AL050404.1 |  | VWA5B2 |
|  | SOX11 | RHOD |  |  |  |  |  |  | AC005532.1 |  | KIAA1644 |
|  | GPRIN1 | LAMA3 |  |  |  |  |  |  | DTHD1 |  | FAM131C |
|  | JPT1 | MICALCL |  |  |  |  |  |  | AC239859.6 |  | SYT5 |
|  | ZNF286B | IL1R2 |  |  |  |  |  |  | OTOS |  | NKAIN2 |
|  | CELF3 | LILRB5 |  |  |  |  |  |  | HIF1A-AS2 |  | MOBP |
|  | HIST1H2BG | CLDN7 |  |  |  |  |  |  | AC097713.1 |  | HMGCLL1 |
