# Supplementary material for: The unique immune ecosystems in pediatric brain tumors: integrating single-cell and bulk RNA-sequencing
Source: Front Immunol. 2023 Nov 29;14:1238684. doi: 10.3389/fimmu.2023.1238684 (PMC10716463; doi:10.3389/fimmu.2023.1238684)
Supplement: Supplementary Figure 1 — The comparison of two methods-clustering-based (CB) method and positive expression of marker genes (PEMG) method. X axis represents the parameters of marker genes, cell types and corresponding marker pathways, and the y axis represents the ratio of the parameters between the two methods. The red line represents the mean ratio of all parameters. [file DataSheet_1.docx]

**Supplemental Figures**

**Supplemental Figure 1**

**
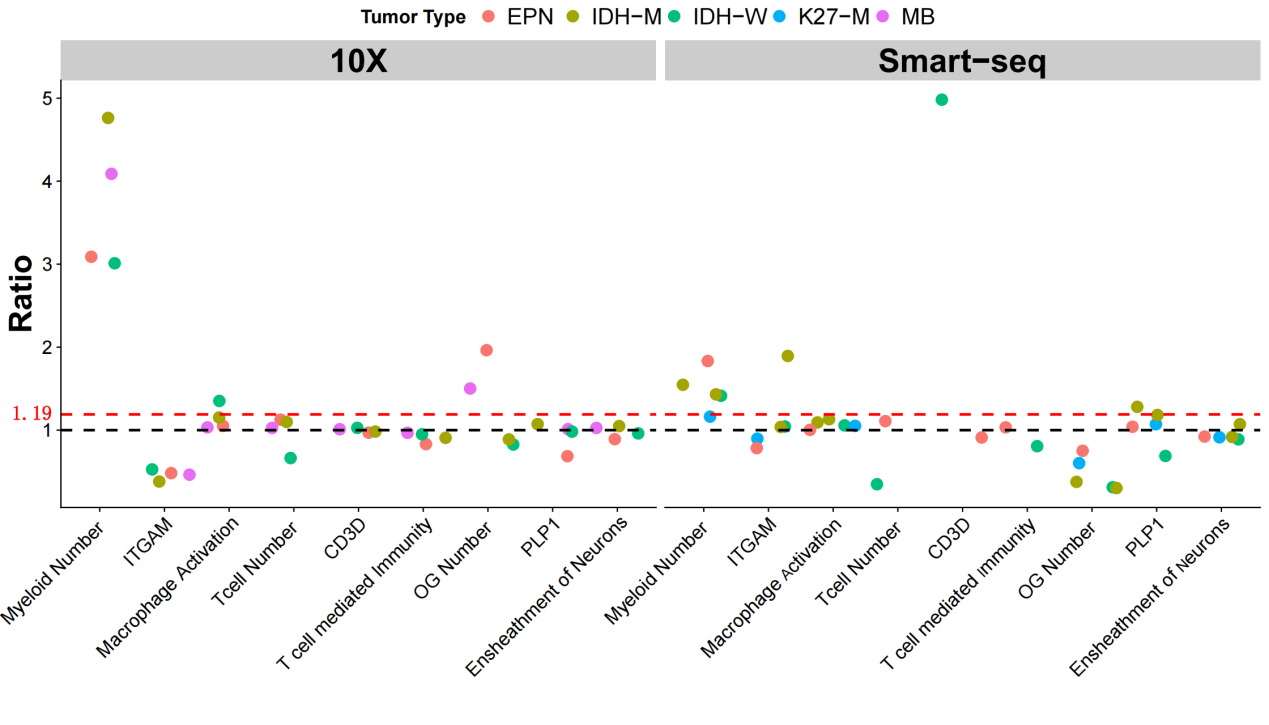
**

**Supplemental Figure 1.**The comparison of two methods-clustering-based (CB) method and positive expression of marker genes (PEMG) method. X axis represents the parameters of marker genes, cell types and corresponding marker pathways, and the y axis represents the ratio of the parameters between the two methods. The red line represents the mean ratio of all parameters.

**Supplemental Figure 2**


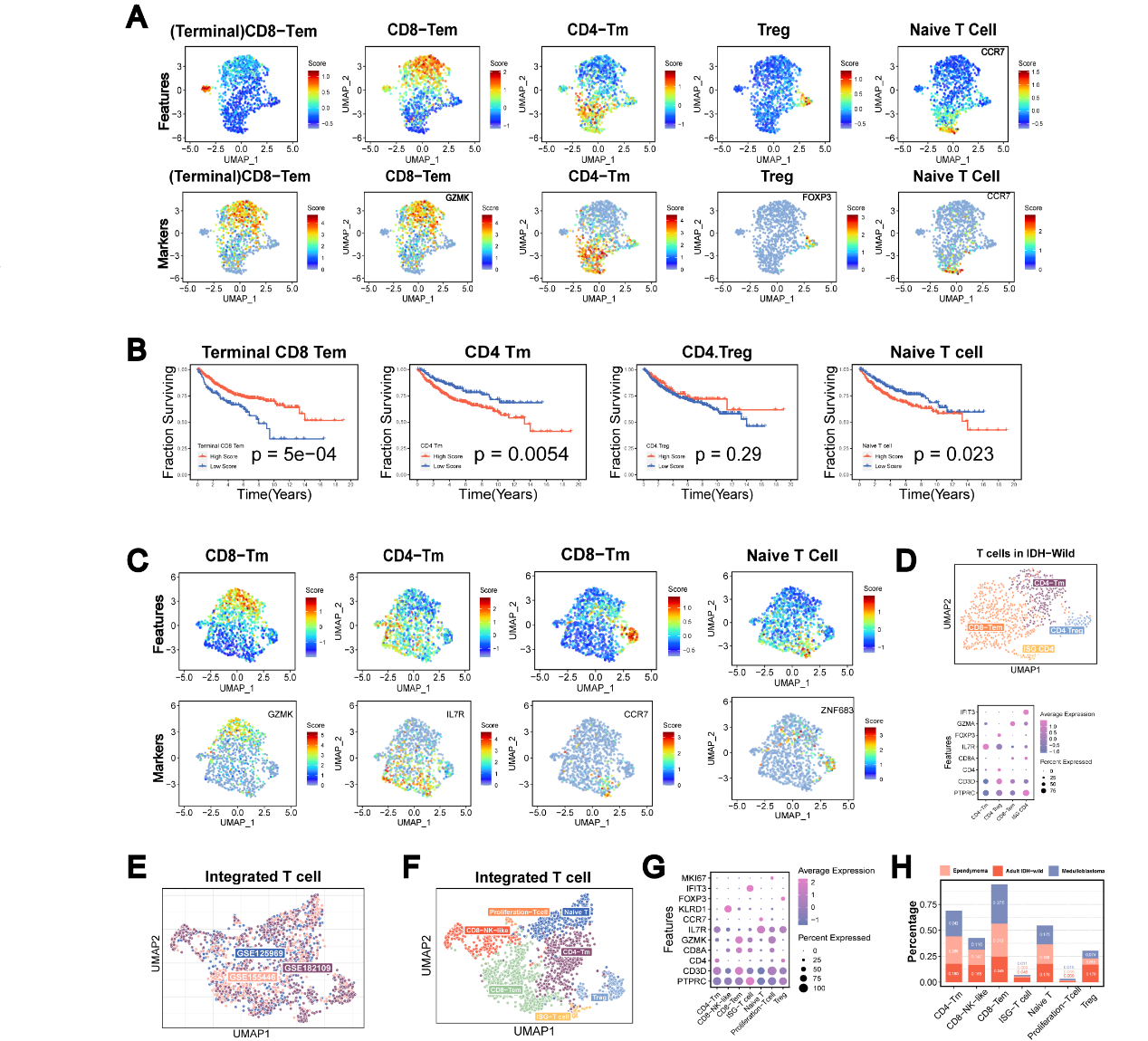


**Supplemental Figure 2. A**. UMAP overlays of the feature scores and marker genes expression level of T cell subpopulations in the single cell dataset of Medulloblastoma. **B.** Kaplan–Meier survival curves generated with signature score of each T cell subpopulation in the medulloblastoma. **C**. UMAP overlays of the feature scores and marker genes expression level of T cell subpopulations in the single cell dataset of ependymoma. **D.** Identification of T cell subpopulations in the adult IDH-wild glioma. UMAP shows the identified T cell subsets, and dotplot shows the marker genes of each subpopulations. **E.** The integration of T cells from the different datasets GSE155446 (medulloblastoma), GSE125969 (ependymoma) and GSE182109 (adult IDH-wild glioma). **F.** The T cell subpopulations identified in the integrated T cell dataset. **G.** The marker genes of each identified subpopulation of T cell. **H.** The comparison of percentages of T cell subpopulations among the different tumors.

**Supplemental Figure 3**


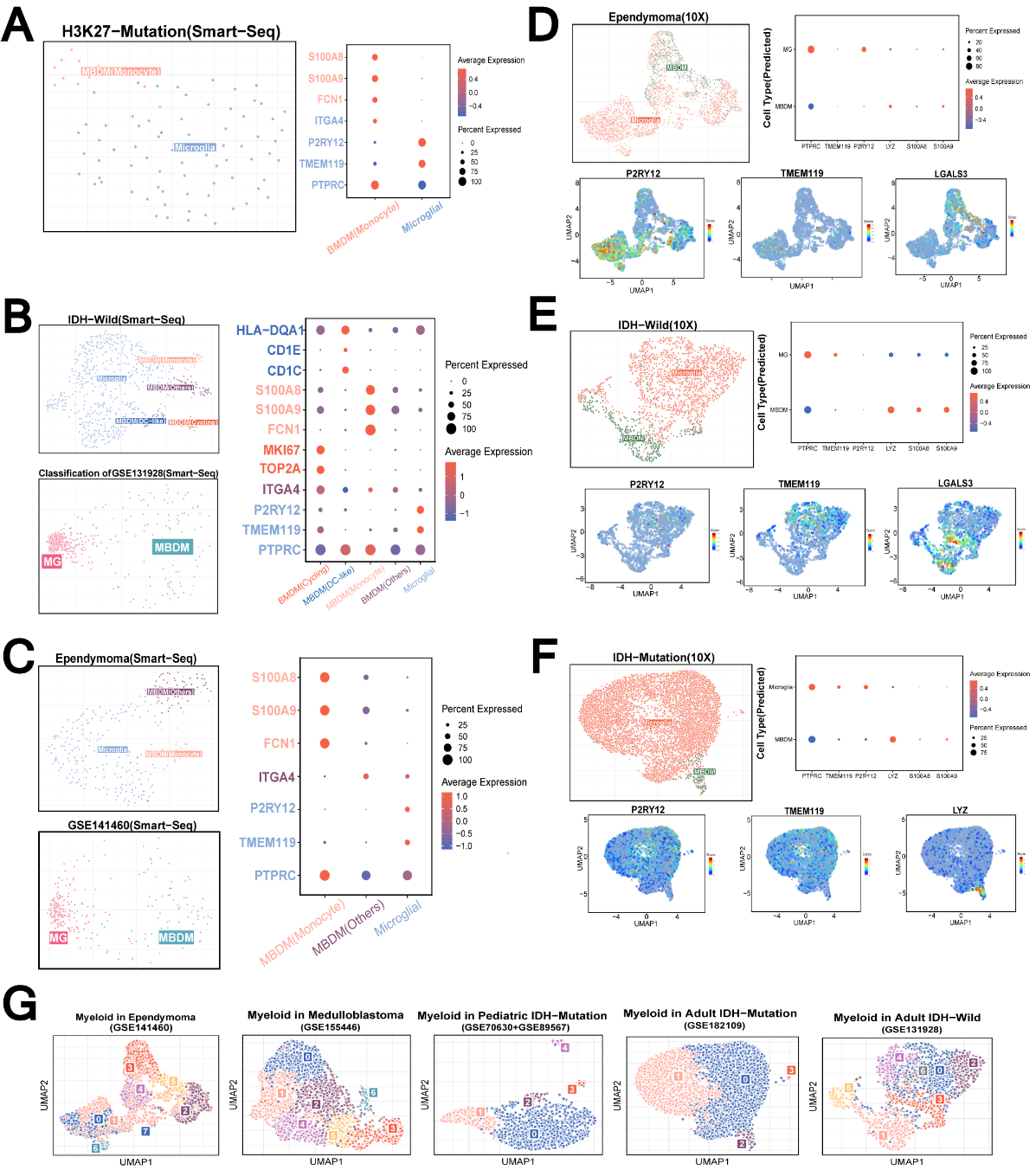


**Supplemental Figure 3. A.** The UMAP plot shows the clusters of myeloid compartments from the H3K27M-mutation based on smart-seq2. The dotplot displays the classical marker genes of MG and BMDM in each cluster. **B.** The UMAP plots respectively show the clusters of myeloid compartments from the adult IDH-wild gliomas based on smart-seq2 and displays the discrimination between MG and BMDM. The dotplot displays the classical marker genes of MG and BMDM in each cluster. **C.** The UMAP plots respectively show the clusters of myeloid compartments from the ependymoma based on smart-seq2 and displays the discrimination between MG and BMDM. The dotplot displays the classical marker genes of MG and BMDM in each cluster. **D-F.** Evaluation of MG and BMDM in the 10X data of medulloblastoma, adult IDH-wild glioma and IDH-mutation glioma. The UMAP plot displays the consistency between the predicted MG and BMDM and the clusters. The dotplot shows the expression of classical markers in the MG and BMDM. UMAP plots of marker genes shows the expression of classical markers in the cells. **G.** The UMAP plots shows the clusters of myeloid cells from the ependymoma, medulloblastoma, pediatric IDH-mutation glioma, adult IDH-mutation glioma and adult IDH-wild gliomas.

**Supplemental Figure 4**

**
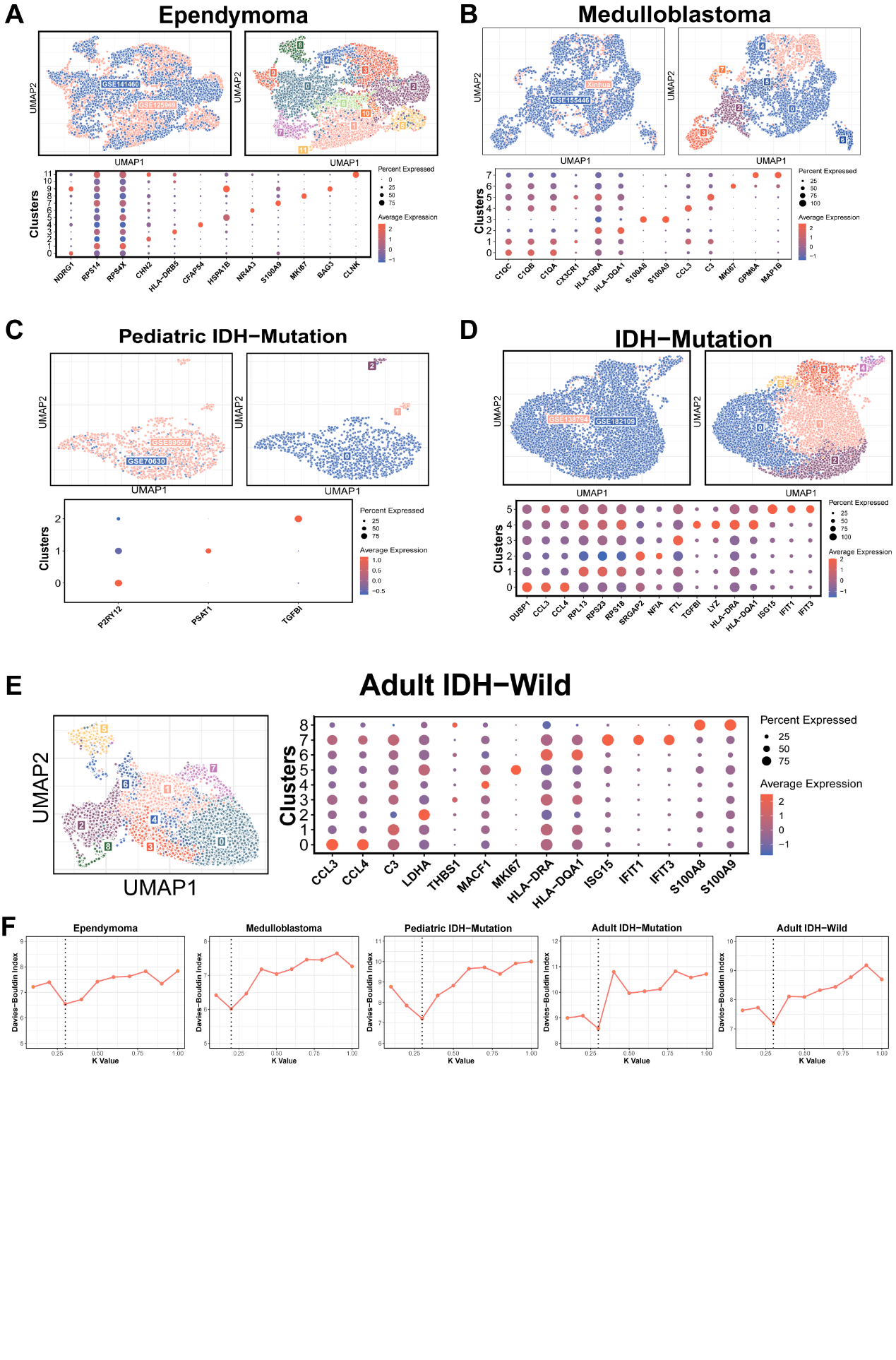
**

**Supplemental Figure 4.** **A-D.** The evaluation of myeloid subpopulations in ependymoma, medulloblastoma, pediatric IDH-mutation and adult IDH-mutation. The UMAP plots show the integration of the single cells from the different sources and the clusters of integrated myeloid datasets. The dotplots display the marker genes of each cluster. **E.** The UMAP plots shows the clusters of myeloid cells from the adult IDH-wild gliomas. **F.** The line charts show the optimal cluster number (dotted line) and the minimum value of Davies-Bouldin index (DBI) of different integrated myeloid datasets of each tumor type. X axis represents the cluster number, and Y axis represents the DBI.

**Supplemental Figure 5**

**
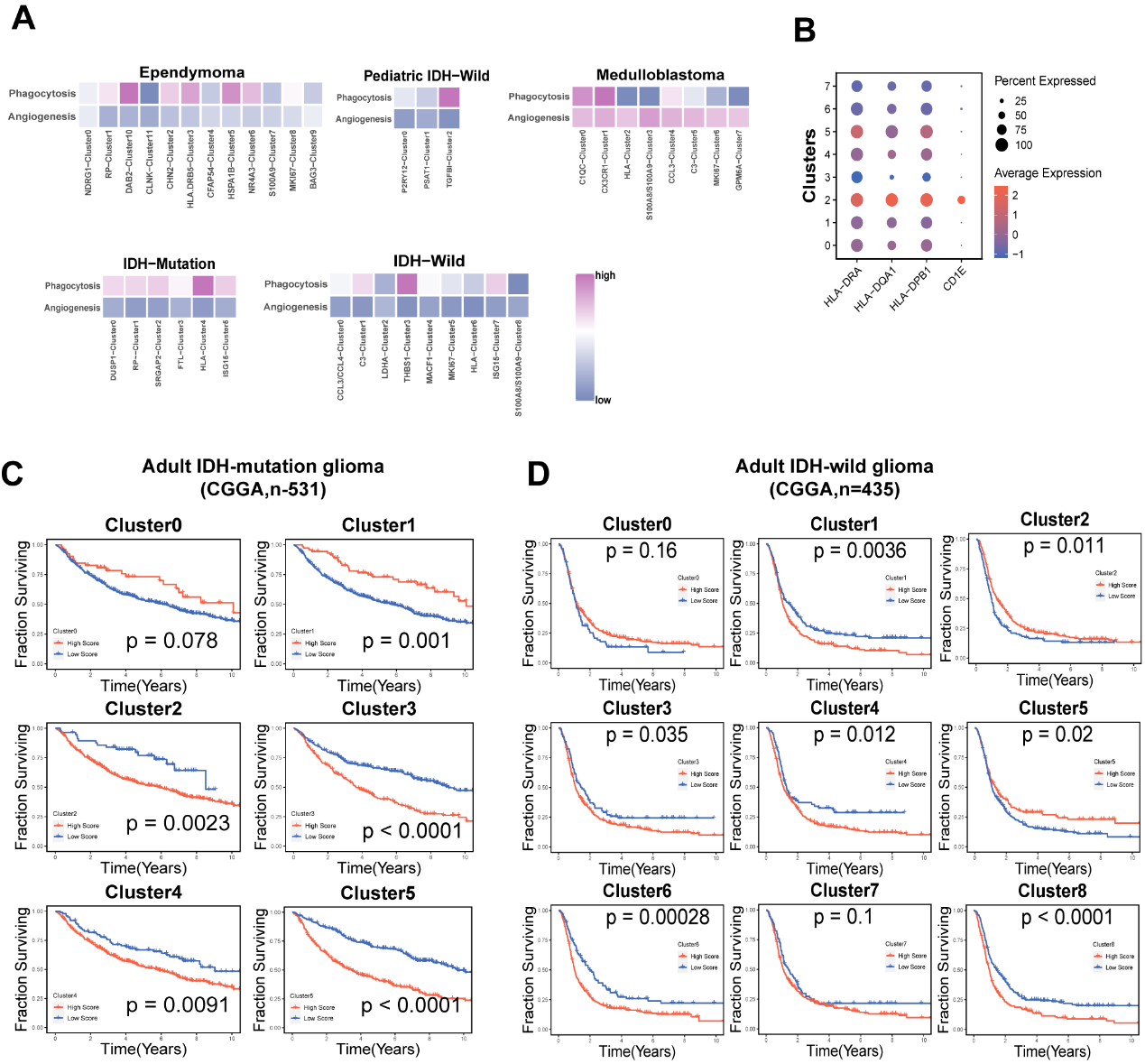
**

**Supplemental Figure 5. A.** Heatmap display the phagocytosis and angiogenesis scores in each cluster across the different tumors. **B.** Dotplot shows the marker genes (HLA-DQA1 and CD1E) in the cluster2 of medulloblastoma. **C.** Kaplan–Meier survival curves generated with each clusters signature score of adult IDH-mutation glioma using the CGGA dataset. **D.** Kaplan–Meier survival curves generated with each clusters signature score of adult IDH-wild glioma using the CGGA dataset.

**Supplemental Figure 6**

**
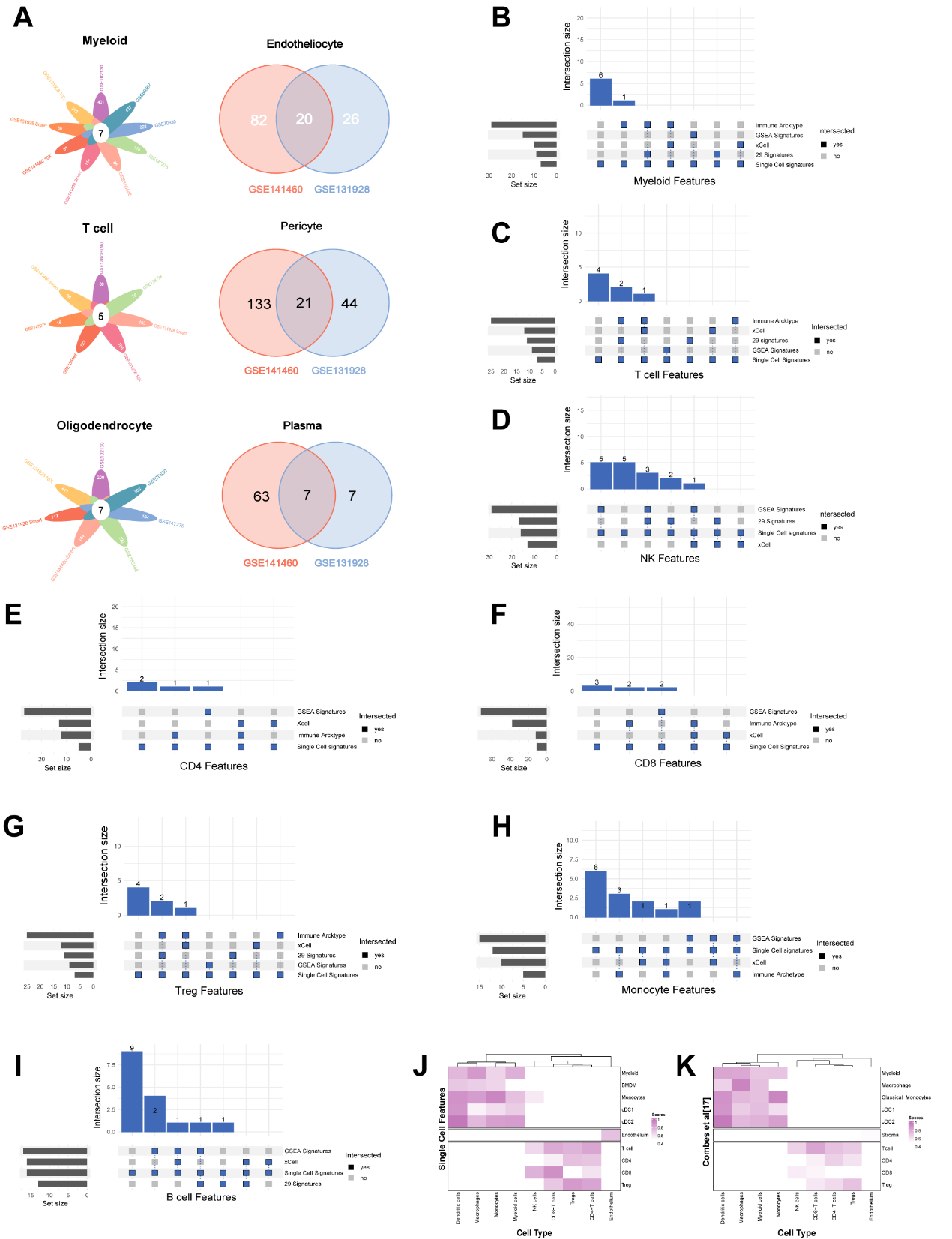
**

**Supplemental Figure 6. A.** The intersections of marker genes of major cell types across the different datasets. **B-I.** Upset plot reveals the intersections of gene features from scFes and other gene features from different sources**. J-K** Heatmap and hierarchical clustering of mean expression levels in the purified cell lines collected by previous study(Bagaev et al., 2021) .

**Supplemental Figure 7**

**
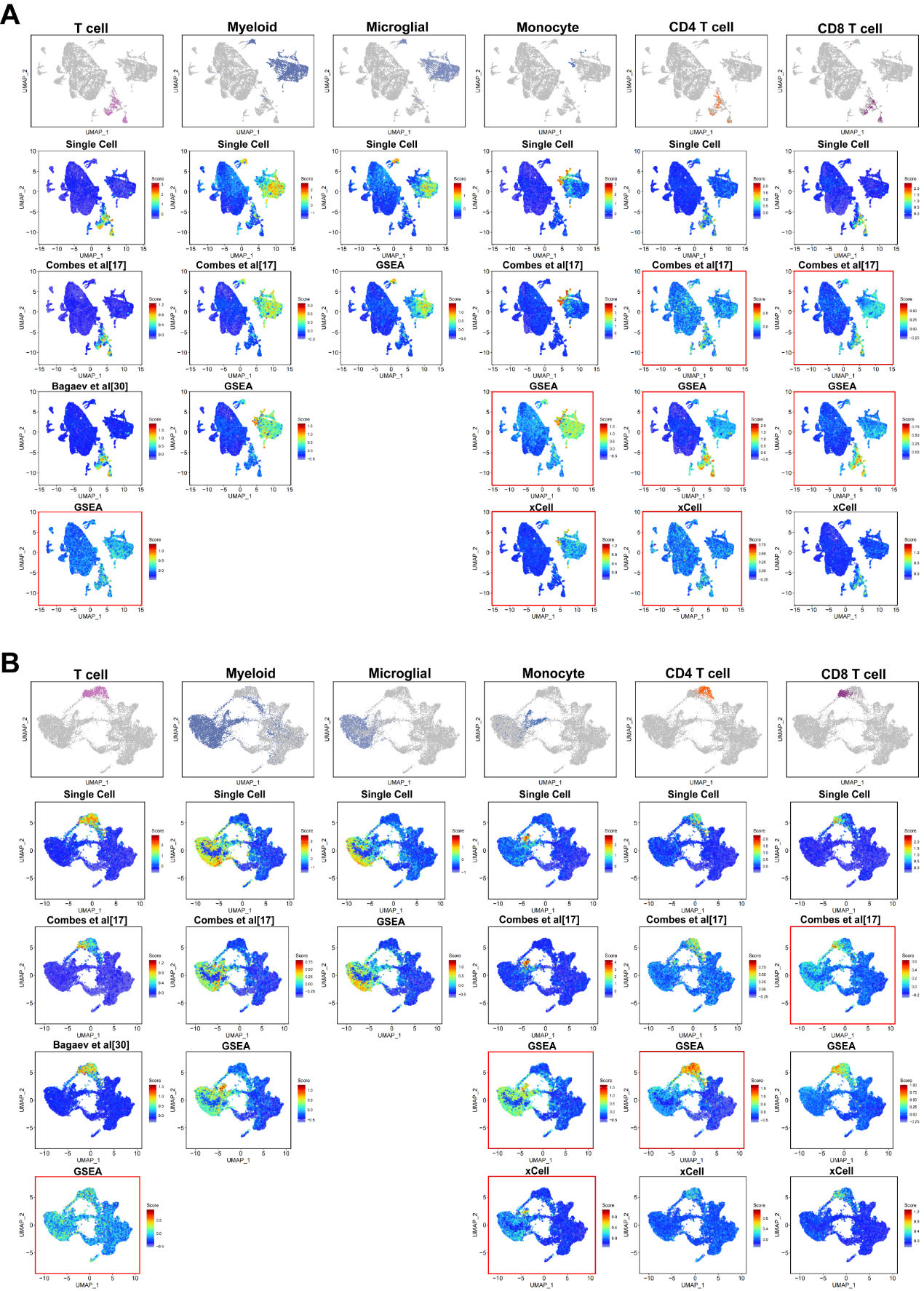
**

**Supplemental Figure 7.** A. UMAP overlays of the feature scores of major cell types in the independent single cell dataset of ependymoma. B. UMAP overlays of the feature scores of major cell types in the independent single cell dataset of adult IDH-wild glioma.

**Supplemental Figure 8**

**
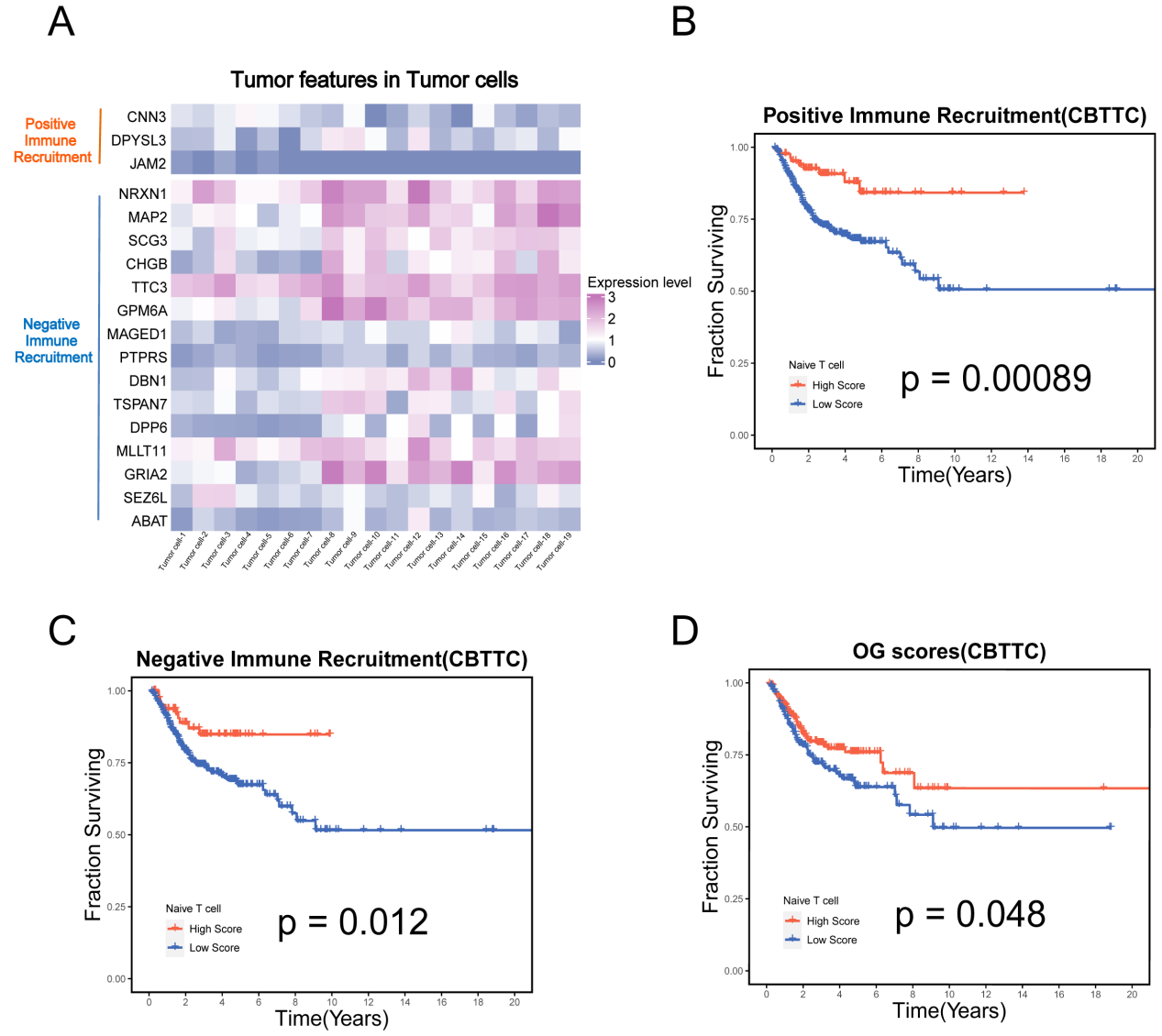
**

**Supplemental Figure 8. A.** The tumor cells in the medulloblastoma were sampled and the heatmap shows that the expression levels of negative-IR genes in the most of tumor cells are higher those of positive-IR genes. **B-D.** The positive-IP scores(**B**), the negative-IR scores(**C**) and OG(**D**) scores were associated with the better prognosis.

**Supplemental Figure 9**

**
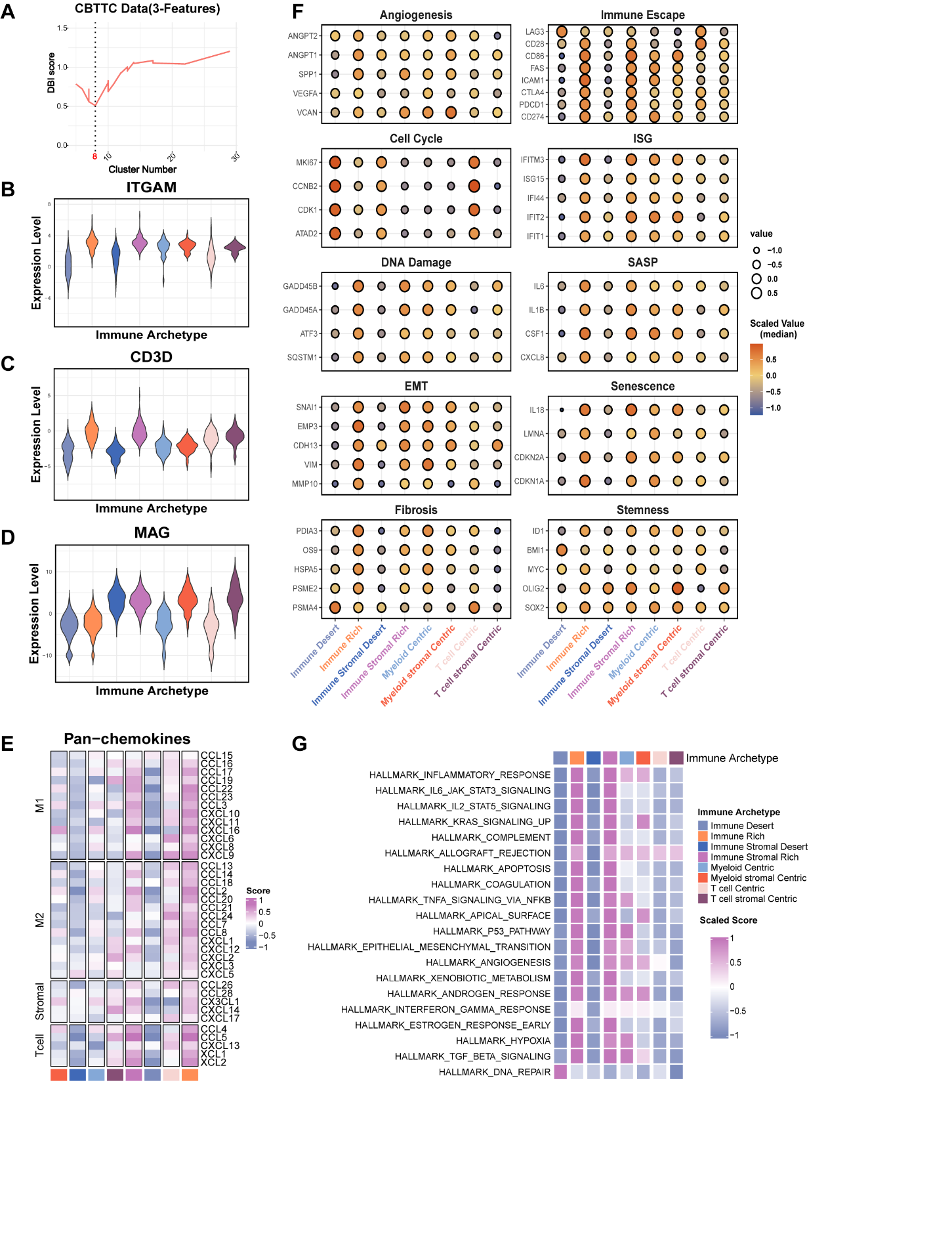
**

**Supplemental Figure 9. A.** The line chart shows optimal cluster number (dotted line) and the minimum value of DBI. X axis represents the cluster number, and Y axis represents the DBI. **B-D.** The violin plots respectively show the expression levels of three markers among the 8 clusters. **E.** Heatmap and hierarchical clustering of median chemokine gene expression per cluster in the CBTTC cohort. **F.** Dotplot shows the median chemokine gene expression per cluster of tumor related processes in the CBTTC cohort. **G.** A heatmap showing top and bottom Hallmark Pathways with the top 20 variances among the 8 clusters.

**Supplemental Figure 10**

**
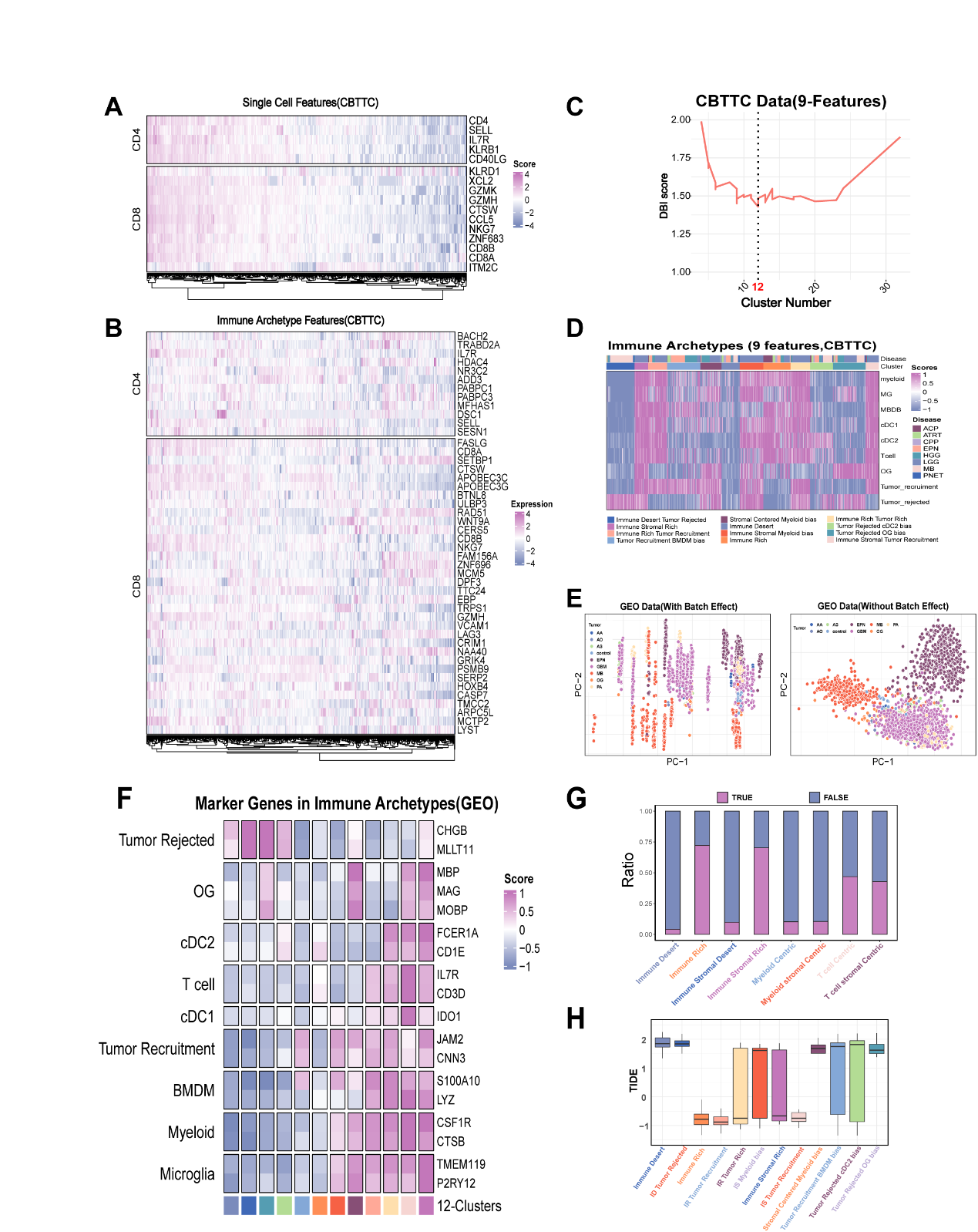
**

**Supplemental Figure 10. A**. Heatmap and hierarchical clustering of CD4 and CD8 features from scFes. **B.** Heatmap and hierarchical clustering of CD4 and CD8 features from the recently published article(引文). **C.** The line chart shows optimal cluster number (dotted line) and the minimum value of DBI. X axis represents the cluster number, and Y axis represents the DBI. **D.** The heatmap shows the 12 clusters based on the 9 features via hierarchical clustering. **E.** The PCA plot reveals the batch effect among the different RNA-seq datasets from GEO and the removal of batch effect with the preservation of corresponding tumor characters. **F.** The heatmap shows the mean expression levels of the marker genes of predominant cell type in each cluster using the integrated GEO dataset **G.** The prediction of response rates in each cluster (8 clusters). **H.** The TIDE score among the different clusters (12 clusters).
